# Toward an improved definition of a healthy microbiome for healthy aging

**DOI:** 10.1038/s43587-022-00306-9

**Published:** 2022-11-17

**Authors:** Tarini Shankar Ghosh, Fergus Shanahan, Paul W. O’Toole

**Affiliations:** 1 APC Microbiome Ireland, University College Cork, National University of Ireland, Cork, Ireland; 2 School of Microbiology, University College Cork, National University of Ireland, Cork, Ireland; 3 Department of Medicine, University College Cork, National University of Ireland, Cork, Ireland; 4 Present Address: Department of Computational Biology, Indraprastha Institute of Information Technology, New Delhi, India

**Keywords:** Microbiology, Computational biology and bioinformatics, Medical research, Ageing

## Abstract

The gut microbiome is a modifier of disease risk because it interacts with nutrition, metabolism, immunity and infection. Aging-related health loss has been correlated with transition to different microbiome states. Microbiome summary indices including alpha diversity are apparently useful to describe these states but belie taxonomic differences that determine biological importance. We analyzed 21,000 fecal microbiomes from seven data repositories, across five continents spanning participant ages 18–107 years, revealing that microbiome diversity and uniqueness correlate with aging, but not healthy aging. Among summary statistics tested, only Kendall uniqueness accurately reflects loss of the core microbiome and the abundance and ranking of disease-associated and health-associated taxa. Increased abundance of these disease-associated taxa and depletion of a coabundant subset of health-associated taxa are a generic feature of aging. These alterations are stronger correlates of unhealthy aging than most microbiome summary statistics and thus help identify better targets for therapeutic modulation of the microbiome.

## Abstract (web summary)

The authors analyze microbiome profiles from several public repositories to identify the higher-level indices that best reflect the abundance and ranking of disease-associated and health-associated gut microbes and that may help identify targets for therapeutic modulation.

## Main

Physical and cognitive decline with age is not experienced uniformly; delayed age-related decline (healthy aging) is evident in many people. One of the determinants of age-related decline is the microbiome. The microbiome transduces environmental signals that shape host immune, metabolic and neurologic function, and it modifies the risk of disease, including age-related diseases. However, the microbiome is, itself, modified by age-related impairment and age-related disease^1,2^. Several studies have found alterations in the composition and function of the microbiome as the host ages^1,3–6^ (reviewed also in Ghosh et al.^7^). We have also shown that age-related microbiome alterations are both distinct from and overlapping with those in age-related diseases^1,8^.

There is broad consensus how the microbiome changes with age, but specific intervention targets are less clear. Moreover, terms like diversity, assumed by many to be desirable^9^, and ‘uniqueness’, which has been cast as a marker of healthy aging^6^, need greater precision and should not be used agnostic of the loss or gain of specific taxa in aging. Other summary statistics include different measures of uniqueness that capture specific aspects of gut microbiome variability and are calculated using different distance measures.

Here, we analyzed microbiome diversity and four measures of microbiome uniqueness in 21,000 gut microbiomes for their relationship with aging and health. We show that diversity and uniqueness measures are not synonymous; uniqueness is not a uniformly desirable feature of the aging microbiome, nor is it an accurate biomarker of healthy aging. Different measures of uniqueness show different associations with diversity and with markers of health and disease. The Kendall uniqueness measure is negatively associated with microbiome diversity and health-associated taxa and positively associated with multiple disease-associated taxa. These health- and disease-associated taxa show the strongest association with the unhealthy aging phenotype and represent actionable targets for the design of microbiome-based therapeutics for older people.

## Results

### Uniqueness indices show different interactions with diversity

We analyzed 21,041 fecal microbiome datasets (or profiles) from seven data repositories (Methods and Table 1)^1,10–16^. Six of these data repositories covered participants from Europe, North/South America, Asia and Africa and ranging from 18 to 100 years old. One repository (NU-AGE) was specific to older individuals^15^. The combined study population derives from 19 nationalities across Europe, North America, South America, Asia, Pacific Islands and Africa. Taxonomic profiles at the genus and species level and MetaCyc-based functional profiles were also available for all the 8,430 Shotgun datasets included in this study (Table 1).

**Table 1:** Description of datasets corresponding to the 21,041 gut microbiomes from the seven data repositories

Study name	Investigation type	Data repository	Data type	Nationality	Region	Minimum age	Maximum age	Total samples			Percentage of samples (adults age >60 years)	Study conditions	Accession number
								Total	Controls	Patients			
HMP_2019_ibdmdb	I	CMD3	Shotgun	USA	EU/NA	18	76	846	219	627	13.83	IBD, control	PRJNA389280
SankaranarayananK_2015	I	CMD3	Shotgun	USA	EU/NA	20	84	37	18	19	24.32	Control, T2D	PRJNA268964
CosteaPI_2017	I	CMD3	Shotgun	DEU, KAZ	EU/NA	19	75	201	201	0	16.92	Control	PRJEB17632
AsnicarF_2021	I	CMD3	Shotgun	USA, GBR	EU/NA	18	65	1,098	1,098	0	11.57	Control	PRJEB39223
HansenLBS_2018	I	CMD3	Shotgun	DNK	EU/NA	22	65	207	207	0	16.91	Control	PRJNA491335
NielsenHB_2014	I	CMD3	Shotgun	DNK, ESP	EU/NA	18	70	394	247	147	17.26	Control, IBD	PRJEB1220
SchirmerM_2016	I	CMD3	Shotgun	NLD	EU/NA	18	75	465	465	0	6.02	Control	PRJNA319574
WirbelJ_2018	I	CMD3	Shotgun	DEU	EU/NA	28	90	125	65	60	53.60	CRC, control	PRJEB27928
ZellerG_2014	I	CMD3	Shotgun	FRA	EU/NA	25	89	156	61	95	69.87	Control, CRC, polyps	PRJEB6070
KeohaneDM_2020	I	CMD3	Shotgun	IRL	EU/NA	18	72	117	117	0	3.42	Control	PRJEB36820
QinN_2014	I	CMD3	Shotgun	CHN	East_Asia	18	78	237	114	123	13.08	Control, cirrhosis	PRJEB6337
YeZ_2018	I	CMD3	Shotgun	CHN	East_Asia	19	75	65	45	20	7.69	Control, BD	PRJNA31482
QinJ_2012	I	CMD3	Shotgun	CHN	East_Asia	19	86	342	173	169	20.76	Control, T2D	PRJNA42234
YachidaS_2019	I	CMD3	Shotgun	JPN	East_Asia	21	79	616	251	365	63.80	CRC, control, polyps	PRJDB4176
DhakanDB_2019	I	CMD3	Shotgun	IND	South_Asia	19	71	88	88	0	7.95	Control	PRJAN397112
GuptaA_2019	I	CMD3	Shotgun	IND	Pacific/SA	22	75	60	30	30	31.67	CRC, control	PRJNA531273
BritoIL_2016	I	CMD3	Shotgun	FJI	Pacific/SA	20	80	154	154	0	24.68	Control	PRJNA217052
PehrssonE_2016	I	CMD3	Shotgun	PER, SLV	Africa	18	84	120	120	0	17.50	Control	PRJNA300541
LokmerA_2019	I	CMD3	Shotgun	CMR	Africa	26	78	57	57	0	29.82	Control	PRJEB27005
PasolliE_2019	I	CMD3	Shotgun	MDG	Africa	21	72	111	111	0	1.80	Control	PRJNA485056
RubelMA_2020	I	CMD3	Shotgun	CMR	Africa	18	87	156	79	77	16.67	STH, control	PRJNA547591
RampelliS_2015		CMD3	Shotgun	TZA, ITA	Africa	20	70	33	33	0	6.06	Control	PRJNA278393
AG	I	AGP	16S	USA, GBR	EU/NA	18	101	3,812	2,404	1,408	26.84	Alzheimer’s disease, ASD, cancer, CVD, CDI, diabetes, IBD, IBS, kidney disease, liver disease, lung disease, migraine, SIBO	PRJEB11419
NU-AGE	I	NU-AGE	16S	EU	EU/NA	65	79	464	333	131	43.53	Controls	This Study
ISC	I	ISC	Shotgun	IRL	EU/NA	17	102	610	610	0	100.00	Control, longstay (long-term residential care), IBS	PRJEB37017, PRJEB42304, PRJEB20054, PRJEB15388
He	I	HE	16S	CHN	East_Asia	18	97	7,009	3,559	3,450	0.35	Atherosclerosis, cholecystitis, colitis, constipation, diarrhea, fatty liver, gastritis, IBS, kidney stone, rheumatoid arthritis, metabolic syndrome, T2D	PRJEB18535
Odamaki	I	Odamaki	16S	JPN	East_Asia	18	104	306	306	0	37.91	Controls	PRJDB4360
LogMPie	I	LOGMPIE	16S	IND	South_Asia	18	65	874	874	0	5.84	Controls	PRJEB25642
FengQ_2015	II	CMD3	Shotgun	AUT	EU/NA	43	86	154	61	93	86	CRC, polyps	PRJEB7774
HanniganGD_2017	II	CMD3	Shotgun	CAN, USA	EU/NA	35	88	81	28	53	47	Polyps, CRC	PRJNA389927
ThomasAM_2018a	II	CMD3	Shotgun	ITA	EU/NA	49	84	80	24	56	80	Polyps, CRC	PRJNA447983
ThomasAM_2018b	II	CMD3	Shotgun	ITA	EU/NA	38	70	59	27	32	47	CRC	PRJEB27928
ThomasAM_2019_c	II	CMD3	Shotgun	JPN	East_Asia	28	78	80	40	40	62	CRC	DRA006684
VogtmannE_2016	II	CMD3	Shotgun	USA	EU/NA	31	89	104	52	52	62	CRC	ERR1293500
YuJ_2015	II	CMD3	Shotgun	CHN	East_Asia	34	89	128	53	75	71	CRC	PRJEB10878
Heitz-BuschartA_2016	II	CMD3	Shotgun	LUX	EU/NA	19	62	35	16	19	17	T1D	PRJNA289586
HMP_2019_t2d	II	CMD3	Shotgun	USA	EU/NA	33	69	296	46	250	57	IGT, T2D	https://portal.hmpdacc.org*
KarlssonFH_2013	II	CMD3	Shotgun	EU	EU/NA	68	71	145	43	102	100	IGT, T2D	PRJEB1786
JieZ_2017	II	CMD3	Shotgun	CHN	East_Asia	32	107	378	164	214	56	ACVD	PRJEB21528
NagySzakalD_2017	II	CMD3	Shotgun	USA	EU/NA	51	51	100	50	50	0	ME/CFS	PRJNA379741
VincentC_2016	II	CMD3	Shotgun	CAN	EU/NA	61	91	229	196	33	100	CDI	PRJNA297252
XieH_2016	II	CMD3	Shotgun	GBR	EU/NA	36	80	250	177	73	64	Migraine, asthma	PRJEB9576
ZhuF_2020	II	CMD3	Shotgun	CHN	East_Asia	18	64	162	81	81	1	Schizophrenia	PRJEB29127
Total samples								21,041					

The table shows the descriptions of the two kinds of datasets (study cohorts) used in the current study. Type I study cohorts were included for age–microbiome investigations. Overall, the total number of microbiome profiles investigated here was 18,760 from 28 studies with ~ 7,000 gut microbiome profiles from older people (≥60 years). Type II study cohorts were those that were additionally investigated only for the disease–microbiome interactions in older (≥60 years) and young/middle-aged (<60 years) participants. These included a total of 2,281 gut microbiomes from 15 studies. The public accession numbers of the sequence datasets corresponding to each of the 43 individual studies are also indicated.

Country abbreviations: CHN, China; GBR, Great Britain; CAN, Canada; USA, United States of America; DEU, Germany;

FRA, France; DNK, Denmark; LUX, Luxembourg; JPN, Japan; ITA, Italy; AUT, Austria; IND, India; TZA, Tanzania; CMR, Cameroon; MDG, Madagascar; PER: Peru; SLV, El-Salvador; FJI, Fiji; NLD, Netherlands; ESP, Spain; KAZ, Kazakhstan.

Disease abbreviations: IBD, inflammatory bowel disease; T1D, type 1 diabetes; T2D, type 2 diabetes; CRC, colorectal cancer; STH, soil-transmitted helminths; BD, Bechet’s disease; ASD, autism spectrum disorder; CVD, cardiovascular disease; CDI, *Clostridioides difficile* infection; IBS, irritable bowel syndrome; SIBO, small intestinal bacterial overgrowth; IGT, impaired glucose tolerance; ME/CFS, myalgic encephalomyelitis/chronic fatigue syndrome; ACVD, atherosclerotic cardiovascular disease.

**Extended Data Fig. 1:**
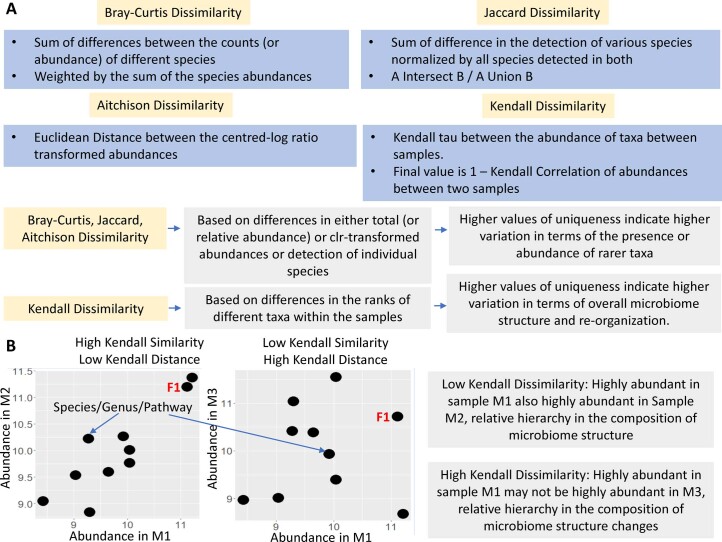
Different uniqueness measures and their implications. **A**. Summary description of the inter-microbiome distance calculation using the four different distance metrics and the aspects of gut microbiome variation captured by the different distance matrices. **B**. Pictorial illustration of the meaning of high or low Kendall similarity measures using three different hypothetical microbiomes (M1, M2 and M3). While Microbiomes M1 and M2 have a high Kendall Similarity, M1 and M3 do not. The two sub-plots pictorially elaborate this. Each point in the two sub-plots represents a feature which may be either the abundance of a species or genus or pathway. In the left sub-plot, the y-value of each represents the abundance of the corresponding features in M2 and the x-value represents the abundance of the same feature in M1. In the right sub-plot, the y-value of each point represents the abundance of the corresponding features in M3 and the x-value represents the abundance of the same feature in M1. As observed, in the left sub-plot of higher Kendall similarity (and low Kendall distance), highly abundant features in M1 (for example the feature F1 highlighted in red) are also highly abundant in M2. Thus, the ranking of the features in terms of their abundance in M1 and M2 are similar. This is not observed between M1 and M3 which have lower Kendall similarity and higher Kendall distance.

We first calculated five microbiome summary statistics, Shannon index (or diversity) and four different measures of uniqueness, namely, Bray–Curtis (as used by Wilmanski et al.^6^), Jaccard, Aitchison and Kendall (Methods) at the levels of genus, species and functional pathways (MetaCyc). Higher values of Bray–Curtis, Jaccard or Aitchison uniqueness indicate greater variation in the presence or abundance of taxa (or pathways). In contrast, higher Kendall uniqueness indicates higher variation of overall microbiome structure and reorganization (Extended Data Fig. 1). We then investigated the associations between these properties within each individual study cohort and across all studies (Supplementary Fig. 1).

**Extended Data Fig. 2:**
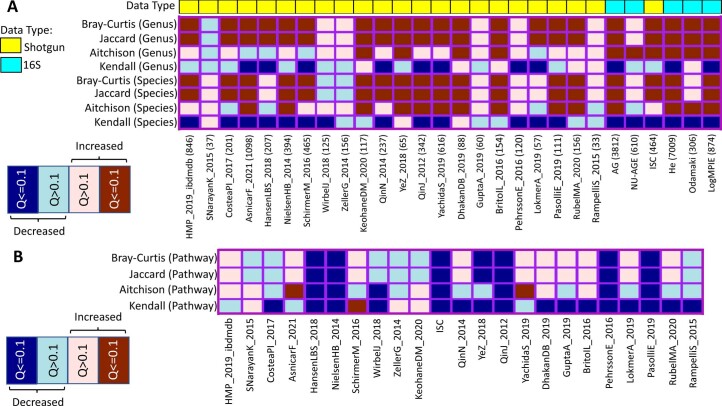
Links between diversity and uniqueness. Heatmaps showing the association patterns between Shannon Diversity and the four measures of uniqueness at the level of: A. Species and Genus and B. Pathways. Each cell indicates the directionality and the strength between diversity and specific measure of uniqueness (given by the row) in a specific study (given by the column). The strengths are indicated in the legend key on the left. The number of gut microbiomes investigated in each study cohort is shown in parentheses for the corresponding studies for the top heatmap. The statistical significance of the associations were computed using two-sided robust F-tests. The p-values obtained for the association of the different microbiome summary indices were corrected on a per-study cohort basis using Benjamini–Hochberg correction to compute the *Q*-values. Source data

Different measures of uniqueness were mutually positively correlated to varying extents across studies but showed different relationships with microbiome diversity (Shannon index) (Supplementary Tables 1 and 2, Extended Data Fig. 2 and Supplementary Fig. 2). Although Bray–Curtis, Jaccard and Aitchison uniqueness values (all associated with increased abundance and detection of rarer taxa) positively associated with diversity, the Kendall uniqueness measure (which reflects differences in overall microbiome hierarchy and relative rank abundance of individual microbiome members) showed significantly negative correlation with Shannon diversity across most datasets (Extended Data Fig. 2 and Supplementary Fig. 2). This differential association of the uniqueness measures with Shannon diversity was consistent at both species and genus levels. Kendall uniqueness and diversity were also negatively associated at the level of functional pathways (Extended Data Fig. 2, Supplementary Fig. 2b and Supplementary Table 1b). Thus, higher values of Kendall uniqueness, associated with loss of gut microbiome organization, occur when there is a loss of structure and diversity of the overall gut microbiome.

### Uniqueness and diversity show a geography-specific increase with age

**Fig. 1:**
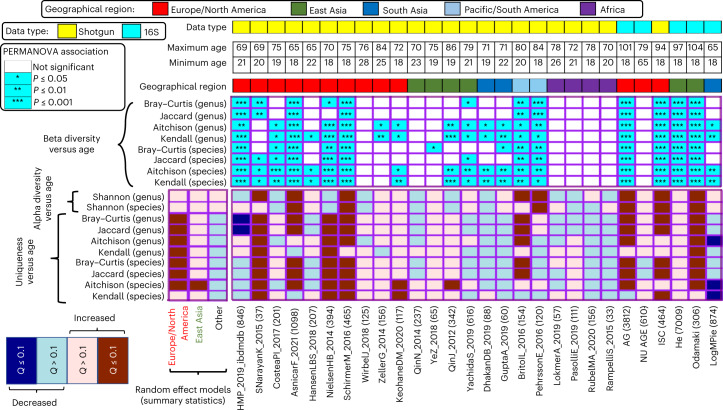
Association of measures of microbiome uniqueness, Shannon diversity and beta diversity with age in different study cohorts shows region-specific variabilities. The names of the study cohorts appear as listed in Table 1, and the number of investigated gut microbiomes are indicated in parentheses. The top four rows indicate the data type (Shotgun or 16S; as per legend), maximum and minimum participant age and the geographical region. The heatmap immediately below these panels shows the results of PERMANOVA for associating overall beta diversity with age computed using the four microbiome distance matrices analyzed at the levels of genus and species. The bottom heatmap shows the results of the robust linear regression models for associating species and genus-level microbiome summary statistics with age across the different individual studies. The statistical significance of the associations were computed using two-sided robust *F*-tests. The *P* values obtained for the association of the different microbiome summary indices were corrected on a per-study cohort basis using Benjamini–Hochberg correction to compute the *Q*-values. Also indicated on the right of this heatmap are the results of the association meta-analyses of these microbiome summary statistics with age for studies grouped based on their geographical regions. For a given geographical region, the summarized associations are computed using random effect models on the specific individual study-specific effect sizes (computed based on robust linear regression models (Methods)). As for the previous heatmap, the *P* values obtained for the association of each summary index were corrected on for each geography-specific study groups using Benjamini–Hochberg corrections. The results show that age-wise association of the gut microbiome with age (association of individual summary statistics as well as overall diversity) shows region-specific signatures, with the strongest effects being observed for the European and North American cohorts. Various measures of uniqueness and diversity strongly associate with age, but only for the European and North American cohorts. Source data

**Extended Data Fig. 3:**
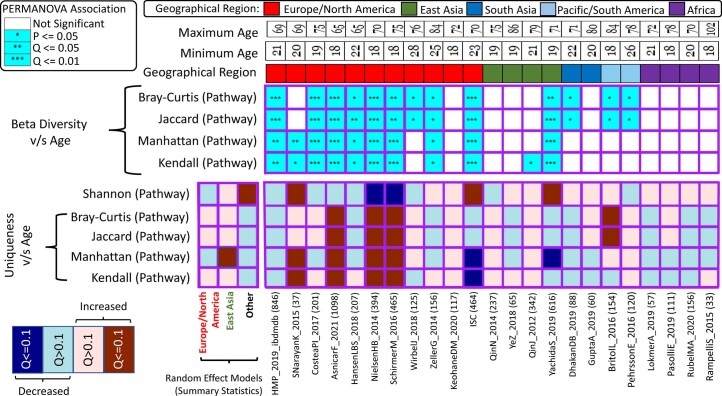
Association of different measures of Uniqueness, Shannon diversity and beta diversity with age in different study cohorts at the functional pathway-level using the MetaCyc scheme. The names of the study cohorts (with the number of investigated gut microbiomes indicated in parentheses) are indicated. The top three rows indicate the maximum participant age, the minimum participant age and the geographical region of the study cohorts (indicated in different colors as provided in the legends). The heatmap immediately below these panels shows the results of PERMANOVA for associating overall pathway-level beta-diversity with age computed using the four different microbiome distance matrices (utilized in the current study). The bottom heatmap shows the results of the robust linear regression models for associating various pathway-level microbiome summary statistics with age across the different individual studies. The statistical significance of the associations were computed using two-sided robust F-tests, corrected on a per-study cohort basis using Benjamini–Hochberg corrections to obtain *Q*-values. Also indicated on the right of this heatmap are the results of the association meta-analyses of these microbiome summary statistics with age for studies grouped based on their geographical regions. For a given geographical region, the summarized associations are computed using Random Effect Models on the specific individual study-specific effect sizes (computed based on robust linear regression models (See Methods). Here, the p-values were computed using two-sided permutation tests corrected per-geography-specific-cohort groups using Benjamini–Hochberg corrections to obtain *Q*-values. Source data

Because the microbiome is affected by age and geography, we investigated the separate and combined interaction of these variables with age (Fig. 1, Extended Data Fig. 3 and Supplementary Table 3). Overall, in 21 of 28 datasets (~75%) subjected to diversity or uniqueness analysis at the taxonomic level (Fig. 1), and in 15 of 23 datasets (~65%) examined at the pathway level (Extended Data Fig. 3), we detected significant association (*P* < 0.05) between age and overall microbiome (beta) diversity for at least one of the distance measures. This finding indicated that for a majority of studies, the gut microbiome composition changed with age. The individual distance measures did not show any consistent differences in the number of their associations. Notably however, the association of overall gut microbiome composition with age was strongest in European and North American individuals (consistently significant associations with multiple beta-diversity measurements) (Fig. 1). This pattern was even stronger for pathway beta-diversity analysis (Extended Data Fig. 3), where microbiome function significantly associated with age in 10 of 11 European/North American cohorts. In contrast, we observed significant association between pathway data and age in only 4 out of the 13 cohorts from other geographies.

**Extended Data Fig. 4:**
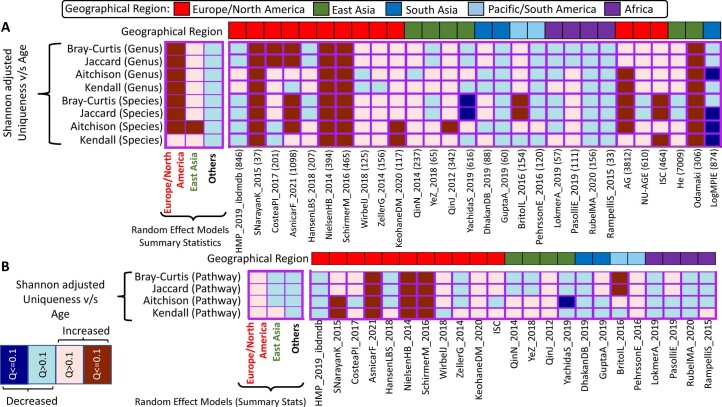
Association between Shannon-adjusted uniqueness with age. Heatmaps showing the association between Shannon-adjusted measures of uniqueness and age across A) the 28 studies (with taxonomic profile) and B) the 23 studies with pathway profiles, obtained using Study-Specific Robust Linear Regression models. The geographic origin of each study is also indicated. Also indicated are the results of the summarized associations obtained by performing Random Effect Models analysis of these individual study associations by grouping cohorts into different geographical bins. The number of gut microbiomes investigated in each study cohort is shown in parentheses for the corresponding studies for the top heatmap. Source data

Multiple measures of uniqueness and diversity also positively correlated with age, but like beta diversity, primarily for European and North American individuals (Fig. 1; Supplementary Table 4 individual studies; Supplementary Table 3 Random Effects Model for cohort geographies). This was similar to the positive association between Bray–Curtis uniqueness and age in a predominantly North American study population reported by Wilmanski et al.^6^. However, across datasets from other geographies, neither uniqueness nor diversity associated with age (Fig. 1 and Supplementary Table 4). Using random effect models against geography-specific study groups, the positive association of multiple measures of uniqueness and diversity with age shifted from being strongly or significantly positive for Europeans and North Americans to being nonsignificant for other geographies (Supplementary Table 5). This pattern was especially pronounced at the taxonomy level. Overall, these strong differences in the age-associated alterations in the gut microbiome were not associated with either study sample size, or the cohort size of older adults, or age-range (Supplementary Fig. 3). However, for African cohorts, microbiomes from older adults were underrepresented (numbers ranging from 2 to 26). Given the strong association between diversity and uniqueness measures, we recomputed the associations between aging and uniqueness measures after adjusting for the Shannon diversity across all studies (for both taxonomy and function). The patterns remained largely unchanged (Supplementary Tables 6 and 7 and Extended Data Fig. 4).

Thus, the extent and type of age-specific microbiome associations (including summary indices) differ with geography. Most of the exceptions in Europe to these data interactions were in the NU-AGE cohort, perhaps because of the narrow age range of this targeted-recruitment cohort (Fig. 1).

### Summary indices show different links with disease-/health-linked taxa

We next tested if microbiome summary statistics reflected the abundances of taxa consistently reported as showing differential associations with health. We focused on 107 species-level taxa present in at least 5% of the microbiomes, in at least 60% of studies, in both Shotgun-derived and 16S-datasets. We primarily investigated compositionality-tuned clr-transformed taxonomic abundances, although pilot evaluation of different normalization strategies identified very similar taxon abundances (Methods and Supplementary Figs. 4 and 5).

**Fig. 2:**
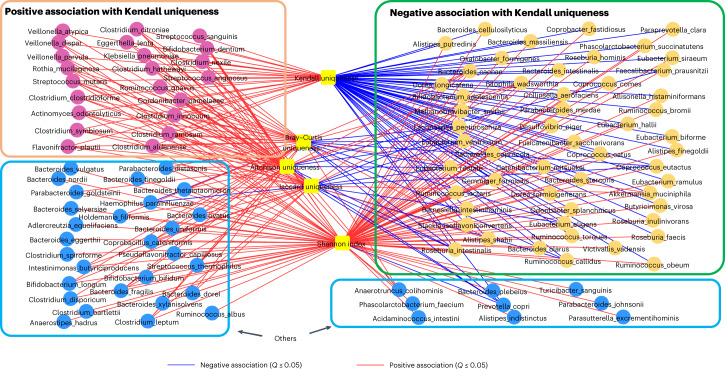
Identification of species-level groups based on their pattern of association with different microbiome summary statistics. Species fall into three groups based on their association pattern with Kendall uniqueness. Each edge indicates an association with *Q* ≤ 0.05, with colors blue and red indicating significant negative and positive associations, respectively. Based on their pattern of association, the microbiome taxa can be resolved into three partitions based on their association with Kendall uniqueness. A set of 54 species-level taxa containing many of the putatively beneficial symbionts show significantly negative association with Kendall uniqueness. A group of 22 species-level taxa containing many taxa previously shown to be associated positively with multiple diseases/unhealthy measures, like frailty^1^, associate positively with Kendall uniqueness. The disease/unhealthy aging links of the above two groups are further validated in Figs. 3 and 5. A third group of 36 taxa (highlighted as ’Others’) show no association with Kendall uniqueness. Source data

**Extended Data Fig. 5:**
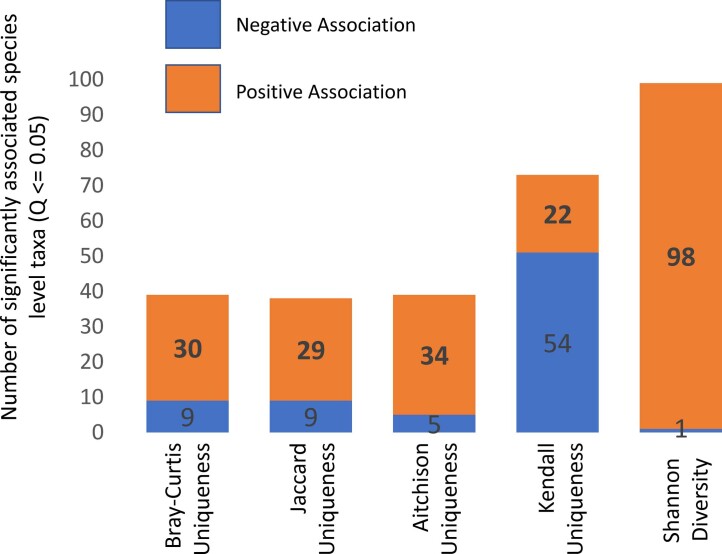
Overall directionalities of association between different summary indices and microbiome taxa. Stacked bar plots showing the number of species-level taxa that show either significantly positive or significantly negative associations with each microbiome summary statistics in the Random Effect Models based meta-analysis across all the 28 studies. 107 species-level taxa that we detected in at least 5% of the microbiomes, in at least 60% of the studies in both Shotgun and 16S groups of datasets. Source data

We detected 288 significant associations between these 107 species and the five microbiome summary statistics (Methods, Supplementary Tables 8 for cohort-specific associations, Supplementary Table 9 and Extended Data Fig. 5 for across cohort meta-analysis using random effect models). The maximum number of associations were with the Shannon index (99 associations) and Kendall uniqueness (76 associations) (Extended Data Fig. 5). However, the pattern of these associations was different. Although almost all associations obtained with the Shannon diversity index were positive (98 of 99), the associations with Kendall uniqueness included both negative (54 associations) and positive associations^17^. The individual species-level taxa (see heatmap in Supplementary Fig. 6) clustered into three groups (Fig. 2) comprising 54 species-level taxa negatively associated with Kendall uniqueness, 22 species-level taxa positively associated with Kendall uniqueness and 37 species-level taxa showing no associations with Kendall uniqueness. We referred to these groups of taxa as Kendall uniqueness negative, Kendall uniqueness positive and others, respectively.

**Extended Data Fig. 6:**
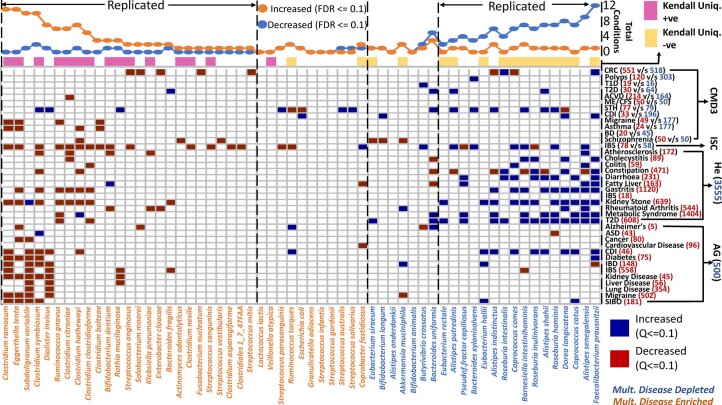
Identification of taxa enriched or depleted in multiple diseases in 12,000 gut microbiomes. Disease associations (where disease metadata were available) were tested based upon whether or not these taxa replicated the multiple-disease-enriched and the multiple-disease-depleted taxa previously identified in Ghosh *et al*.^1^ which was based on 2,500 microbiome profiles that were not included in the current analysis. The results of the association analysis are shown in a single heatmap for both Shotgun (CMD3 and ISC) 16S-based (AG and He) datasets. For AG and He (where control and patient gut microbiomes were sequenced as part of a single study), we compared taxon abundances in patients (numbers of metagenomes in red; right panel) versus controls (numbers in blue besides repository names) from the same data repository using two-sided Mann–Whitney tests. For CMD3 and ISC, we used matched patient-control studies pertaining to each disease. The number of patient and control gut microbiomes being compared are indicated in the right panel in parenthesis corresponding to each disease (in red and blue font, respectively). All comparisons were done using Mann–Whitney tests, with P-values of taxa associations corrected for each dataset-disease-scenario using Benjamini–Hochberg correction to obtain *Q*-values. We first determined proportions of each species group that were validated in these additional gut microbiome profiles across both Shotgun and 16S datasets and then investigated the group-wise affiliations of species that associated negatively (green) and positively (red) with Kendall Uniqueness (Fig. 2). The associations of ~ 70% of the multiple disease-enriched and disease-depleted (as identified in Ghosh et al^1^. were reproduced with the expected directionalities, with the former group overlapping significantly with the Kendall Uniq. +ve group and the latter with the Kendall Uniq. -ve (Supplementary Text S1). Please Table 1 Foot Note for disease abbreviations. Source data

We next checked if the membership of these groups (Fig. 2) showed differences in their association patterns with a putatively ‘beneficial microbiome’, with respect to the relative proportions of ‘putatively beneficial’ and ‘potentially detrimental’ taxa^8^. Here, we operationally defined a potentially detrimental taxon as being enriched in (or positively associated with) multiple diseases that is ‘disease associated’, whereas a putatively beneficial taxon was defined as one that is health associated or inversely correlated with multiple diseases (that is, ‘health associated’). We have previously identified 36 ‘multiple-disease-depleted’ and 23 ‘multiple-disease-enriched’ taxa that were enriched or depleted in multiple diseases, respectively^1^. Here, we considerably expand the study dataset with multiple cohorts and diseases (11,950 gut microbiomes from 22 cohorts) (Supplementary Table 10). Reinvestigating the disease associations of the above set of 59 health- and disease-associated taxa (originally identified in^1^) in the newly included datasets of the current study (that is, not considered in Ghosh et al.^1^), indicated a high reproducibility of the disease associations of these taxa in the additional metagenomic and 16S data (Methods, Supplementary Note 1 and Extended Data Fig. 6). Notably, although our previously identified set of health-associated taxa overlapped significantly with the Kendall uniqueness-negative group, the previous list of disease-associated taxa overlapped significantly with the Kendall uniqueness-positive group in the current study (Extended Data Fig. 6 and Supplementary Note 1).

The Kendall uniqueness positive group (Fig. 2) contained species including *Clostridium symbiosum*, *Clostridium ramosum*, *Ruminococcus gnavus*, *Clostridium hathewayi*, *Clostridium citroniae* and *Clostridium bolteae*, many of which we and others have identified as enriched in multiple diseases and associated with frailty in the ELDERMET cohort^1,14,18–20^. The Kendall uniqueness-negative taxa (Fig. 2) largely comprised species previously associated with health, including *Faecalibacterium prausnitzii*, multiple species of the *Coprococcus* and *Roseburia* genera, *Eubacterium rectale*, *Eubacterium eligens*, *Barnesiella intestinihominis* and *Odoribacter splanchnicus* (showing significant negative associations with Kendall uniqueness), all of which are depleted in multiple diseases^1,14,18^, as well as being associated with healthy aging trajectories^21^. Other members of this group included *Akkermansia muciniphila*, which although positively associated with Bray–Curtis and Jaccard uniqueness, showed negative associations with Kendall uniqueness. Thus, increasing uniqueness and diversity are features of an aging–host microbiome in general (especially for the Westernized populations), but not necessarily a signature of a putatively beneficial microbiome. Our previously defined lists of disease-associated and health-associated taxa overlap significantly with ab initio species-level groups defined here based on association with Kendall uniqueness. Thus, among the summary statistics tested, Kendall uniqueness is an efficient microbiome-summary measure to define the health correlation of a given microbiome or cohort based on constituent taxa. We next investigated the age-related abundance changes in these taxa especially in the gut microbiome of older adults in the diverse cohorts.

### Specific Kendall uniqueness-positive taxa increase with age in older adults

**Fig. 3:**
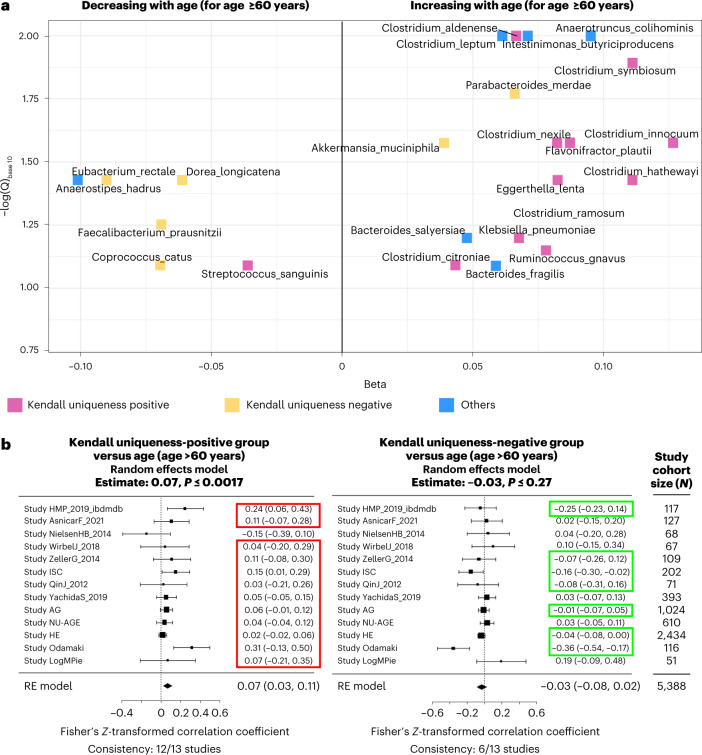
Increase with age in older adults of the disease-associated species that correlate positively with Kendall uniqueness. **a**, Volcano plot showing the association of the clr-transformed abundances of species in the two major species groups (identified in Fig. 2) with increasing age in the microbiomes of older adults (age ≥60 years). The *x* axis shows the summarized estimate of the random effects model-based association meta-analysis for each species determined across the 13 selected studies (Results), along with the study cohort size (the number of independent samples/gut metagenomes from each study). The *y* axis shows the −log(*Q*)_base 10_, where the *Q*-value is obtained by correcting the overall *P* values obtained for the same meta-analyses across all species using the Benjamini–Hochberg correction. Taxa belonging to the three taxon groups identified in Fig. 2 are shown in different colors (pink, Kendall uniqueness positive; yellow, Kendall uniqueness negative; blue, others). Only taxa showing associations with *Q* ≤ 0.1 are indicated. Taxa showing significant (*Q* ≤ 0.1) positive associations with age tend to be dominated by those belonging to the Kendall uniqueness-positive group. **b**,**c**, Overall increase of disease-associated group of taxa with increasing age >60 years; forest plots show the results of separate random effects (RE) model-based meta-analyses performed the group abundances of the Kendall uniqueness-positive and Kendall uniqueness-negative groups with age (>60 years) (Methods). The highlighted study cohorts (highlighted in green for health-associated Kendall uniqueness-negative group and in red for disease-associated Kendall uniqueness-positive group) are those where the association pattern was similar to the overall pattern. For each plot, the effect size of the associations with age is depicted as a line, with the mean effect size shown as black squares (the size proportional to the weight or power for each study), and the lines indicate the confidence interval of this estimate. The summarized effect size is indicated at the bottom in the shape of a rhomboid, with the outer edges indicating its confidence interval. The two-sided *P* values of the permutation tests of each random effects model is also indicated above each plot. Source data

We next focused on 13 studies with at least 50 gut microbiomes from people older than 60 years (Methods), allowing us to assemble 5,388 datasets from older persons. These 13 studies included 11,264 gut microbiomes from younger individuals (age <60 years). When we analyzed the age-association of the 54 health-associated and 22 disease-associated taxa identified in Fig. 2, 13 of the 22 Kendall uniqueness-positive taxa showed an increase with age over 60 years (in at least two-thirds of the cohorts). For 11 of these taxa, the increase was significant (overall random effects model *Q* ≤ 0.1) (Supplementary Table 11 and Fig. 3a). The taxa involved included *Clostridium symbiosum*, *Clostridium innocuum*, *Clostridium aldenense*, *Clostridium nexile*, *Flavonifractor plautii*, *Eggerthella lenta*, *Clostridium hathewayi*, *Clostridium ramosum*, *Klebsiella pneuomoniae*, *Ruminococcus gnavus* and *Clostridium citroniae*. As noted above, many of these taxa have been previously linked with frailty^1,17,22^. In contrast, the age relatedness of the health-associated species was variable and generally negative. Some of the major members of this group, namely, *Eubacterium rectale*, *Dorea longicatena*, *Faecalibacterium prausnitzii* and *Coprococcus catus*, significant decreased with age in older individuals (all with random effects model *Q* ≤ 0.1 and consistency ≥ 66.7%) (Fig. 3a and Supplementary Table 11). In contrast, other members of this group like *Akkermansia muciniphila*, previously linked to healthy aging^23^, showed an increase with age in older individuals.

There was a significant overall positive association between the abundance of the Kendall uniqueness-positive taxon group with age >60 years (random effects model estimate = 0.07, *P* = 0.0017), with the positive link replicating in 12 out of the 13 individual study cohorts (Methods and Fig. 3a). Although no significant pattern was observed with respect to the association of health-associated Kendall uniqueness-negative taxa with age (Fig. 3b), the rate of age-related increase in the Kendall uniqueness-positive taxa was much stronger than that of the health-associated species group (Mann–Whitney test of association coefficients, *P* = 5.3 × 10^−6^), whose variation with age trended toward a decrease (Supplementary Fig. 7a). This pattern was replicated even when we considered only the microbiomes from the apparently nondiseased control participants (Mann–Whitney test of association coefficients *P* = 2.3 × 10^−6^). Similarly, the significant age-associated increase in the grouped abundance of the Kendall uniqueness-positive species was also replicated when only considering the apparently nondiseased controls (random effects model estimate = 0.053; *P* = 0.004) (Supplementary Fig. 7b). Notably, the species belonging to the others group (showing no association with Kendall uniqueness) showed an association that was intermediate between that of health and disease-associated taxa, with no overall association with aging (Supplementary Fig. 7a,c).

We used random effects models to investigate the association of functional pathways with the abundances of health and disease-associated species groups (Supplementary Table 12), focusing on 41 pathways that were positively linked with the health-associated group and negatively linked with the disease-associated taxon group. This group included multiple pathways: synthesis of tryptophan and its precursor chorismate; biosynthesis of arginine, ornithine and other polyamines; and synthesis of multiple B vitamins, including folate (B9), pantothenate (B5) and thiamin (B1) (Supplementary Table 12). In addition to the positive associations of vitamins with health, multiple previous studies have shown the association of tryptophan, arginine and polyamine metabolism with improved cognitive function, improved colonic barrier function and reduced inflammation^24–26^. The 41 health-linked pathways included those for oxidation of fatty acids and elongation of unsaturated fatty acids that are linked to higher cognitive function^24^.

### Taxa are better markers of unhealthy aging than most summary indices

**Fig. 4:**
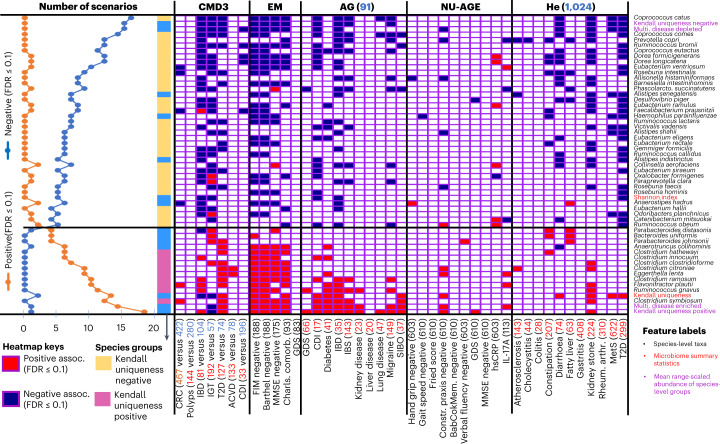
Ranked order of microbiome features that show the most consistent associations with multiple measures of unhealthy aging. The results are shown for 43 measures of unhealthy aging phenotype in five data repositories. Disease groups containing information from less than 20 gut microbiomes were not included in this analysis. Only those features that associate consistently with multiple measures of unhealthy phenotype individually in at least three of the five data repositories and at the maximum of only two association in the opposite direction are shown. The associations are shown for individual species, mean range-scaled abundances of the Kendall uniqueness-positive and negative groups (Fig. 2) and that of the multiple-disease-enriched and multiple-disease-depleted taxon groups identified in Ghosh et al. ^1^, along with multiple microbiome summary statistics used here (Methods). *Q*-values were obtained using Benjamini–Hochberg correction for each data repository-unhealthy measure combination. Features are arranged such that those showing the most negative associations with unhealthy older adult-specific scenario (at least with *Q* ≤ 0.1) are at the top, with a gradual shift to putatively detrimental features showing the most positive associations with negative health (at least with *Q* ≤ 0.1). The two groups differentially associating with unhealthy aging phenotypes are demarcated with horizontal lines. For each association, we have also indicated the number of gut microbiomes investigated. For CMD3 and ISC, containing samples from multiple studies, we used the matched patient-control studies pertaining to each disease (number of controls in blue font and patients in red font). For single AG and He cohorts, we compared taxon abundances in patients versus controls from the same data repository (size of each disease group indicated in red and the number of controls in blue besides the repository names). For EM and NU-AGE, all microbiomes were considered (number in parentheses), and associations were performed along a continuous gradient (Methods). Abbreviations: FIM, functional independence measure; Barthel, Barthel score; MMSE, Mini Mental State Examination; Charls. comorb., Charlson comorbidity; GDS, geriatric depression scale; hand grip, hand grip strength; Constr. praxis, sensitivity C-reactive protein; MetS, metabolic syndrome; Rheum. arthr., rheumatoid arthritis (Table 1 lists additional abbreviations). Source data

Five data repositories (CMD3, AG, ISC, specifically ELDERMET; NU-AGE and He) provided participant metadata indicative of normal/unhealthy aging status of the participants. Selecting adults >60 years of age resulted in 43 combinations of data-repository-versus-unhealthy-phenotype metadata (Methods and Fig. 4). We selected 116 microbiome features (107 species-level taxa, 4 measures of uniqueness, Shannon diversity, group abundances of the disease-associated taxa (showing positive association with Kendall uniqueness), the health-associated taxa (showing negative Kendall association) and the group abundances of our previously identified ‘putatively beneficial’ and ‘potentially detrimental’ taxa^8^, and we tested their association with the unhealthy aging metadata in each of the 43 combinations (Fig. 4 and Methods). We identified a set of 55 features that positively or negatively associated (with *Q*-value of 0.10 or lower) with multiple measures of unhealthy aging in at least three of five data repositories (allowing a maximum of two associations in the opposite direction across all repositories). These define an operational core set of healthy aging-associated microbiome markers, 16 of which were consistently negatively associated with healthy aging and 39 showing consistent positive associations with healthy aging (Fig. 4).

Of the features tested, the group abundance of the Kendall uniqueness-positive taxa (associated with disease) showed the most consistent positive associations with unhealthy aging, being positively associated (*Q* ≤ 0.1) with the highest number (18 of the 43) of the tested microbiome-unhealthy aging pairs for which such data were available (Fig. 4). Thus, the disease-associated species group showed not only a significant increase with aging in general but also the most consistent association with an unhealthy aging phenotype. This was followed, in rank order of association strength, by the abundance of multiple individual species belonging to this species group, like *Clostridium symbiosum*, *Ruminococcus gnavus*, *Flavonifractor plautii*, *Clostridium ramosum*, *Eggerthella lenta*, *Clostridium citroniae*, *Clostridium clostridioforme*, *Clostridium innocuum* and *Clostridium hathewayi*. In addition, the group abundance of the 36 disease-associated taxa (previously identified by us^1^) was also among the top features positively associated with the unhealthy aging (associated with 14 combinations). Among the microbiome summary statistics, as expected, only Kendall uniqueness was identified among the 16 top features associated with unhealthy aging (positively associated with 13 unhealthy aged phenotype scenarios).

*Coprococcus catus* and *Coprococcus comes* from the health-associated Kendall uniqueness-negative taxon group, along with the combined abundance of this group (as a whole), *Prevotella copri*, *Ruminococcus bromii* and that of the 23 disease-depleted group of taxa (previously identified^1^), were the top six microbiome features negatively associated with at least 12 clinical health/disease states (and with *Q* ≤ 0.1) (Fig. 4). This was followed in rank order by a multiple taxa from the health-associated taxa. There were relatively fewer associations between other measures of uniqueness or Shannon diversity and unhealthy aging with, for example, Shannon diversity negatively associated in 5 out of 43 scenarios with *Q* ≤ 0.1. Thus, except for Kendall uniqueness, none of the measures of microbiome uniqueness are a marker of healthy (or unhealthy) aging.

**Extended Data Fig. 7:**
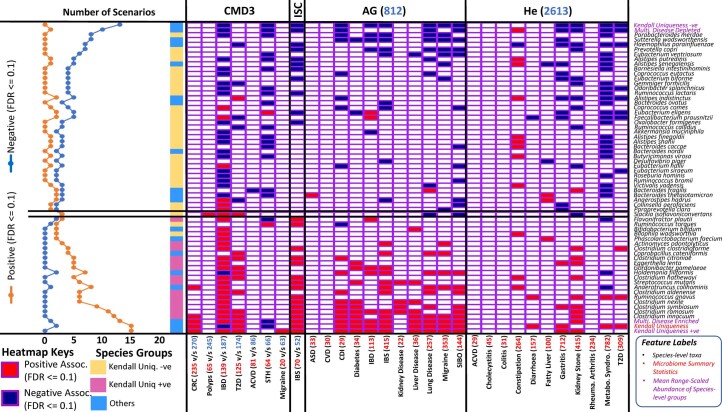
Identification of a ranked order of microbiome features that show the most consistent associations with multiple measures of unhealthy phenotype in younger people (age < 60 years). The results are shown for 30 measures of unhealthy phenotype in the younger participants (age < 60 y) from four data repositories. Disease groups containing information from less than 20 gut microbiomes were not included in this analysis. Only those features that associate consistently with multiple measures of unhealthy phenotype individually in at least two of the four data repositories and at the total maximum of only two association in the opposite direction are shown. The associations are shown for individual species, mean range-scaled abundances of the disease-associated and the health-associated groups identified in this study, the combined abundances of the multiple-disease-enriched and multiple-disease-depleted taxa groups previously identified in Ghosh *et al*.^1^, along with the different multiple microbiome summary statistics. P-values were FDR-corrected for each data repository-unhealthy measure combination to obtain the *Q*-values. Features are arranged such that those that show the most positive associations with negative health (at least with a *Q* < = 0.10) are shown at the top with a gradual shift to putatively beneficial features showing the most negative associations with negative health (at least with a *Q* < = 0.1). The number of gut microbiomes being compared for the association investigation in each scenario are indicated. The convention adopted is as described in the legend of Fig. 5. Abbreviations for clinical measures/disease phenotypes: CRC: Colorectal Cancer, IBD: Inflammatory Bowel Disease, IGT: Impaired Glucose Tolerance, T2D: Type II Diabetes, ACVD: Atherosclerotic Cardiovascular Disease, CDI: Clostridioides difficile infection, STH: Soil Transmitted Helminths, ASD: Autism Spectrum Disorder, CVD: Cardiovascular Disease, SIBO: Small Intestional Bacterial Overgrowth; Metabo. Syndro.: Metabolic Syndrome, Rheuma. Arthritis: Rheumatoid Arthritis. Source data

Younger individuals (Methods and Extended Data Fig. 7) were notably different in the identity and ranking of microbiome taxa associated with health loss (only 40 of 64 features overlapped), with younger-individual microbiomes having a distinct abundance of Bacteroides, *Parabacteroides* and *Alistipes* taxa associated negatively with the unhealthy phenotype. However, as for the older adults, the strongest positive and negative associations were with disease-associated Kendall uniqueness-positive and the health-associated Kendall uniqueness-negative taxa groups, respectively.

### Healthy aging markers occupy core positions in the gut microbiome

Higher Kendall uniqueness is a direct reflection of a change in internal microbiome hierarchy, with a loss (or depletion) of numerically dominant microbiome members and higher abundance of subdominant taxa. Distinct interactions of the health-associated (Kendall uniqueness-negative) and disease-associated (Kendall uniqueness-positive) taxon groups with measures of unhealthy aging likely reflect distinct functional roles and positions in microbiome ecological networks. Older age and increased duration in residential care covary with loss of diversity-associated taxa and core taxonomic modules in ELDERMET participants^4,5^. In the NU-AGE study, taxa associated with healthy aging and whose abundance increased with MedDiet adherence were enriched in the microbiome core and occupied highly connected nodes in the microbiome network^15^. These observations show that the retention of the core is associated with healthy aging. Here, we validated and extended these concepts by coabundance network analysis across the 12 datasets from the seven data repositories (the 13 datasets from Fig. 3, omitting NU-AGE, which lacked microbiomes from younger individuals; Methods). This constituted 4,778 microbiomes from older adults (age ≤60 years) and 11,264 microbiomes from younger individuals. Comparing the centrality measures of the taxa indicated an overall similarity between the coabundance networks obtained for the older and younger individuals (Supplementary Table 13). Only *Prevotella copri* and *Dorea longicatena* displayed significantly higher centrality in the older-individual-specific networks.

**Extended Data Fig. 8:**
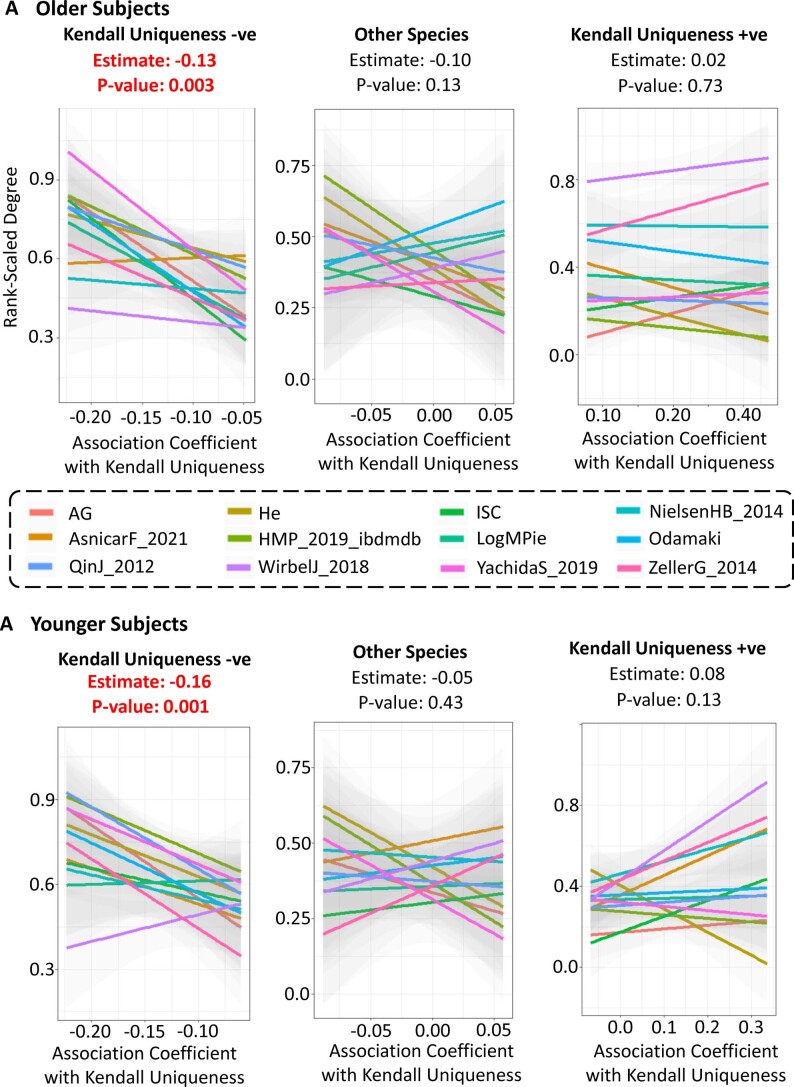
Relationship between degree centrality and the strength of the association with Kendall Uniqueness for the different species-level taxon groups. Least-square regression lines linking degree centrality measures of the different taxa and their extent of association (association coefficient) with Kendall Uniqueness. Regression-lines are shown separately for taxa belonging to each of the three different species-level-groups. For each species-group, distinct regression lines are shown separately for the co-occurrence networks obtained for each study. Panel A shows these relationships only in the study-specific co-occurrence networks derived for the gut microbiomes from older participants (participant age > = 60 years); Panel B shows the same for networks derived for younger gut microbiomes (participant age < 60 years). For both the plots, bold lines indicate the mean regression line for each of the associations, the shaded regions (in gray) corresponding to each line indicate their confidence intervals (+/- standard errors). The p-values indicated above each plot are obtained using Random Effect Models (utilizing two-sided permutation tests). Source data

There were some largely consistent features of the centrality measures for the different Kendall uniqueness-defined taxon groups across the different studies (irrespective of age). The health-associated group of taxa occupied core positions in coabundance networks, shown by significantly higher centrality measures of degree (older individuals: random effects model *P* value = 0.032, consistency: 75% of studies, Supplementary Fig. 8; young: random effects model *P* value = 0.0001, consistency: 92% of studies, Supplementary Fig. 9), betweenness (older adults: random effects model *P* value = 0.0013, consistency: 83% of studies, Supplementary Fig. 10; young: random effects model *P* value = 0.0001, consistency: 92% of studies, Supplementary Fig. 11) and hub score (older adults: random effects model *P* value = 0.027, consistency: 67% of studies, Supplementary Fig. 12; young: random effects model *P* value = 0.0003, consistency: 83% of studies, Supplementary Fig. 13) of this taxon group compared with those for the disease-associated Kendall uniqueness-positive group (Supplementary Figs. 8–13). The Kendall uniqueness-negative taxon group also had significantly higher prevalence than the Kendall uniqueness-positive group (Supplementary Fig. 14). Higher connectivity and higher prevalence indicated that the health-associated taxa are part of the core microbiome. Notably, within this group, the more central a taxon was in the coabundance network, the stronger was its negative association with Kendall uniqueness (Extended Data Fig. 8) across the coabundance networks obtained for both the older (random effects model estimate: −0.13, *P* value = 0.003; pattern observed consistently in 11 out of 12 study cohorts) and younger individuals (random effects model estimate: −0.16, *P* value = 0.001; pattern observed with consistency in 10 out of 12 study cohorts). Thus, increasing Kendall uniqueness is directly linked with a loss of the core microbiome structure, which in turn is associated with an unhealthy phenotype in both the young and older participants and is also a microbiome feature of aging in general.

**Fig. 5:**
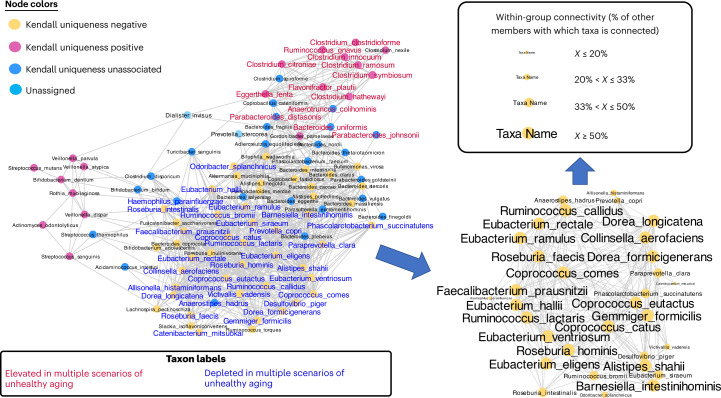
Identification of a coabundant hub of putatively beneficial symbionts that are depleted in unhealthy aging. Coabundance network of taxa derived from microbiomes of adults older than 60 years, across 13 individual studies. We selected a set of 112 species that were identified in both 16S and Shotgun datasets. Associations between the centered-log-ratio transformed abundances of species pair were individually computed within each study using robust linear regression models. Results of the individual robust linear regression models were then collated using random effect models to compute summarized association statistics. For each species, the summarized association *P* values for every other were then corrected using the stringent Bonferroni approach and only those species pairs having a *Q* ≤ 0.001 and an overall summarized positive association estimate (>0) were determined to have coabundant relationships and connected by an edge. The species-level nodes belonging to the different species groups are filled in different colors, namely, green for the health-associated group, red for the disease-associated group and light blue for other species. Species-level taxa that are observed to be either elevated or depleted in multiple scenarios of unhealthy aging are shown in brown and dark blue, respectively. We also investigated the interactions for the taxa depleted in multiple scenarios of unhealthy aging (Fig. 5) individually within the 11 studies (Results). This species-to-species coabundance subnetwork of health-associated markers is shown in the bottom right corner. The sizes of the labels are based on the number of connections each taxon has with the others in this subnetwork. Source data

**Extended Data Fig. 9:**
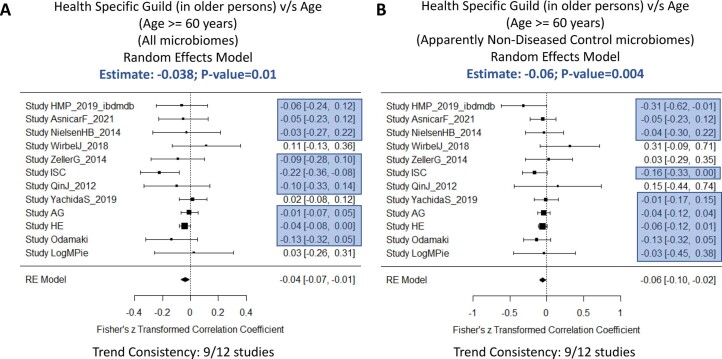
Single consensus coabundance network of taxa combining all the microbiomes from younger participants with age < 60 years from the 12 individual studies. We selected a set of 112 species that are commonly identified in both 16S and Shotgun datasets. Associations between the centered-log-ratio transformed abundances of species pair were individually computed within each study using robust linear regression models. Results of the individual robust linear regression models were then collated using Random Effect models to compute summarized association statistics. For each species, the summarized association p-values for every other were then FDR-corrected and those species having a stringent threshold of *Q* < = 0.001 and an overall summarized association estimate of greater than 0 were determined to have co-abundant relationship with it. The species-level nodes belonging to the different species groups are filled in different colors, namely green for Kendall Uniqueness negative, red for Kendall Uniqueness positive and light blue for other species. Species-level taxa that were either elevated or depleted in multiple scenarios of unhealthy young are shown in deep pink and dark blue, respectively. Source data

**Extended Data Fig. 10:**
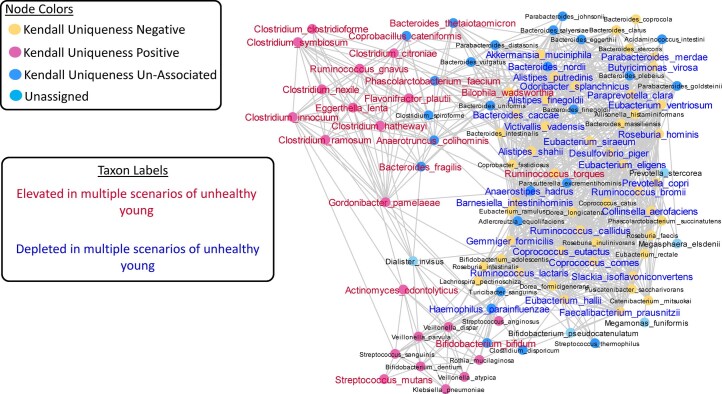
Variation of the healthy aging associated taxonomic guild with age in older people (age > = 60 years). Forest plots showing the results of the Random Effects Models investigating the variation of the mean ranked abundance of health-associated taxonomic guild of 12 species (in older persons) across age in the cohorts considering: A) All the microbiomes B) Only the microbiomes from apparently healthy nondiseased controls. For each forest plot, the effect size of the associations of different taxon groups with age is depicted as a line with the mean effect size shown as black squares (the size of the squares proportional to the weight or power for each study) and the length and the end-points of the line indicate the confidence intervals of this estimate. The summarized effect size is indicated at the bottom in the shape of a rhomboid, the outer edges of which indicates its confidence interval. The number of samples (or gut microbiome) (n) corresponding to the different studies are: AG:1023, AsnicarF_2021:127, HE:2434, HMP_2019_ibdmdb:117, ISC:202, LogMPie:51, NielsenHB_2014:68, NU-AGE:610, Odamaki:116, QinJ_2012:71, WirbelJ_2018:67, YachidaS_2019:393, ZellerG_2014:109. Two-sided P-values for the Random Effect Model were computed using permutation tests of association for the summary effect sizes. Source data

To investigate the relative placement of microbial markers of the unhealthy phenotype in the coabundance networks, we generated consensus coabundance networks based on the consistent abundance associations between species pairs observed across the 12 individual studies (separate networks constructed for young and older participants) (Methods, Fig. 5 and Extended Data Fig. 9). The consensus networks for older and younger individuals both consisted of a large densely connected core hub of most of the health-associated Kendall uniqueness-negative taxa and two subhubs of the disease-associated taxa. One of these subhubs of disease-associated Kendall uniqueness-positive taxa comprised multiple species from the *Streptococcus* and *Veillonella* genera and other species like *Klebsiella pneumoniae* and *Actinomyces odontolyticus*, whereas the other subhub contained multiple disease-associated *Clostridium* species, along with *Ruminococcus gnavus*, *Flavonifractor plautii* and *Eggerthella lenta*. The taxa that were not associated with Kendall uniqueness were located either in the periphery or acted as linking hubs between the health-associated core and the two disease-associated subhubs. The distinguishing feature between the two consensus coabundance networks (for the two age groups) was the placement (as shown in Fig. 5 and Extended Data Fig. 9) of the taxa that are elevated/depleted in multiple cases of unhealthy phenotype (in the corresponding age groups). In the younger-participant consensus network, the taxa depleted in multiple examples of health loss were spread across the core hub of the coabundance network. Similarly, those elevated in multiple scenarios of health loss were also distributed across the two disease-associated subhubs (Extended Data Fig. 9). In contrast, the positive and negative markers of unhealthy aging in older adults were localized to specific subregions in the corresponding consensus coabundance network for the older participants (Fig. 5). Eleven taxa whose abundance was elevated in multiple scenarios of aging (in Fig. 4) were present in one single disease-associated taxa-dominated subhub of this network (Fig. 5), whereas 39 taxa depleted in multiple scenarios of unhealthy aging (as in Fig. 4) were colocalized to a specific region of the core hub of this network. A total of 19 of these 39 taxa exhibited a dense network of coabundance relationships amongst themselves (wherein each member of this 19 taxa group had coabundance relationships with at least 50% of the other 18 members), thus forming a coabundant guild of 19 taxa putatively associated with healthy aging (Fig. 5 and Supplementary Table 14). Notably, this 19-species older-specific health-associated guild showed reduced abundance with age beyond 60 years of age across at least 75% of the studied cohorts (random effects model *P* value = 0.019 taking all microbiomes; random effects model *P* value = 0.004 only considering the apparently nondiseased controls, trend replicated across 75% of the study cohorts) (Extended Data Fig. 10).

Age-related health loss differs between people, despite apparently starting from similar health status at younger age. We therefore examined the rate of loss or gain with age of four microbiome parameters in the four cohorts that included either control and disease groups (CMD, AG and He) or healthy and unhealthy aging groups (EM) (Supplementary Fig. 15). Unhealthy aging was characterized by either faster age-associated loss of taxa whose abundance may be the key for healthy aging or consistently lower levels of these taxa with respect to the controls group. In the He and CMD cohorts, the nondiseased participants showed a higher rate of increase of uniqueness with age (consistent with previous observations)^6^, but this is not an indicator of healthy aging, because individuals in the disease group displayed significantly higher uniqueness from a much younger age.

## Discussion

This study explored whether determining the gain or loss of specific taxa represent a more precise metric of healthy/unhealthy aging than summary microbiome statistics, such as diversity and uniqueness. We assessed the interaction between specific microbiome taxa and summary statistics with aging and health in a heterogeneous global dataset derived from 19 different nationalities spanning five different continental regions. The study identifies that the gut microbiome alterations associated with both aging in general and unhealthy aging are characterized by a common theme: loss of the core microbiome structure (specifically a coabundant species-level guild of the core microbiome) and concomitant increase of a specific guild of disease-associated taxa.

To address the confounding effects between incidence of specific diseases and aging in general, we have investigated the above patterns using a two-step investigation strategy. We first investigated all gut microbiomes from individuals aged >60 years and then revalidated our findings within the gut microbiomes from the apparently nondiseased controls. However, it is important to note that biological aging in general may be accompanied by increased incidence of conditions like dyslipidemia, hypertension and inflammation, which might not have been specifically recorded in all reports but are linked with the microbiome composition^27^. Another limitation of the datasets available for this study was the underrepresentation of extreme older adults (for example, centenarians), with the majority of data being from individuals younger than 100 years of age. This impeded our investigating healthy versus unhealthy aging trajectories in individuals in the extreme age ranges. Previous studies have attempted to profile the gut microbiome of centenarians in general (reviewed previously^7^) and link the features of a centenarians’ gut microbiome with healthy aging. However, it is important to note that, increased life-span is not equivalent to health span. Although all centenarians clearly exemplify healthy aging trajectory in their past lives, their current physiological statuses will show individual-specific health differences that need to be stratified before performing microbiome-aging association studies for these individuals.

For future aging-microbiome studies, an alternative universal approach to address the above confounding effects would be to use the ‘biological age’ or the ‘rate of aging’ rather than the chronological age of the individuals for these investigations. A multitude of omics-based aging clocks are currently available and can predict not only an overall biological age or ‘accelerated rate of aging’ in an individual (irrespective of the chronological age) but also the age-related decline with respect to specific attributes of health^28^. Despite the above limitations, the identification of specific guilds of bacteria could be used for designing older-people-targeted microbiome-based therapeutic interventions and as diagnostic markers of individuals (middle-aged or at the onset of aging) who are at risk for an unhealthy aging trajectory.

Differences in the baseline composition of an apparently healthy gut microbiome within a given study population could influence the strength and consistency of the above alterations depending upon the nature of the study population. For example, the strongest effects of the aging-associated gut microbiome changes were detected for the European and North American individuals. Notably, a majority of members of the bacterial guild associated negatively with unhealthy aging were reported in a previous study by our group to be more abundant in nonindustrialized populations, as well as in the Irish Travellers living a more traditional lifestyle compared to settled industrialized societies^29^. Could the specific markers of health in older people identified in the current study have a reduced rate of loss in these populations, resulting in the retention of a resilient microbiome into late aging? It would be desirable for future studies to include a greater representation of older adults from nonindustrialized countries to further examine the weaker diversity and uniqueness associations with age and health noted for those geographies in the current study.

To address the issue of baseline differences across study populations, we identified that the Kendall uniqueness measure efficiently captures the relative loss of the core microbiome and microbiome organization in an individual with respect to a given reference population. In essence, the concept of Kendall uniqueness further resonates with the previously proposed ‘Anna-Karenina principle’ of the microbiome that ‘All happy microbiomes look alike, each unhappy microbiome is unhappy in its own way’^30^. We have previously shown in the ELDERMET cohort that increased duration of illness-associated hospitalization is associated with a loss of the core microbiome^4,5^. Similarly, the microbiome of people with conditions like inflammatory bowel diseases and colorectal cancer also display loss of specific core taxonomic groups (identified in the current study) and increased variability in the gut microbiome^31–33^. Additionally, higher abundance of particular core microbiota taxa has been shown to facilitate faster recovery of the microbiome following antibiotic treatment^34^. The identification of the Kendall uniqueness metric in the current study indicates that the retention of the microbiome core and hierarchical abundance in the microbiome could be the key driver facilitating microbiome resilience and homeostasis. The identification of such a microbiome summary index that efficiently captures the state of the microbiome with respect to the corresponding reference population will have translational value.

Equally importantly, we also identify specific groups of taxa that are associated (either positively or negatively) with Kendall uniqueness. These specific taxon guilds show consistently stronger associations with the unhealthy aging phenotype than the Kendall uniqueness measure itself. Thus, although the latter could serve as a population-level microbiome summary statistic to capture the state of microbiome (decline) in an individual, the taxa defined here are expected to have diagnostic and therapeutic value.

There is also a need for further studies that investigate the microbiome at higher resolution. Strain-level resolution offers a more crystalline view of microbiome-disease associations. Gene presence/absence analyses or a single-nucleotide polymorphism-level meta-analysis of gut microbiomes from multiple geographic locations will also be informative but will require uniformly high-quality metagenomic data across all cohorts/participants, plus detailed metadata.

## Conclusion

The definition of a healthy microbiome is dependent on context. However, age-related changes in the microbiome are identifiable and more reliably linked with health and disease than in youth. Many of the health and disease associations of particular taxa were previously validated by the NU-AGE MedDiet intervention study^15^, which demonstrated healthier aging in the dietary intervention group, which tended to retain putatively beneficial symbionts. However single time-point measures of gut microbiome diversity or uniqueness will not provide actionable information. Rather, the proportions of disease or health-associated taxa are likely to be a superior therapeutic target and metric of clinical status and benefit.

## Methods

### Statement on ethical regulations

The study used meta-analysis on publicly available deanonymized data and did not collect data from human participants as part of this study. The details on protocols involving different aspects of the human study participants (sex, number and age of participants and statements on informed consent), including relevant ethical regulations, name of the board/committee and institution that approved the study protocol, are described in the original studies (which have been referred to in this study).

### Statistics and reproducibility

Because the current study is a meta-analysis of several publicly available datasets, no statistical method was used to predetermine sample size in this study. We have attempted to include all data from each of the available datasets. Wherever applicable, we have described the criteria used to select the specific subsets of studies. Similarly, the methods pertaining to the mode of collection of data from individuals (for example, whether performed blind or not) can be obtained in the publications corresponding to the individual studies. For many parts of our analysis, we have relied on nonparametric tests, whereas for others like the meta-analysis models, the data distribution was assumed to be normal, but this was not formally tested.

### Collation of gut microbiome data repositories

Table 1 provides the details of the seven data repositories included in this study. We used a total of 21,041 gut microbiome profiles (8,430 Shotgun sequenced and 12,611 16S amplicon based). The details of these five repositories are provided in Supplementary Note 2 (refs. ^1,10–16,35–39^).

To summarize, the seven data repositories included more than 21,000 samples, with similar representation of gut microbiomes profiled using both Shotgun and 16S rRNA gene amplicon-based approaches. Six of the data repositories contained samples from different nationalities across age landscape ranging from 18 to >100 years, and one cohort (NU-AGE) was older-adult specific. The seven repositories encompassed gut microbiomes from individuals residing in more than 20 different nationalities from Europe, North/South America, Africa and Asia. Of these, more than 6,400 microbiomes were especially from older individuals with age older than 60 years. For the older subset, four of the data repositories (except for Odamaki) also contained information with respect to 50 different clinical measures of unhealthy aging.

### Computation of genus-level, species-level and pathway-level abundances

The CMD3 and ISC data repositories were Shotgun based. For samples belonging to these two repositories, the species-level and genus-level taxonomic profiles were obtained using metaphlan2 (ref. ^40^). For the ISC datasets, the pathway-level abundances were obtained using the humann2 pipeline^41^; for CMD3, this information was already available in the repository and was directly used. The AG, NU-AGE, Odamaki, He and the LogMPie cohort datasets were 16S based. For these cohorts, for uniformity of taxonomic assignments across studies (or data repositories) and across taxonomic levels, we used the single SPINGO classifier pipeline for profiling the taxonomy at both the genus and species levels^42^. Given the compositional nature of the taxonomic and functional profiles, all data were converted to both relative abundances as well as transformed to the centered-log-ratio (clr) transformation for all subsequent steps of the investigation^43^ as described in Supplementary Note 3.

Many previous studies performing such across-studies meta-analyses have focused on relative abundance data for identifying disease-specific and shared markers of multiple diseases^1,18,44^. However, as described previously, given the compositional nature of the microbiome datasets, clr transformation has been strongly suggested as the ideal normalization measure for performing such investigations^45^ and was used for a majority of species abundance associations with different microbiome properties and age. Thus, to relate the results of the current study with the previously published studies (on microbiome markers of health and disease) while at the same time accounting for the compositionality of the datasets, it was important to investigate the effects and relationships among the different normalization measures utilized here in (relative abundance and clr transformation) before performing this association analysis. Across the 28 studies, we observed a strong positive correlation between the total-sum-scaled relative abundances and the clr-transformed abundances of the constituent microbiome taxa at both the species and genus level (Supplementary Figs. 4 and 5). The correlations were computed using corr.test function of the psych R package (version 2.1.9)

### Computation of microbiome summary indices

In this study, we profiled five different microbiome summary indices, namely, Shannon diversity and the four different measures of uniqueness. The computation of these summary indices is described in Supplementary Note 4 (ref. ^6,46^). All summary indices were computed separately for samples constituting each individual study.

Each of the different uniqueness measures computed using a different distance scheme, captures different aspects of variations within gut microbiomes, including variations in the detection, abundance (considering relative abundances as well as compositionality of the microbiome data) and the overall hierarchical ordering within the gut microbiome (Extended Data Fig. 1 and Supplementary Note 5)^47^. The alpha-diversity-corrected values of the different uniqueness measures were computed as the residuals of the regression models computed between the alpha diversity (available as the Shannon diversity) and the values of the corresponding uniqueness measures. For this purpose, we first utilized robust linear regression models (function rlm of the MASS package version 7.3.54) to regress (or to predict) each of the different uniqueness measures with Shannon diversity as the predictor in individually in each of the study cohorts listed in Table 1. The robust linear regression models are alternatives to simple linear regression models but are more robust to outliers^48^. This effect is achieved by assigning weights to each observation, penalizing outliers. The statistical significance of the fits or associations were computed using the two-sided robust *F*-test (performed using the f.robftest function of the sfsmisc package v 1.1.12 in R). Given a uniqueness measure and the microbiomes belonging to a study cohort, the alpha-diversity-corrected values for the uniqueness measure were then computed as the residuals from the robust linear regression models corresponding to that uniqueness measure in that study cohort.

### Two-step meta-analytic framework to investigate associations between microbiome properties and between microbiome features and age

We adopted a universal two-step meta-analytic framework to investigate the relationships within different microbiome summary indices and between different microbiome summary indices and age, between different microbiome summary indices and the microbiome features (at the level of species or pathways) and feature groups (species-level groups), among the different species-level features and between different species-level features and age (Supplementary Fig. 1). This framework is described in detail in Supplementary Note 6.

Associations of the overall beta diversity (that is, the variation in the overall compositions across the different microbiome) with age were computed individually within the study cohorts using the permutational multivariate analysis of variance (PERMANOVA) approach^49^. The PERMANOVA approach is dependent upon the measure utilized for profiling the differences across microbiomes (and thus on the overall distance matrix utilized). Thus, given that we profiled the differences across the different microbiomes using four different distance measures, each depicting related but nonidentical aspects of gut microbiome variations, we performed the PERMANOVA investigations of gut microbiome variation with age individually in each of the studies using each of the four distance measures. The adonis function of the vegan package version 2.5.7 was used for this purpose.

For each association investigation analyses, the sample (or microbiome) (or *n*) numbers for the individual considered studies provided in Table 1 and Supplementary Table 10. For random effect models, the ‘*n*’ numbers are also indicated in the corresponding forest plots provided in specific figures.

### Identification of taxa showing consistent positive and negative associations with different uniqueness measures and Shannon diversity

We investigated this first in a repository-specific manner, using an approach previously described (and summarized in Supplementary Fig. 1; 28 studies with study population numbers provided in Table 1). As described in Results, we specifically identified 107 species-level taxa that were commonly detected in at least 5% of the samples in at least 60% studies individually in both the Shotgun-based and 16S rRNA gene-amplicon-based data repositories, individually. Within gut microbiome samples belonging to a given data repository, association estimates and significance were obtained using robust linear regression models between the clr-transformed abundances of various taxa with each of the uniqueness measures and diversity (using the same strategy as described in the previous section). Each individual study as described in Table 1 (with investigation type as I) was investigated separately. These studies included the multiple studies within CMD (each with varying experimental methodologies for DNA sequencing and extraction). Within the individual studies, the *P* values of associations obtained using robust linear regression models were corrected separately for each of the five microbiome summary statistics (four measures of uniqueness and Shannon diversity), using the Benjamini–Hochberg correction to obtain the false discovery rate (FDR) (or *Q*-value) (computed using the p.adjust function with ‘method’ parameter = ‘fdr’ of the base R package version 4.1.0). The summarized associations of the abundances of the different species-level taxa with the different the different summary statistics were then investigated using the meta-analytic random effects models (using the previously described strategy). For the individual studies, for the different taxa, the *P* values of associations obtained using the random effects models were corrected separately for each microbiome summary statistics using Benjamini–Hochberg correction. The number of taxa showing significant positive or negative correlations with at least one of the uniqueness measures or diversity (FDR correcting for random effect Model z-test *P* value for each uniqueness/diversity measure < 0.05) and with a consistency ≥67% (proportion of individual cohorts where the directionality of associations obtained using robust linear regression models were the same as the summarized estimate obtained using the random effects model) were then identified. The total number of such associations obtained for each microbiome summary statistics were then summarized. The species-level taxa were then divided into three groups based on their association with Kendall uniqueness, namely negatively associated with Kendall uniqueness, positively associated with Kendall uniqueness and others.

### Computation of grouped abundances of different species-level taxa groups

The grouped abundances for each group (the three Kendall-linked taxonomic groups and the groups of multiple-disease-enriched and multiple-disease-depleted taxa previously identified in Ghosh et al.^1^) of species-level taxa were obtained as described below. For each taxa belonging to a group, the clr-transformed abundances across each sample (or microbiome) was first range-scaled as below:

$$\begin{array}{lll}{\mathrm{species - taxa - abundance}}_{\mathrm{range - scaled}}^{\mathrm\it{xj}} \\ = \left[ {{\mathrm{species - taxa - abundance}}^{\mathrm\it{xj}} }\right. \\ \left.{-{\mathrm{min}}\left( {{\mathrm{species - taxa - abundance}}^{\mathrm\it{j}}} \right)} \right]\\ {\mathrm{/}}\left[ {{\mathrm{max}}\left( {{\mathrm{species - taxa - abundance}}^{\mathrm\it{j}}} \right)}\right. \\ \left.{-{\mathrm{min}}\left( {{\mathrm{species - taxa - abundance}}^{\mathrm\it{j}}} \right)} \right]\end{array}$$

where species-taxa-abundance^*xj*^ is the abundance of species-taxa ‘*j*’ in sample ‘*x*’; min (species-taxa-abundance^*j*^) is the minimum abundance of species-taxa ‘*j*’ across all samples and max (species-taxa-abundance^*j*^) is the maximum abundance of species-taxa ‘*j*’ across all samples.

Subsequently, the grouped abundance of a species-level group was then obtained as the mean of the range-scaled abundances of all species-level taxa belonging to that group.

These included the three species-level groups identified based on their association with Kendall uniqueness (as described above), as well as the multiple- disease enriched and multiple disease depleted identified in Ghosh et al.^1^. The later groups were identified as below.

A previous analysis by our group on more than 2,500 gut microbiome samples covering five major diseases had previously identified distinct groups of species-level taxa that were observed to be either enriched or depleted in multiple disease. We had referred to this as G1-3 or taxa groups enriched across multiple diseases (we refer here as ‘multiple disease enriched’ or ‘disease associated’) and L1-3 or taxa groups depleted in multiple diseases (or ‘multiple disease depleted’ or ‘health associated’). For each gut microbiome (sample) in a given repository, the taxa belonging to either of the two groups were identified and their group abundances were calculated as described above.

### Replication of the disease association pattern of the multiple-disease-enriched and multiple-disease-depleted taxa in the additional cohorts considered in the current study

Our previous list of multiple-disease-enriched and multiple-disease-depleted taxa were obtained by investigating a five different diseases across eight study cohorts^1^. The current study however was considerably expanded (as summarized in Supplementary Table 10). Given that these cohorts were derived from different geographically placed populations covering additional disease scenarios, it was important to replicate the disease association of these multiple-disease-enriched and multiple-disease-depleted taxa on these cohorts. For this purpose, we compared the relative abundances of the different taxa constituting the two groups (between the diseased and control gut microbiomes) in these specific additional cohorts using two-sided Mann–Whitney tests. Before this, we showed that the both relative abundances and clr-transformed abundances generated nonidentical but significantly correlated values, indicating that in specific scenarios. For CMD3 and ISC data repositories, for each disease, we considered the patient gut microbiomes in the different study cohorts corresponding to that disease and compared the abundances of the species-level taxa belonging to the two groups with gut microbiomes from the matched controls belonging to the same study cohorts. For AG and He cohorts, the patient gut microbiomes for the different diseases and the gut microbiomes from the controls were sequenced as part of the same study. Thus, for each disease, the abundance of the different taxa in the gut microbiomes of the corresponding patients were compared with the gut microbiomes from all individuals that did not belong to any of the disease sub-cohorts. The direction of change as well as the *P* values obtained for each taxa were then obtained. For each combination of disease and study cohort (as depicted by the rows of the heatmaps shown in Extended Data Fig. 6), the *P* values obtained for each of the taxa belonging to the two groups were corrected using the Benjamini–Hochberg approach (as described above) to obtain the FDRs. Taxa observed to be enriched or depleted either FDR ≤ 0.1 were identified. A marker taxon was considered replicated if it satisfied either one of the following two criteria: (1) it associated with the expected directionality (positive for disease enriched and negative for disease depleted) in greater than two scenarios and in the opposite directionality at a maximum of two scenarios, or (2) it associated with expected directionality in less than or equal to two scenarios but never with the opposite directionality in any of the investigated scenarios.

### Association of gut microbiome taxa with age

The objective here was to investigate the variations of specific gut microbiome members (individual taxa as well as the grouped abundances of species-level taxa groups identified in Fig. 2) specifically with the onset and progression of aging, and not to explore the dynamics of these taxa in the younger or middle-aged individuals. Thus, in this investigation, we specifically focused on the trajectory starting from the onset of aging (age = 60 years). Thus, for this purpose, we subsequently focused on a group of 13 studies that contained at least 50 gut microbiomes from older individuals (age ≥60 years). The studies considered were HMP_2019_ibdmdb, AsnicarF_2021, NielsenHB_2014, WirbelJ_2018, ZellerG_2014, ISC, QinJ_2012, YachidaS_2019, AG, NU-AGE, He, Odamaki and LogMPie. This totaled to around 5,388 gut microbiome profiles from older adults (age ≥60 years), which were considered for this analysis. Subsequently, we adopted a similar approach as depicted in Supplementary Fig. 1, whereby we first investigated the associations of the of the different taxa with age ≥60 years within each individual study (using robust linear regression models) and subsequently overall using the meta-analytic random effects models. We performed this analysis in a two-step manner. In the first phase, we retained only those taxa that showed a consistent pattern of association (either positive or negative) with age post 60 years in at least two-thirds (67%) of the studies. Subsequently, only this set of filtered taxa showing reasonably consistent across-studies directionalities of association were then further investigated for statistical significance using the random effects model-based meta-analytic framework. The same strategy was used even while considering microbiomes from only the nondiseased controls across the studies.

### Association analysis between microbiome properties and indices of unhealthy aging across various data repositories

#### Study-based stratification

We used gut microbiome profiles from five different data repositories for this purpose, with datasets like CMD3 further containing profiles from multiple studies. Each study/data repository had considerable variations with respect to not only the microbiome profiling methodologies but also the geography of the study population and methods used for obtaining metagenomic sequence data. To address this variation and identify consistent signatures/associations, we repeated all our analysis individually for each measure of unhealthy aging within each data repository (as described below).

#### Feature association with the different unhealthy aging measures

With the exception of Odamaki and LogMPie, each of the other five data repositories had various clinical measures pertaining to the health status of the individuals (as described above). Across the five data repositories (CMD3, ISC or EM, AG, He and NU-AGE), there were a total of 43 measures of unhealthy aging, wherein each scenario contained information from at least 20 gut microbiomes. These various measures included the disease information, measures of physical frailty, inflammation, and cognitive impairment and decline and are shown in Fig. 4. For the continuous measures, associations of the various microbiome features with each of these measures was performed using robust linear regression models as below (as described previously):

$${\mathrm {fit}} = {\mathrm{rlm}}\left( {\mathrm {microbiome}\;{\mathrm {property}}\sim {\mathrm {clinical}}\;{\mathrm{measure}}} \right).$$

For the categorical measures (like disease presence/absence), the associations were performed investigated using Mann–Whitney tests as described previously for replication of the disease association pattern of the multiple-disease-positive (disease-associated) and multiple-disease-negative (health-associated) taxa.

The clinical measures were transformed such that each measure correlated positively with unhealthy aging phenotype. For example, disease information was transformed such that disease occurrence was assigned the value 1 and control status a value of 0. Indices that are expected to correlate positively with unhealthy aging like Fried score (positive index of frailty, higher values indicate more frailty), inflammatory marker levels (higher values indicate higher inflammation), geriatric depression scales (higher values indicate impaired cognitive/mental status) and Charlson comorbidity scores (higher values indicate greater comorbidity) were not transformed. However, indices that negatively associate with the unhealthy aging phenotype like FIM, Barthel score, hand grip strength, gait speed (higher values indicate lower frailty), MMSE, constructional praxis, verbal fluency score and Babcock memory (higher values indicate reduced cognitive impairment) were converted to their inverses (or negatives) by multiplying by −1 (refer to Supplementary Note 2 for abbreviations).

#### Identification of a ranked ordered of microbiome features

For this purpose, we combined the values pertaining to all the 116 investigated microbiome features (Shannon diversity, four measures of uniqueness, combined grouped abundances of the Kendall uniqueness-positive and Kendall uniqueness-negative species-level-taxa groups, combined grouped abundances of the multiple-disease-enriched (or disease associated) and multiple-disease-depleted (or disease-depleted) taxa^1^, the clr-transformed abundances of the 107 species-level taxa identified as described previously in Fig. 2). These measures are listed in the columns of the heatmap depicted in Fig. 4. We subsequently investigated the association of the 116 features with each of the 43 measures (or scenarios of unhealthy aging) as described below. Specific microbiome features showing multiple associations with the same directionality (to ensure reasonable reproducibility of associations across cohorts) either positive or negative but with same directionality and at least *Q* ≤ 0.10) with multiple measures of unhealthy aging in three out the five repositories (to ensure repand at the maximum of two associations (total out of the 43 scenarios) with the opposite directionality were first identified. These consisted of 16 features showing consistent positive associations with multiple measures of unhealthy aging and 39 features showing consistent negative associations with multiple measures of unhealthy aging.

### Association analysis between microbiome properties and indices of unhealthy young across various data repositories

Similar to that described above, there were 30 scenarios of unhealthy phenotype in the young across the five data repositories. Association of the 116 microbiome features were performed using a similar manner as above. For the young, 64 features showing multiple associations with the same directionality (either positive or negative but with same directionality and at least *P* ≤ 0.05) with multiple measures of unhealthy aging in three out the five repositories and at the maximum of two associations (out of the 36 scenarios) with the opposite directionality were first identified. These consisted of 26 features showing consistent positive associations with multiple measures of unhealthy aging and 38 features showing consistent negative associations with multiple measures of unhealthy aging.

### Computation of coabundance networks, prevalence, network centrality properties of various species-level taxa

For this investigation, we considered 12 studies consisting of the same 13 study cohorts (‘*n*’ numbers of the individual studies provided in Table 1) considered previously for the age-specific associations of the species-level features with the exception of NU-AGE as it did not contain microbiomes from younger individuals. For each cohort, first the gut microbiome profiles obtained from all older individuals (≥60 years of age) were obtained. We specifically investigated the commonly detected 107 species-level taxa that were identified as described in the previous sections (Fig. 2). Further methodological details of network analysis are provided in Supplementary Note 7.

### Reporting summary

Further information on research design is available in the Nature Portfolio Reporting Summary linked to this article.

### Supplementary information

Supplementary Information Supplementary Notes 1–7 and Figs. 1–15.

Reporting Summary

Supplementary Table 1. All supplementary tables combined into a single worksheet.

Supplementary Data 1. Node list and edge list for Fig. 2.

Supplementary Data 2. Numerical data tables for Fig. 3.

Supplementary Data 3. Node list and edge list for Fig. 5 (large network).

Supplementary Data 4. Numerical data tables for Extended Data Fig. 9.

Supplementary Data 5. Node list and edge list for Extended Data Fig. 10.

Supplementary Data 6. Numerical table for Table 1.

## Data Availability

The study is a meta-analysis of seven major data resources; the sequence data for four of the data resources (with the exception of NU-AGE) are publicly available. For curatedMetagenomicData3 repository, the taxonomic and pathway profiles were already available and hence were downloaded and directly used for the current study. The sequence data for each of the individual study collated as part of the curatedMetagenomicData3 (CMD3) are publicly available, and the corresponding accession numbers can be obtained by downloading the repository at https://waldronlab.io/curatedMetagenomicData/. For the American Gut (AG) project, the filtered, bloom removed OTU biom files and the corresponding metadata were already available at figshare with reference IDs 6137192 and 6137315, respectively^11,36,37^. These profiles were used for the steps of this analyses. For He et al. and LogMPie cohort, the sequence data were available at the European Nucleotide Archive (ENA) (https://www.ebi.ac.uk/ena/) via accession numbers PRJEB18535 and PRJEB25642, respectively, and the metadata available as part of the original publications^38,39^. For Odamaki et al., the sequence data were available at the DDBJ under accession number DRA004160, and the metadata were obtained from the corresponding publication^16^. For the four studies comprising the Irish Shotgun cohorts, the sequence data were already publicly available at the ENA under the accession numbers PRJEB20054 (ref. ^12^), PRJEB15388 (ref. ^13^), PRJEB42304 (ref. ^14^) and PRJEB37017 (ref. ^1^). The starting data and the processed profiles for the NU-AGE data resource, as well as the minimum starting data for each repository that are necessary to interpret, verify and extend the research in the article, are available at https://github.com/tsg-microbiome/AgeMetaAnalysis. All the data corresponding to the NU-AGE dataset used in the current study are uploaded to this GitHub repository. The explanations for the different data resources are provided in the README.md file of this GitHub repository.

## Source data

Source Data Fig. 1. Numerical source data for Fig. 1
Source Data Fig. 2. Individual high-resolution editable network picture for Fig. 2.
Source Data Fig. 3. Individual high-resolution editable images for Fig. 3a,b.
Source Data Fig. 4. Numerical source data for Fig. 4.
Source Data Fig. 5. Individual high-resolution editable network pictures for Fig. 5.
Source Data Extended Data Fig. 2. Numerical source data for Extended Data Fig. 2.
Source Data Extended Data Fig. 3. Numerical source data for Extended Data Fig. 3.
Source Data Extended Data Fig. 4. Numerical source data for Extended Data Fig. 4.
Source Data Extended Data Fig. 5. Numerical source data for Extended Data Fig. 5.
Source Data Extended Data Fig. 6. Numerical source data for Extended Data Fig. 6.
Source Data Extended Data Fig. 7. Numerical source data for Extended Data Fig. 7.
Source Data Extended Data Fig. 8. Numerical source data for Extended Data Fig. 8.
Source Data Extended Data Fig. 9. Individual high-resolution editable forest plots for Extended Data Fig. 9a,b.
Source Data Extended Data Fig. 10. Individual high-resolution editable network pictures for Fig. 5.

## Code availability

All relevant codes (or scripts) used for this analysis are available at https://github.com/tsg-microbiome/AgeMetaAnalysis.
