# Supplementary material for: Toward an improved definition of a healthy microbiome for healthy aging
Source: Nat Aging. 2022 Nov 17;2(11):1054–69. doi: 10.1038/s43587-022-00306-9 (PMC10154212; doi:10.1038/s43587-022-00306-9)
Supplement: Supplementary file 1 — Supplementary Notes 1–7 and Figs. 1–15. [file 43587_2022_306_MOESM1_ESM.pdf]

nature aging

Analysis https://doi.org/10.1038/s43587-022-00306-9

Toward an improved definition of a healthy microbiome for healthy aging

In the format provided by the authors and unedited

Toward an improved definition of a healthy microbiome for healthy ageing

Tarini Shankar Ghosh, Fergus Shanahan, Paul W. O'Toole

Supplementary Figures Document

This document contains Supplementary Notes (S1-S7) and Supplementary Figures S1-S15 along with their legends

All the references mentioned in the Supplementary Texts are referred to in the main manuscript with the same reference order.

Supplementary Notes

SUPPLEMENTARY NOTE S1

Investigating the reproducibility of the previously identified list of Multiple-Disease-Enriched and Multiple-Disease-Depleted taxa (identified in [1]) in the newly included study cohorts

To identify these sets of taxa, our previous study had investigated eight study cohorts covering five diseases [**1**]. We referred to these two groups of 34 disease-enriched and 25 disease-depleted taxa (previously identified by us) as the set of Multiple-Disease-Enriched and Multiple-Disease-Depleted taxa, respectively. In the current study, we had significantly expanded the study dataset with multiple additional study cohorts and disease conditions (11950 additional gut microbiomes from 22 study cohorts) (**Supplementary Table S10**). Thus, first, we re-investigated the disease associations of these disease-linked taxa (identified in (*1*)) in the newly included datasets of the current study (that were not considered in in (*1*) (**See Methods**)). The four data repositories were CMD3 (12 disease conditions), AG (13 disease conditions), He (12 disease conditions) and ISC (1 disease condition namely IBS) (**Supplementary Table S10**). A marker was considered validated if it satisfied either one of the two criteria. Criteria 1: It associated with the expected directionality (positive for disease-enriched and negative for disease-depleted) in greater than two scenarios and in the opposite directionality at the maximum of a two scenario. Criteria 2: It associated with expected directionality in less than or equal to two scenario, but never with the opposite directionality in any of the investigated scenario. Using the above two criteria, for the additional shotgun and 16S-based datasets. There was a strong reproducibility of the disease associations observed for our previously identified list of disease-associated taxa in the newly investigated cohorts. Across all additional datasets, the associations of 69.5% of Multiple-Disease-Positive and 69.4% of the Multiple-Disease-Negative were reproduced. Besides observing a reasonably high reproducibility of the associations of our previously identified disease/health-associated

taxa, we also observed a significant overlap between the replicated members of the Multiple-Disease-Enriched taxa and the group of 22 taxa showing significant positive associations with Kendall Uniqueness (Odds Ratio=14.58; P=3.76e-7, Fishers' exact test). In contrast, the members of the Multiple-Disease-Depleted significantly associated with the taxa group showing significant negative associations with Kendall Uniqueness (Odds Ratio=9.56, P=0.00013, Fishers' exact test) (**Extended Data Fig 5**). Thus, not only are the associations of the majority of multiple markers of health and disease reproducible across data sets, they associate in exactly opposite manners with the Kendall Uniqueness measure.

SUPPLEMENTARY NOTE S2

Descriptions of the different data repositories included in the current study

a/ curatedMetagenomicData3 (referred to as 'CMD3') (10, 41): The curatedMetagenomicData is a repository of more than 19,000 shotgun metagenomic datasets collated from more than 30 different human-associated microbiome studies. All datasets have been processed using the same bioinformatic analysis pipeline consisting of the metaphlan3 taxonomic profiler along with the humann2 functional profiler (41). From within this data repository, we specifically focussed on fecal shotgun metagenomes with available age-information and with subject age greater than 18 years of age. This resulted in a total of 7,966 gut microbiome profiles. This list was subsequently divided into two groups. The first group, containing 5,685 gut microbiome profiles from 21 studies that had subject ages ranging across an age landscape (specifically minimum age ranging from 18-30 years and maximum age >= 65 years), was used for investigating association analysis of microbiome summary indices amongst each other; association of summary indices and abundance of species-level groups with age; for performing network-based investigations as well as; for performing microbiome-disease investigations (Table 1). The second group, consisting of an additional 2,281 gut microbiome

profiles from 16 studies (containing paired control-patient samples from various diseases), was used only for performing microbiome-disease associations for the specific diseases (**Table 1**). Of these, 2,811 gut microbiome profiles were from older individuals from 13 matched diseased-control studies (covering and with age greater than 60 years. These covered 15 diseases, namely, atherosclerotic cardiovascular disease (ACVD), polyps, Clostridioides difficile infection (CDI), cirrhosis, colorectal cancer (CRC), inflammatory bowel diseases (IBD), impaired glucose tolerance (IGT), type-1-diabetes (T1D), type-2-diabetes (T2D), myalgic encephalomyelitis/chronic fatigue syndrome (ME/CFS), migraine, asthma, schizophrenia, soil-transmitted helminths (STH) and Behcet's disease (BD). Of these, the gut microbiomes from older individuals comprised of those from matched controls and patients of 10 major diseases CRC, Polyps, IBD, IGT, T2D, ACVD, STH, CDI, migraine and asthma.

b/ American Gut Project (abbreviated 'AG') (11): The AG data repository consisted of 4575 16S amplicon-based gut (faecal) microbiome profiles, all processed and analyzed as a single cohort. However, as already reported by the authors, certain samples of the American Gut dataset have been affected by blooms or overgrowth of certain oxygen tolerant species (11). These issues have been addressed by the authors. The refined gut microbiome profiles, containing only those samples having a minimum of 1,250 sequences after the removal of "bloom" Operational Taxonomic Units (OTUs) corresponding to aero-tolerant bacteria (as pointed out by the reviewer) and where the OTU representative sequences, were all trimmed to 125 nucleotides in length, are already available at: doi.org/10.6084/m9.fgshare.6137315.v1 and

https://figshare.com/articles/dataset/American_Gut_Project_fecal_sOTU_counts_table/6137192. We utilized these OTU profiles for our analyses. The same set of samples were previously analyzed in an investigation by Wilmanski et al (6). To retain regional homogeneity and life-style related similarities across samples of this cohort, we retained only those samples

originating from US and UK based individuals. These included 1184 samples from older individuals (age greater than 60 years), corresponding to individuals with self-reported information corresponding to 13 different diseases, namely, autism spectrum disorder (ASD), Alzheimer's disease, cardiovascular disease (CVD), cancer, diabetes, CDI, IBD, irritable bowel syndrome (IBS), kidney disease, liver disease, lung disease, migraine and small intestinal bacterial overgrowth (SIBO).

c/ Irish Shotgun Cohorts (henceforth referred to as 'ISC') (1, 12-14): This consisted of an aggregation of 464 gut shotgun metagenomic profiles from four different studies from settled Irish population cohorts. This included 189 samples from the ELDERMET cohort (older Irish individuals, age >= 65 years) (1); 84 and 56 samples from younger settled Irish individuals belonging to ATHLETEMET and the EXERCISEMET cohorts (18-40 years) (12, 13); 133 samples from irritable bowel disease (IBS) patients and matched controls (age ranging from 18 to 66) (14). All samples across the four studies were processed using similar DNA extraction methodologies and the same bioinformatic protocol for profiling the abundances of microbial species. The metadata of the 189 samples of the ELDERMET also contained information pertaining to five different measures of unhealthy aging, namely Functional Independence Measure (FIM), Barthel Score (both positively associated with physical health status and negatively associated with frailty); Mini Mental State Examination (MMSE) scores and Geriatric Depression Score (GDS) (while MMSE score is negatively associated with cognitive impairment, GDS is positively associated with declined mental status) and; Charlson Comorbidity index.

d/ NU-AGE Cohort (henceforth referred to as 'NU-AGE') (15): This repository consisted of faecal microbiome profiles previously investigated in the NU-AGE Mediterranean Diet intervention study. We only included the 610 16S-based faecal microbiome profiles from individuals only at the baseline time-point. This was an older-subject-specific data repository

consisting of individuals with age ranging from 65-79 years of age. There were multiple measures profiled in this cohort for measuring normal/unhealthy aging status including hand grip strength, gait speed, Fried Score (former two being negatively associated with Frailty, with Fried Score a measure of frailty); Constructional Praxis, Babcock Memory, Verbal Fluency (Boston test total scores), MMSE (all negatively associated with Cognitive Impairment) and GDS (a measure of decline in mental well-being). Additionally, there were also measures of inflammatory markers, of which we specifically investigated the levels of pro-inflammatory cytokines hsCRP and IL-17 as there were observed to have a significant association with the beneficial microbiome modulations observed in the original study.

e/ Odamaki Cohort (henceforth referred to as 'Odamaki') (16): This was a Japanese population cohort previously analyzed by Odamaki et al. The original cohort contained 367 16S rRNA gene amplicon based gut microbiome samples with age ranging from infancy till 104 years of age. From this, we specifically focussed on 306 samples obtained from individuals having age ranging from 18 to 104 years (116 of these individuals were older subjects with age > 60 years). This cohort did not have any clinical measure pertaining to the health status.

f/ "He et al" Cohort (henceforth referred to as 'He') (42): This is a Chinese population cohort previously analyzed by He et al. The cohort contained 7,009 16S rRNA gene amplicon based gut microbiomes. The age range of the participants ranged from 18 to 97 years and a total of 2,434 gut microbiomes were from individuals aged >= 60 years. A total of 3,559 gut microbiome profiles originated from apparently non-diseased individuals (encompassing 1024 gut microbiomes from subjects >= 60 years). Of the remaining 3,450 gut microbiomes, the following 12 clinical complications/diseases contained at least 10 patient-derived profiles: atherosclerosis, cholecystitis, colitis, constipations, diarrhoea, fatty liver, gastritis, IBS, kidney stones, rheumatoid arthritis, metabolic syndrome and T2D.

g/ LogMPie Cohort (referred to as 'LogMPie') (43): This is a pan-Indian population cohort consisting of 1,004 16S rRNA gene amplicon based gut microbiome profiles, previously investigated by Dubey et al (43). The age-range of the subjects was from 18 to 65 years.

SUPPLEMENTARY NOTE S3

Description of the approach for computing CLR transformations of microbiome data

The clr-transformations were performed using the clr function of the compositions package (version 2.0.4) in R. To handle zero values, all values were added a pseudo-count of 0.00001 prior to the clr-transformation. Finally, post-transformation for a microbiome, the minimum transformed values obtained for the microbiome (corresponding to the zero count or zero abundance values) were subtracted from the transformed abundances for all features. This correctly reset the the clr-transformed abundances corresponding zero-count features back to zero, while retaining the distribution of the clr-transformed abundances for all non-zero-count features.

SUPPLEMENTARY NOTE S4

Computation of diversity and uniqueness measures

Shannon diversity: Shannon diversities at both the taxonomic and pathway levels were computed using the 'diversity' function of the vegan package in R [47].

Bray-Curtis and Jaccard Uniqueness: Bray-Curtis uniqueness was computed using the same strategy as adopted in the original study by Wilmanski *et al* [6]. For this purpose, for any of the three kinds of profile (genus abundance or species abundance or pathway abundance), within the samples belonging to a given study, we first computed all-versus-all the Bray-Curtis distance matrix using the vegdist function of the vegan R package with 'method="bray"' as the parameter. The vegan package of version 2.5.7 was utilized for all analyses in the current study.

The sample in the given study cohort with the minimum genus-level Bray-Curtis distance to the given sample was identified and the corresponding distance was assigned as the Bray-Curtis uniqueness corresponding to that sample (for that particular profile, namely genus or species or pathway). The same procedure was utilized to compute the Jaccard uniqueness values (for the three kinds of profiles), except by setting the method parameter in the vegdist function to "jaccard". We utilized the raw counts and the total sum scaled abundances for computing the Bray-Curtis and the Jaccard uniqueness measures.

Aitchison Uniqueness: Given any two samples (or microbiomes), the Aitchison distance is computed as the feature-to-feature Euclidean distances between clr-transformed abundances of each feature constituting the two microbiomes. Thus, for this purpose, we first computed an all-versus-all Aitchison distance matrix by providing the clr-transformed abundance matrix of all the samples to the vegdist function of the vegan R package and computing the distances using 'method=euclidean' as the parameter. As described previously, the Aitchison uniqueness for the microbiome was computed as the minimum distance for that sample from all samples from the same study cohort.

Kendall Uniqueness: The Kendall Uniqueness measure (for the three kinds of features) was computed as follows. As for the other uniqueness measures, given the clr-transformed microbiome profile (species/genus/pathway) we first computed all-versus-all sample-to-sample Kendall distance matrices for all samples belonging to each study. For this purpose, the profile (features in rows and samples in columns) was provided as input to the cor.fk function of the pcaPP package (version 1.9.74) of R as:

cor.fk(species or genus or pathway profile for all samples belonging to a given study)

The resultant was a Kendall's-Tau matrix. Each cell of this matrix contained the Kendall's-Tau correlation between two samples based on the similarities in the abundance

patterns of the different features. We specifically utilized the Kendall's-Tau for this purpose rather than using Spearman or Pearson correlation, because of the relatively higher robustness of this measure against sparse datasets as typically observed for microbiome profiles.

SUPPLEMENTARY NOTE S5

Different Distance Measures convey distinct aspects of gut microbiome variation

Bray-Curtis distance measures the variation in the counts (or relative abundances) of the different microbial features (species/genus/pathway) between two microbiomes. Jaccard distance, on the other hand, primarily measures the variation in the detection (rather than the abundance) of various features. The Aitchison distance is similar to Bray-Curtis distances but is especially tuned for investigating compositional data (the data type under which a majority of microbiome data can be classified. It is defined as the Euclidean distances (sum of the squared differences) between the centered-log-ratio (clr) transformed abundances of the different microbiome features (a transformation suited for investigating compositional datasets). Each of the above three uniqueness reflect specific aspects of variations in the abundance and detection of individual species or genera or pathways. Thus, higher values of any of the three uniqueness measures indicates higher variation in terms of the presence or abundance of taxa (or pathways) across the samples (**Extended Data Figure 1**). In contrast, the Kendall Uniqueness measures variations in the relative ranks of different taxa within an individual sample as compared to others from the same cohort. Low Kendall dissimilarity between two microbiomes A and B indicates that features highly abundant in sample A are also highly abundant in Sample B and *vice-versa*, thereby retaining the relative hierarchy in the composition of microbiome structure. Similarly, high Kendall distance between two microbiomes A and C indicates that features highly abundant in microbiome A may not be

highly abundant in C thereby there is a change in the relative hierarchy in the composition of microbiome structure.

SUPPLEMENTARY NOTE S6

Details of the two step procedure adopted for computing associations between different properties

In the first step, given any pair of properties (including the age), we first computed the extent of association between the properties individually within each study using Robust Linear Regression models. The significance of the associations for each model were computed using two-sided Robust F-tests (or Wald-Tests for multiple coefficients as described previously) (50). In the second step, RLM estimates obtained individually within each individual study (now considered as study-specific effect sizes) were then investigated for consistency and significance across studies using the meta-analytic Random Effect Models across the studies as a whole or within groups of studies from the European/North American, East Asian and Other (South Asian, South American/Pacific Islands and African) geographies. The random effect models were computed using the rma function of the metafor package version 3.0.2 in R. However, for certain investigations that required a combined analysis by merging studies, the study name was encoded as 'dummy variable' and associated computed using a simple linear regression computed (lm function of the base R package 4.1.0).

SUPPLEMENTARY NOTE S7

Details of the gut microbiome co-occurrence network computation

Within each of the 12 study cohorts, separate microbial co-abundance networks were computed for the selected taxa for the gut microbiome profiles from the older individuals (age >= 60 years) and those from younger individuals (age < 60 years). For these two types of co-abundance networks, we adopted a two-step compositionality-addressing strategy as described below. First, we computed the Kendall tau values between the clr-transformed abundances of each pair of the 107 species-level taxa. This was obtained using the cor.fk function of the pcaPP package. The p-values and the FDR corrected p-values (or the Q-values) of the associations were computed using corr.p function of the psych package (where in the p-values were corrected on a per-taxa basis). The subsequent species-to-species taxa-level co-abundance network was created by adding an edge between species-level taxa-pairs with Kendall's Tau of greater than 0 and FDR <= 0.1. This was performed using the igraph package version 1.2.8 of R (using the function of graph_from_adjacency_matrix function). The three different centrality measures, namely Betweenness, Degree and Hub-Score were then computed for the older-specific networks and the young-specific networks obtained for each of the 12 individual studies using the different functions within these packages (namely, betweenness, degree and hub_score). Each centrality measure captures a different aspect of the centrality pertaining to a given node (or taxa) of a network. For example, while degree indicates the number of nodes that a given node is connected to (or the total number of taxa a given taxa has co-abundance relationships with), betweenness refers to number of shortest paths (as compared to all possible shortest paths between vertex or node pair) that pass through the given node (in other words, the number of taxa pairs whose co-abundance relationships may be mediated by the given taxa). Hub-score on the other hand is equivalent to the authority score of the taxa (and a generalization of the eigen-vector centrality) that primarily measures the connectedness of the nodes or taxa

connected to the given taxa (where in taxa that are connected to other highly connected taxa are given higher scores). The individual centrality measures for each taxa (or node) obtained were then ranked across all taxa constituting the nodes of a given network.

The two combined networks of edges merging the patterns corresponding to the older-subject-specific and young-specific microbiome sub-types across the 12 different study cohorts were obtained as follows. For each pair of species, we computed the overall association (model estimates and P-value of association) by investigating the individual Kendall Taus obtained for each of the study cohorts using Random Effect Model. These included either the older-subject-specific or young-specific gut microbiome sub-types belonging to the 12 study cohorts. For each species-level taxa, the P-values obtained using Random Effect Models corresponding to each of the other 107 species-level taxa were corrected using Benjamini-Hochberg approach to obtain the FDR (or Q-Value). Species-level-pairs having an overall Random Effects Model estimate of greater than 0 and FDR <= 0.1 (and a positive Kendall Tau across at least 70% of the study cohorts) were identified as having a significant co-abundance relationship and thus a co-abundant edge amongst them. The same strategy was utilized for generating the older-subject-specific and the young-specific consensus co-abundance networks.

For the specific co-abundant hub of putatively beneficial species-level taxa, we also checked if the same edge was reproduced in at least 50% of the other cohorts. Prevalence values of the taxa in this combined network were computed as the percentage of all the older-subject-specific gut microbiomes across all the five data repositories in which they were detected.

Supplementary Figures

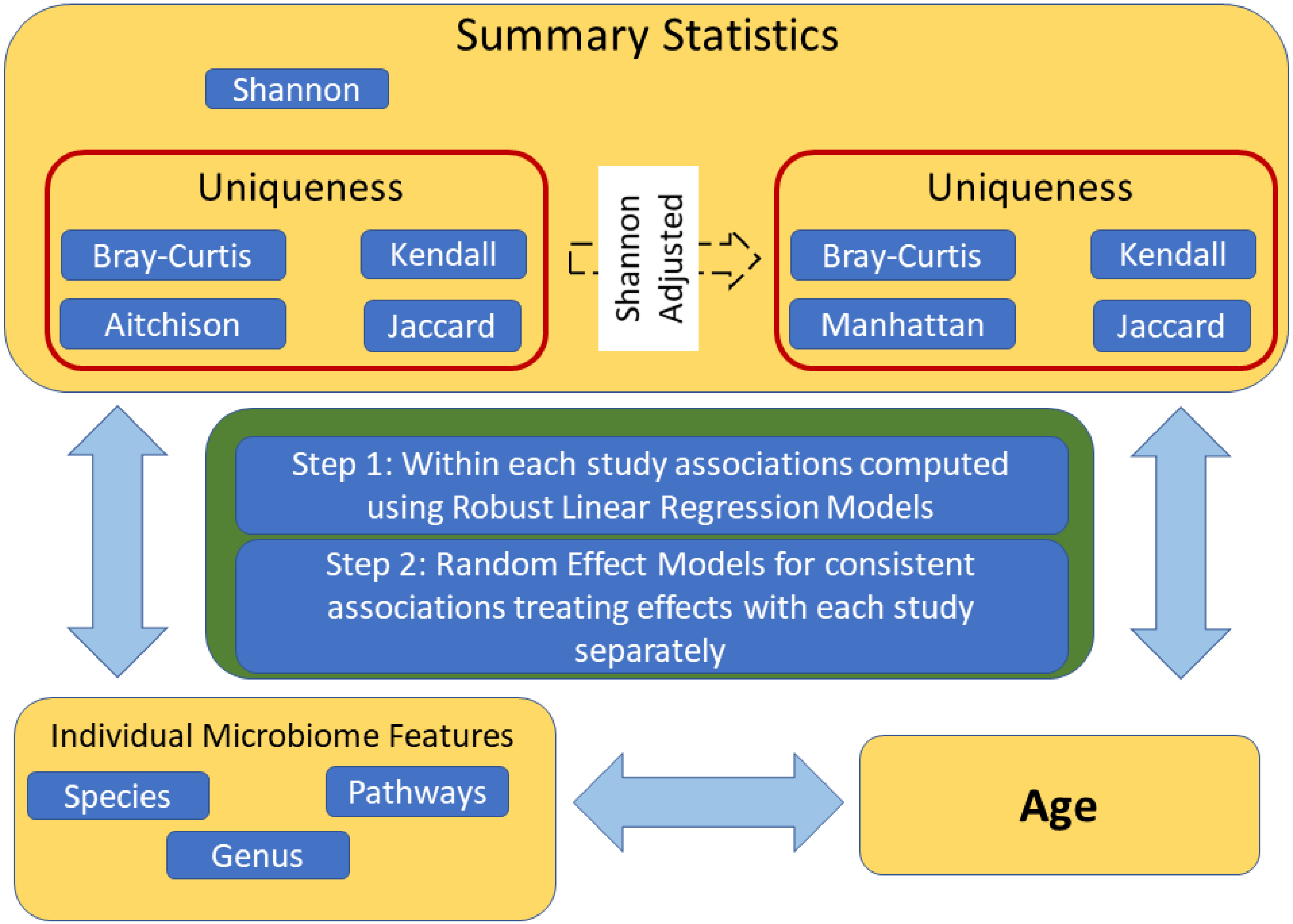

Supplementary Figure S1. Pictorial representation of the two-step Meta-analytic framework utilized for investigating different microbiome properties and age.

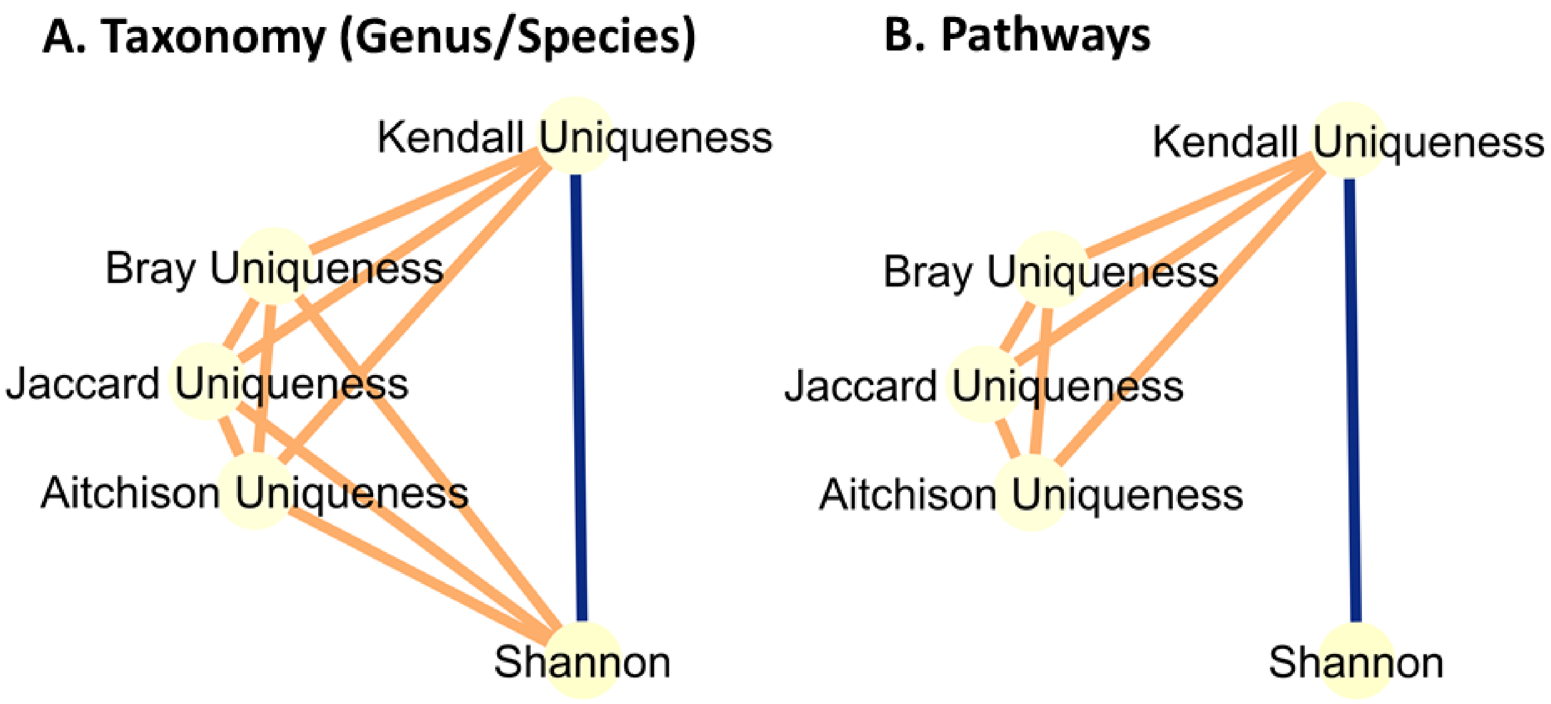

Supplementary Figure S2. Network showing the relationships between different microbiome summary indices at the level of A. Taxonomy and B. Pathways, obtained using the Random Effect Models. An edge between any two properties indicates a significant summarized association between the properties with Q <= 0.05. Positive associations are indicated in orange and negative associations are indicated in dark-blue.

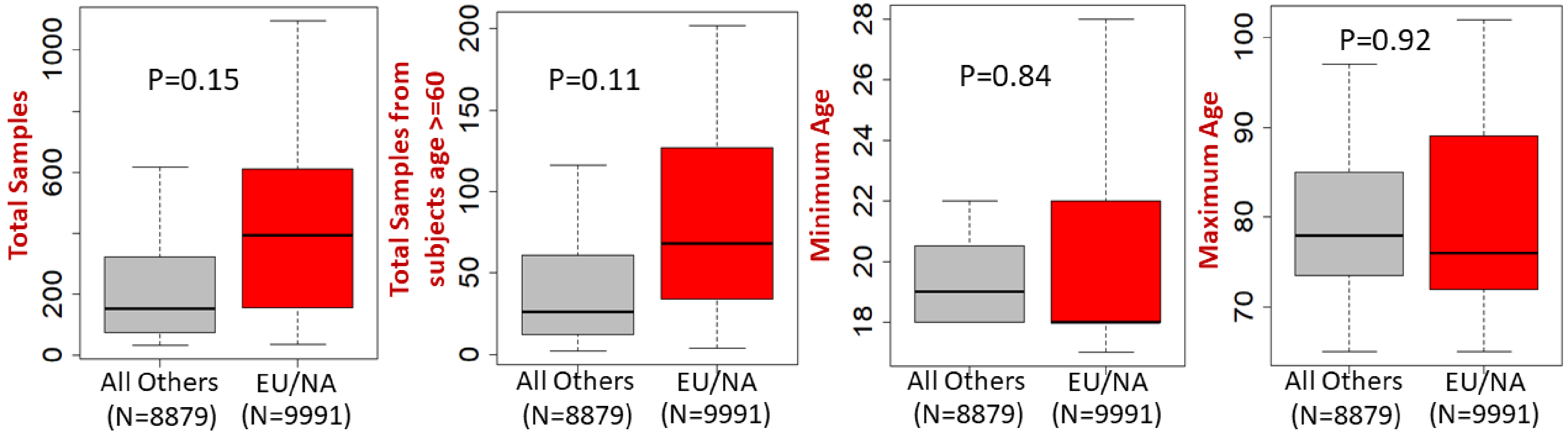

Supplementary Figure S3. Boxplots comparing the total number of samples, total number of samples from older subjects with age >= 60 years, the minimum subject age and the maximum subject age in cohorts from Europe/North America and those from other geographical regions. The p-values of the comparison in the distributions of these values obtained for the two major cohort groups using two-sided Mann-Whitney tests is also computed. Boxes corresponding to the boxplots indicate the inter-quartile range (with the median indicated in bold) of the values and the upper and lower whiskers extend to +1.5 X interquartile range from the third quartile (upper whisker) or to -1.5 X interquartile range from the first quartile (lower whisker).

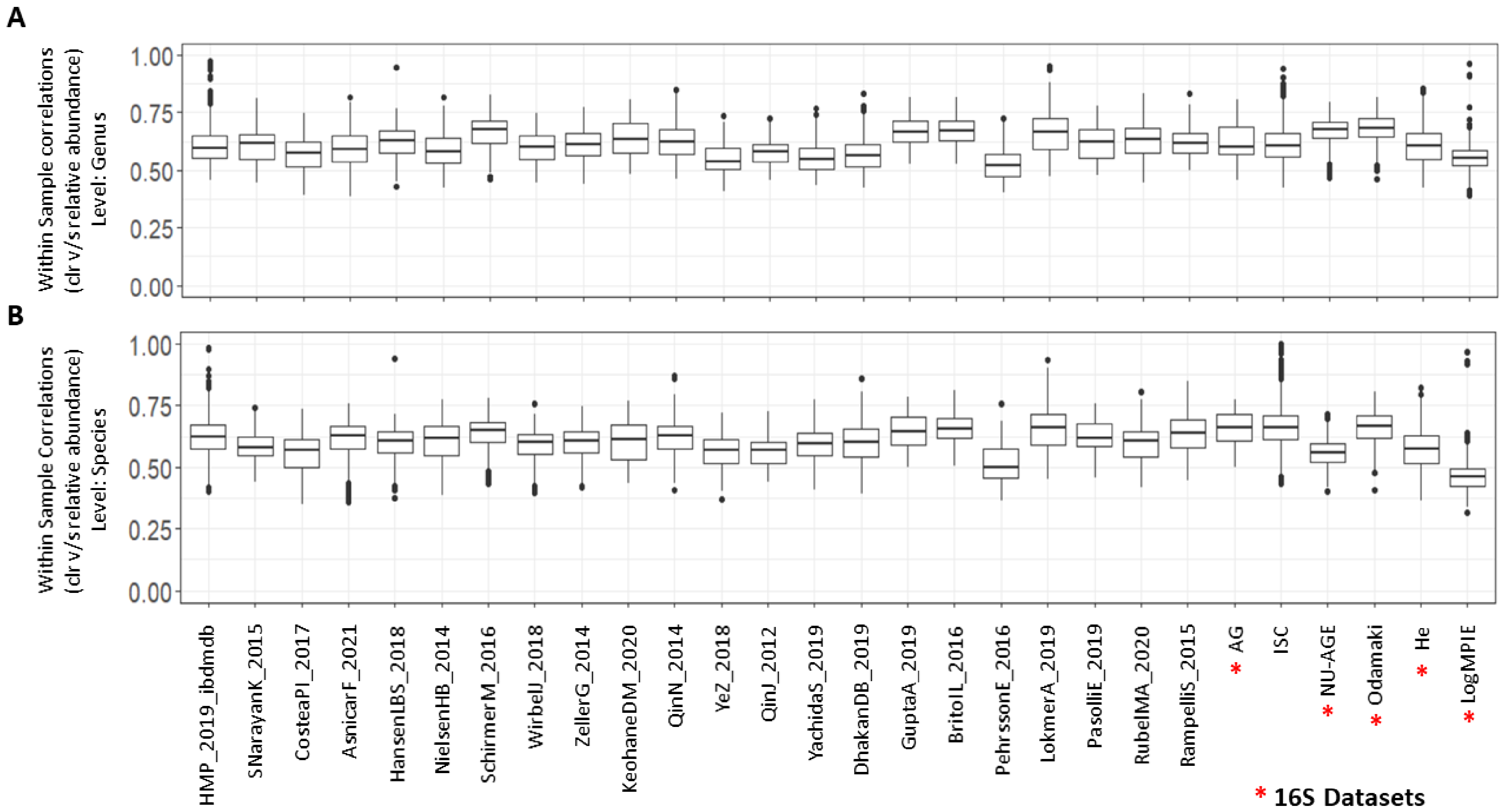

A
B
Within Sample correlations (clr v/s relative abundance) Level: Genus
Within Sample Correlations (clr v/s relative abundance) Level: Species
1.00
0.75
0.50
0.25
0.00
HMP_2019_ibdmdb
SNarayanK_2015
CosteaPI_2017
AsnicarF_2021
HansenLBS_2018
NielsenHB_2014
SchirmerM_2016
WirbelJ_2018
ZellerG_2014
KeohaneDM_2020
QinN_2014
YeZ_2018
QinJ_2012
YachidaS_2019
DhakanDB_2019
GuptaA_2019
BritoIL_2016
PehrssonE_2016
LokmerA_2019
PasolliE_2019
RubelMA_2020
RampelliS_2015
* AG
ISC
* NU-AGE
* Odamaki
* He
* LogMPIE
* 16S Datasets

Supplementary Figure S4. Boxplots showing the distribution of the correlation values between the clr-transformed abundances and the (total sum-scaled) relative abundances (for the same taxa) obtained ***within*** each individual microbiome belonging to the 28 individual studies. A. denotes the correlations between the sample-specific taxa abundances at the genus level. B. denotes the correlations between the sample-specific taxa abundances at the species level. The clr-transformed abundances of the different taxa had a Spearman correlation of greater than 0.50 based upon taxa with relative abundances in more than 75% of the samples across all 28 studies at the genus level and in 27 out of the 28 studies when the taxonomic abundances were profiled at species level. Boxes corresponding to the boxplots indicate the inter-quartile range (with the median indicated in bold) of the values and the upper and lower whiskers extend to +1.5 X interquartile range from the third quartile (upper whisker) or to -1.5 X interquartile range from the first quartile (lower whisker). The number of samples (or gut microbiome profiles) investigated as part of each study cohort are: HMP_2019_ibdmdb:846, SankaranarayananK_2015:37, CosteaPI_2017:201, AsnicarF_2021:1098, HansenLBS_2018:207, NielsenHB_2014:394, SchirmerM_2016:465, WirbelJ_2018:125, ZellerG_2014:156, KeohaneDM_2020:117, QinN_2014:237, YeZ_2018:65, QinJ_2012:342, YachidaS_2019:616, DhakanDB_2019:88, GuptaA_2019:60, BritoIL_2016:154, PehrssonE_2016:120, LokmerA_2019:57, PasolliE_2019:111, RubelMA_2020:156, RampelliS_2015:33, AG:3812, NUAGE:610, ISC:464, He:7009, Odamaki:306, LogMPie:874.

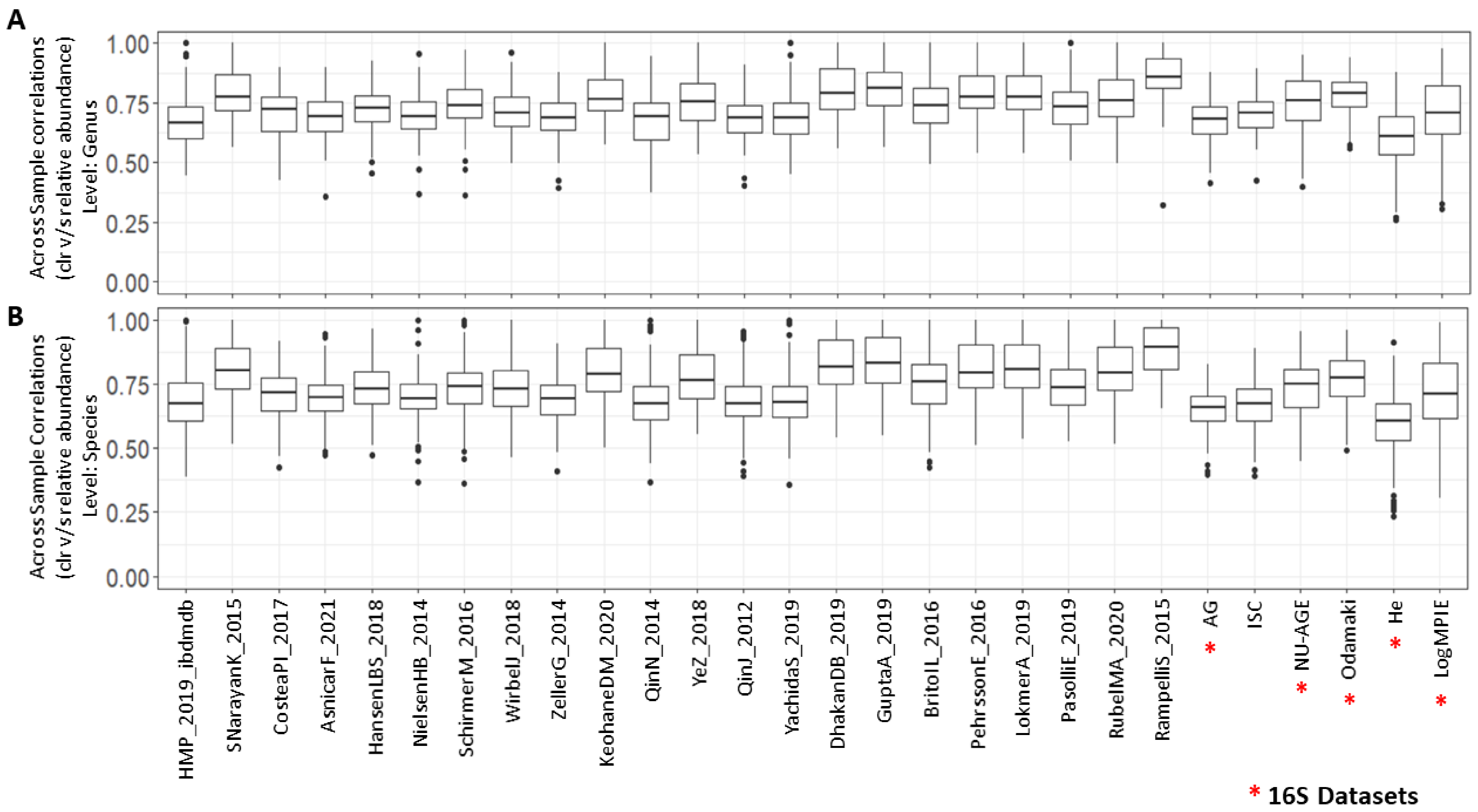

A
B
Across Sample correlations (clr v/s relative abundance) Level: Genus
Across Sample Correlations (clr v/s relative abundance) Level: Species
1.00
0.75
0.50
0.25
0.00
HMP_2019_ibdmdb
SNarayanK_2015
CosteaPI_2017
AsnicarF_2021
HansenLBS_2018
NielsenHB_2014
SchirmerM_2016
WirbelJ_2018
ZellerG_2014
KeohaneDM_2020
QinN_2014
YeZ_2018
QinJ_2012
YachidaS_2019
DhakanDB_2019
GuptaA_2019
BritoIL_2016
PehrssonE_2016
LokmerA_2019
PasolliE_2019
RubelMA_2020
RampelliS_2015
AG *
ISC
NU-AGE *
Odamaki *
He *
LogMPIE *
* 16S Datasets

Supplementary Figure S5. Boxplots showing the distribution of the correlation values between the clr-transformed abundances and the (total sum-scaled) relative abundances of the different taxa ***across*** the gut microbiomes belonging to each of the 28 individual studies. A. denotes the correlations between the sample-specific taxa abundances at the genus level. B. denotes the correlations between the sample-specific taxa abundances at the species level. The clr-tranformed abundances of the different taxa had a spearman correlation of greater than 0.50 with the relative abundances in more than 75% of the taxa across all 28 studies at the genus level and in 27 out of the 28 studies (for all taxa) when the taxonomic abundances were profiled at species level. Boxes corresponding to the boxplots indicate the inter-quartile range (with the median indicated in bold) of the values and the upper and lower whiskers extend to +1.5 X interquartile range from the third quartile (upper whisker) or to -1.5 X interquartile range from the first quartile (lower whisker). The number of samples (or gut microbiome profiles) investigated as part of each study cohort are: HMP_2019_ibdmdb:846, SankaranarayananK_2015:37, CosteaPI_2017:201, AsnicarF_2021:1098, HansenLBS_2018:207, NielsenHB_2014:394, SchirmerM_2016:465, WirbelJ_2018:125, ZellerG_2014:156, KeohaneDM_2020:117, QinN_2014:237, YeZ_2018:65, QinJ_2012:342, YachidaS_2019:616, DhakanDB_2019:88, GuptaA_2019:60, BritoIL_2016:154, PehrssonE_2016:120, LokmerA_2019:57, PasolliE_2019:111, RubelMA_2020:156, RampelliS_2015:33, AG:3812, NUAGE:610, ISC:464, He:7009, Odamaki:306, LogMPie:874.

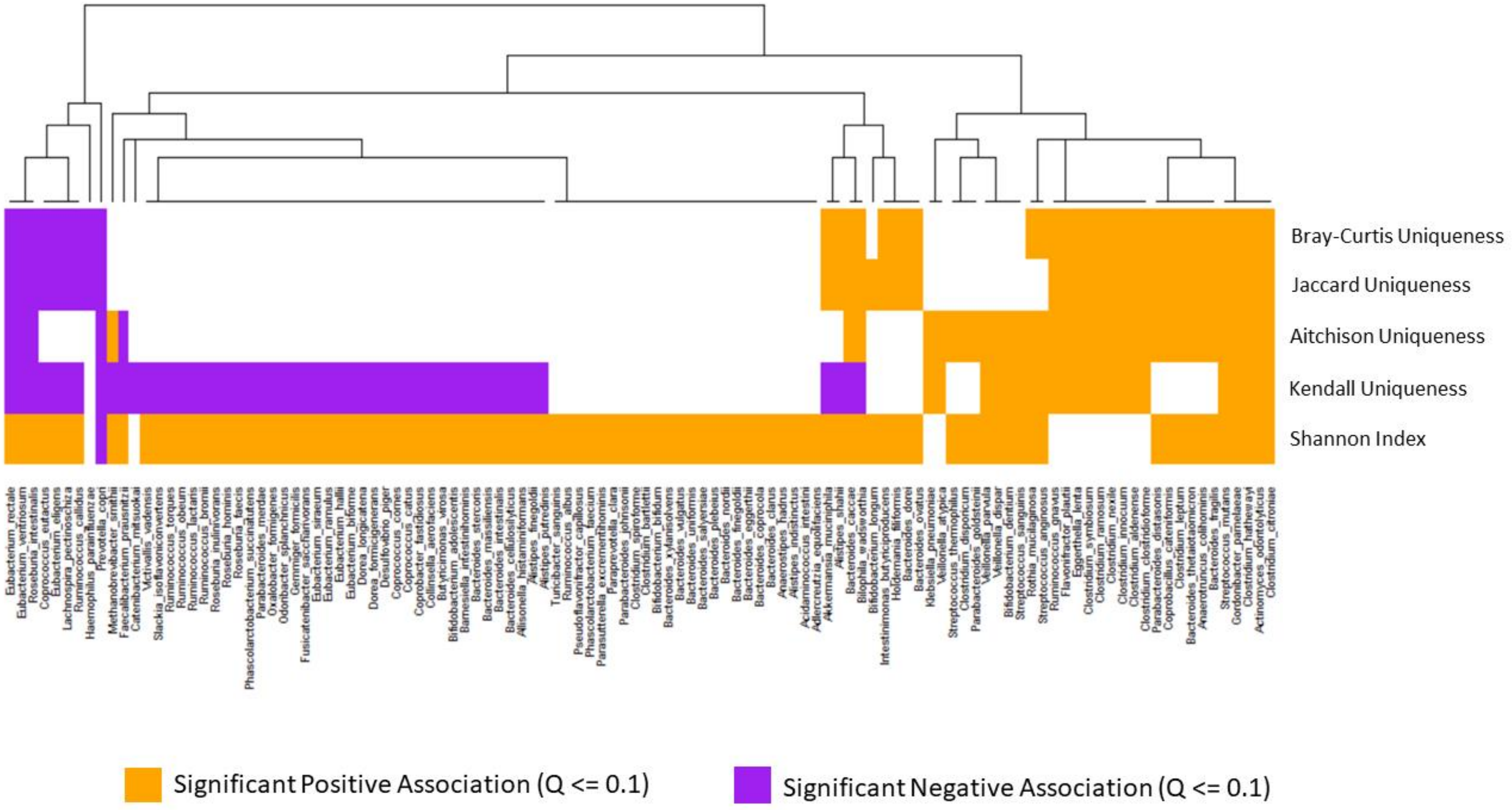

Supplementary Figure S6. Heatmap showing the individual associations of each of the 100 species-level taxa with each of the five microbiome summary statistics in the Random Effect Models based meta-analysis across all the 28 studies. This list includes a subset of taxa the 107 taxa that were identified as in Supplementary Figure S9 that had significant associations with at least one of the five microbiome summary statistics.

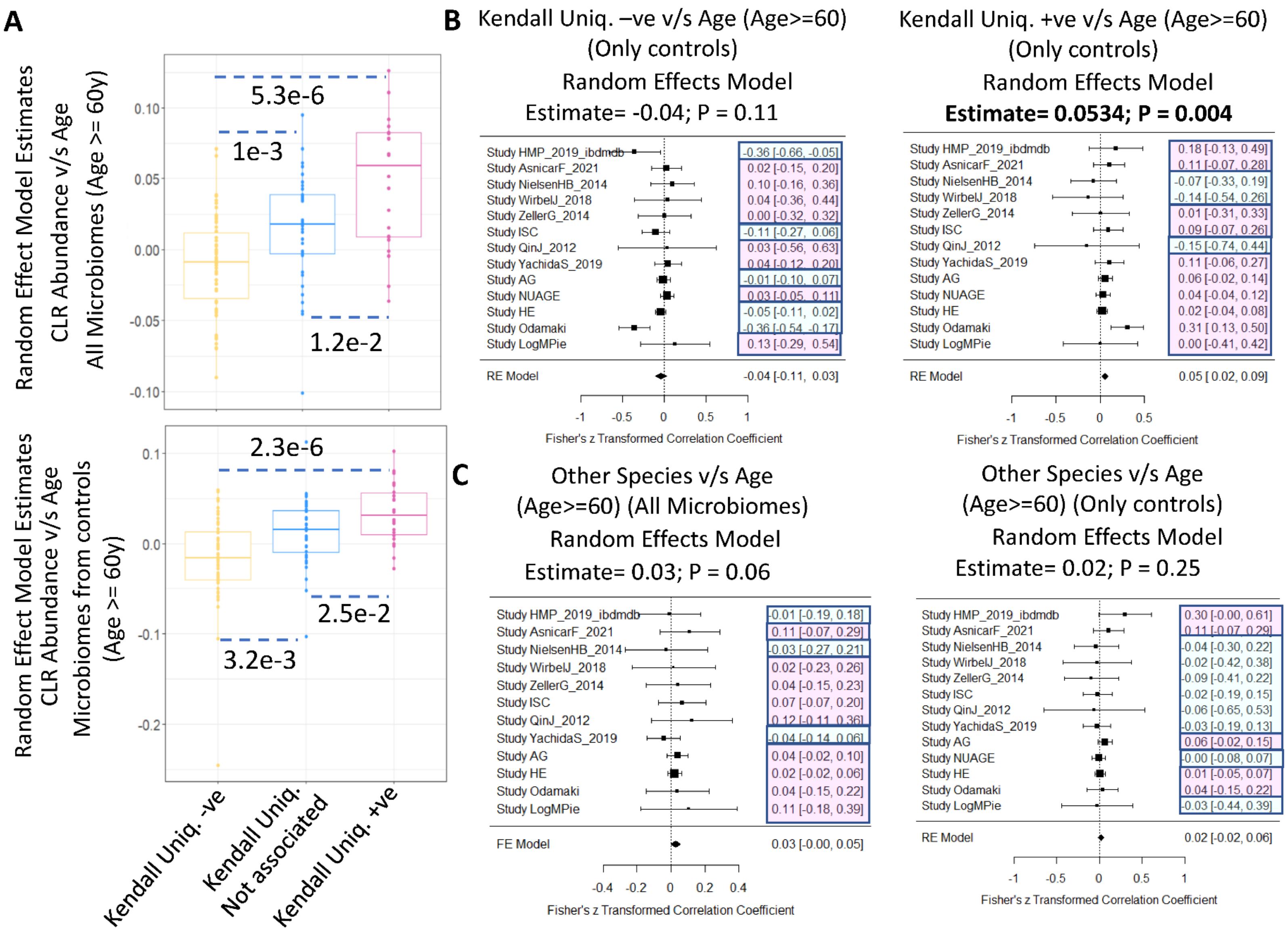

A
Random Effect Model Estimates CLR Abundance v/s Age All Microbiomes (Age >= 60y)
5.3e-6
1e-3
1.2e-2
Random Effect Model Estimates CLR Abundance v/s Age Microbiomes from controls (Age >= 60y)
2.3e-6
2.5e-2
3.2e-3
Kendall Uniq. -ve
Kendall Uniq. Not associated
Kendall Uniq. +ve
B
Kendall Uniq. –ve v/s Age (Age>=60) (Only controls)
Random Effects Model
Estimate= -0.04; P = 0.11
Kendall Uniq. +ve v/s Age (Age>=60) (Only controls)
Random Effects Model
Estimate= 0.0534; P = 0.004
Fisher's z Transformed Correlation Coefficient
C
Other Species v/s Age (Age>=60) (All Microbiomes)
Random Effects Model
Estimate= 0.03; P = 0.06
Other Species v/s Age (Age>=60) (Only controls)
Random Effects Model
Estimate= 0.02; P = 0.25
Fisher's z Transformed Correlation Coefficient

Supplementary Figure S7. A. Boxplot showing the summarized Random Effect Model estimates of the association of the abundances (clr-transformed) of species-level taxa belonging to the different groups (Kendall Uniqueness -ve, n=22; Kendall Uniqueness +ve, n=54, Others n=36) identified in Figure 2 with age (in older subjects with age>=60 years), first considering all the microbiomes (irrespective diseased non-diseased status) (top left) and then considering only the microbiomes from non-diseased controls (bottom left). Both investigations yielded similar patterns. P-values computed using two-sided Mann-Whitney Tests between the estimates corresponding to each pair of groups are also indicated. Boxes corresponding to the boxplots indicate the inter-quartile range (with the median indicated in bold) of the values and the upper and lower whiskers extend to +1.5 X interquartile range from the third quartile (upper whisker) or to -1.5 X interquartile range from the first quartile (lower whisker). **B.** Forest plots showing the results of the Random Effect models-based association analysis of the grouped (mean of the range-scaled) abundance of the taxa showing negative association with Kendall uniqueness and the taxa group showing positive association with Kendall uniqueness with age in only the microbiomes from non-diseased control subjects with age >= 60 years. Here, we detected a significant increase of the generally disease-associated species with age in even the non-diseased controls (a trend replicated ~ 70% of the individual studies). **C.** Forest plots showing the association of the grouped abundance of the other taxa (not associated with Kendall uniqueness) with age post 60 years first considering all microbiomes and then by considering only those microbiomes belonging to the non-diseased controls. For both (B) and (C), Random Effect Model utilizes obtain two-sided p-values of association for the summary effect sizes. The number of samples (or gut microbiome) (n) corresponding to the different studies are: AG:1023, AsnicarF_2021:127, HE:2434, HMP_2019_ibdmdb:117, ISC:202, LogMPie:51, NielsenHB_2014:68, NUAGE:610, Odamaki:116, QinJ_2012:71, WirbelJ_2018:67, YachidaS_2019:393, ZellerG_2014:109.

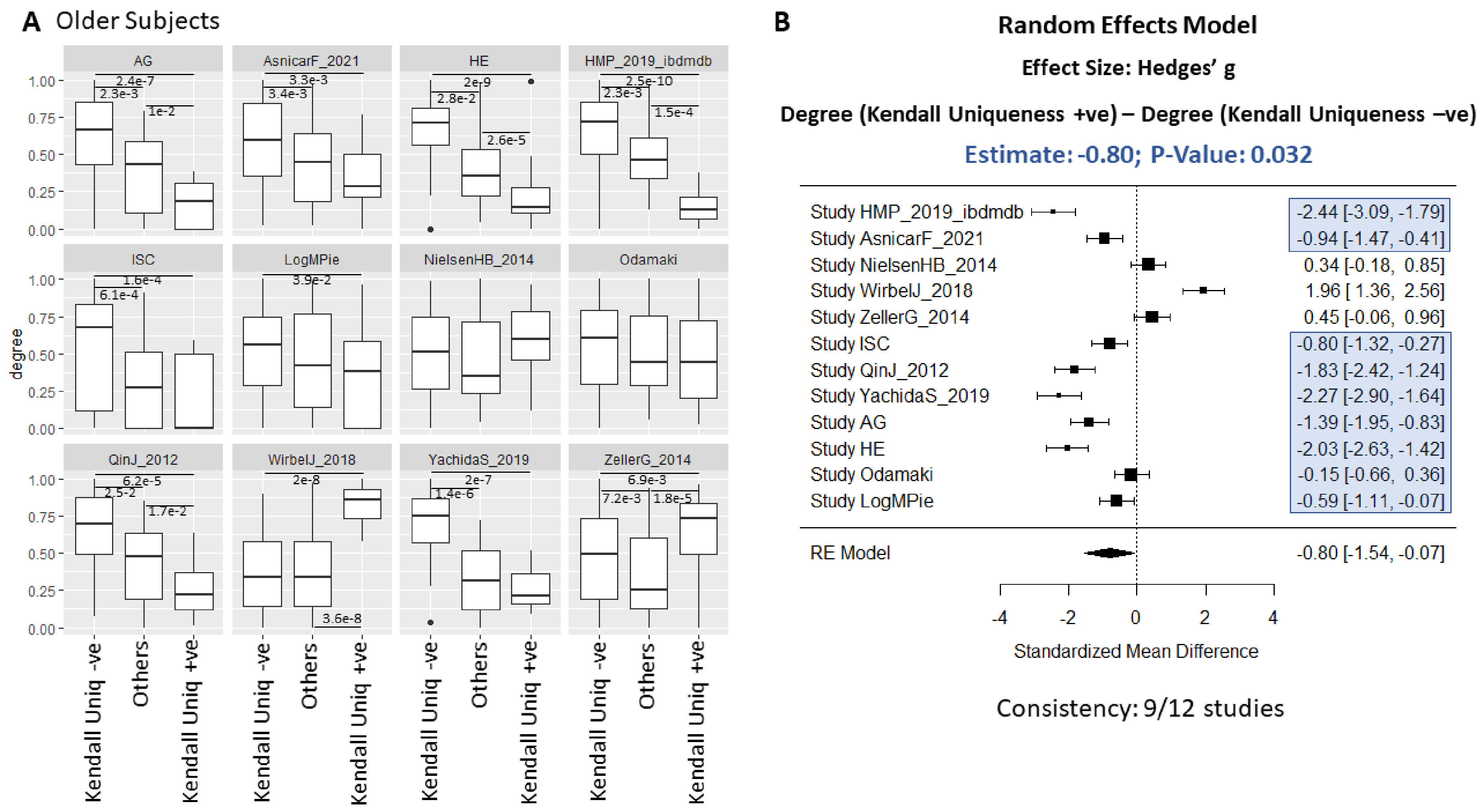
A Older Subjects
AG
AsnicarF_2021
HE
HMP_2019_ibdmdb
ISC
LogMPie
NielsenHB_2014
Odamaki
QinJ_2012
WirbelJ_2018
YachidaS_2019
ZellerG_2014
degree
Kendall Uniq -ve
Others
Kendall Uniq +ve
B
Random Effects Model
Effect Size: Hedges' g
Degree (Kendall Uniqueness +ve) – Degree (Kendall Uniqueness –ve)
Estimate: -0.80; P-Value: 0.032
Study HMP_2019_ibdmdb -2.44 [-3.09, -1.79]
Study AsnicarF_2021 -0.94 [-1.47, -0.41]
Study NielsenHB_2014 0.34 [-0.18, 0.85]
Study WirbelJ_2018 1.96 [1.36, 2.56]
Study ZellerG_2014 0.45 [-0.06, 0.96]
Study ISC -0.80 [-1.32, -0.27]
Study QinJ_2012 -1.83 [-2.42, -1.24]
Study YachidaS_2019 -2.27 [-2.90, -1.64]
Study AG -1.39 [-1.95, -0.83]
Study HE -2.03 [-2.63, -1.42]
Study Odamaki -0.15 [-0.66, 0.36]
Study LogMPie -0.59 [-1.11, -0.07]
RE Model -0.80 [-1.54, -0.07]
Standardized Mean Difference
Consistency: 9/12 studies

Supplementary Figure S8. **A.** Boxplot comparing the degrees of the three groups of species-level taxa (Kendall Uniqueness -ve, n=22; Kendall Uniqueness +ve, n=54, Others n=36) in the older-subject-specific co-occurrence networks obtained for the 12 different studies considered for the network-based analysis. Horizontal bars between boxes indicate the p-values of significance for the comparison within the different groups using dunns' test (two-sided) after adjusting for multiple comparisons using Benjamini-Hochberg. Individually, in eight out of the 12 study cohorts, the Kendall Uniqueness -ve species group exhibited significantly higher (at least with Q <= 0.05) degree than the Kendall Uniqueness +ve species group. This is further shown in B. **B.** Forest plot of the Random Effects Model meta-analysis indicating the significant degree difference between two groups of taxa in the older-subject-specific networks across the 12 studies. The summarized estimates of the model along p-value are also indicated. The significant overall negative effect size further indicates a higher degree for the Kendall Uniqueness -ve group as compared to the Kendall Uniqueness +ve taxa. Consistency indicated here denotes that the pattern of overall negative effect size was replicated within nine out of the 12 individual studies. Boxes corresponding to the boxplots indicate the inter-quartile range (with the median indicated in bold) of the values and the upper and lower whiskers extend to +1.5 X interquartile range from the third quartile (upper whisker) or to -1.5 X interquartile range from the first quartile (lower whisker). The number of samples (or gut microbiome) (n) corresponding to the different studies are: AG:1023, AsnicarF_2021:127, HE:2434, HMP_2019_ibdmdb:117, ISC:202, LogMPie:51, NielsenHB_2014:68, NUAGE:610, Odamaki:116, QinJ_2012:71, WirbelJ_2018:67, YachidaS_2019:393, ZellerG_2014:109. In (B), Two-sided P-values for the Random Effect Model were computed using permutation tests of association for the summary effect sizes.

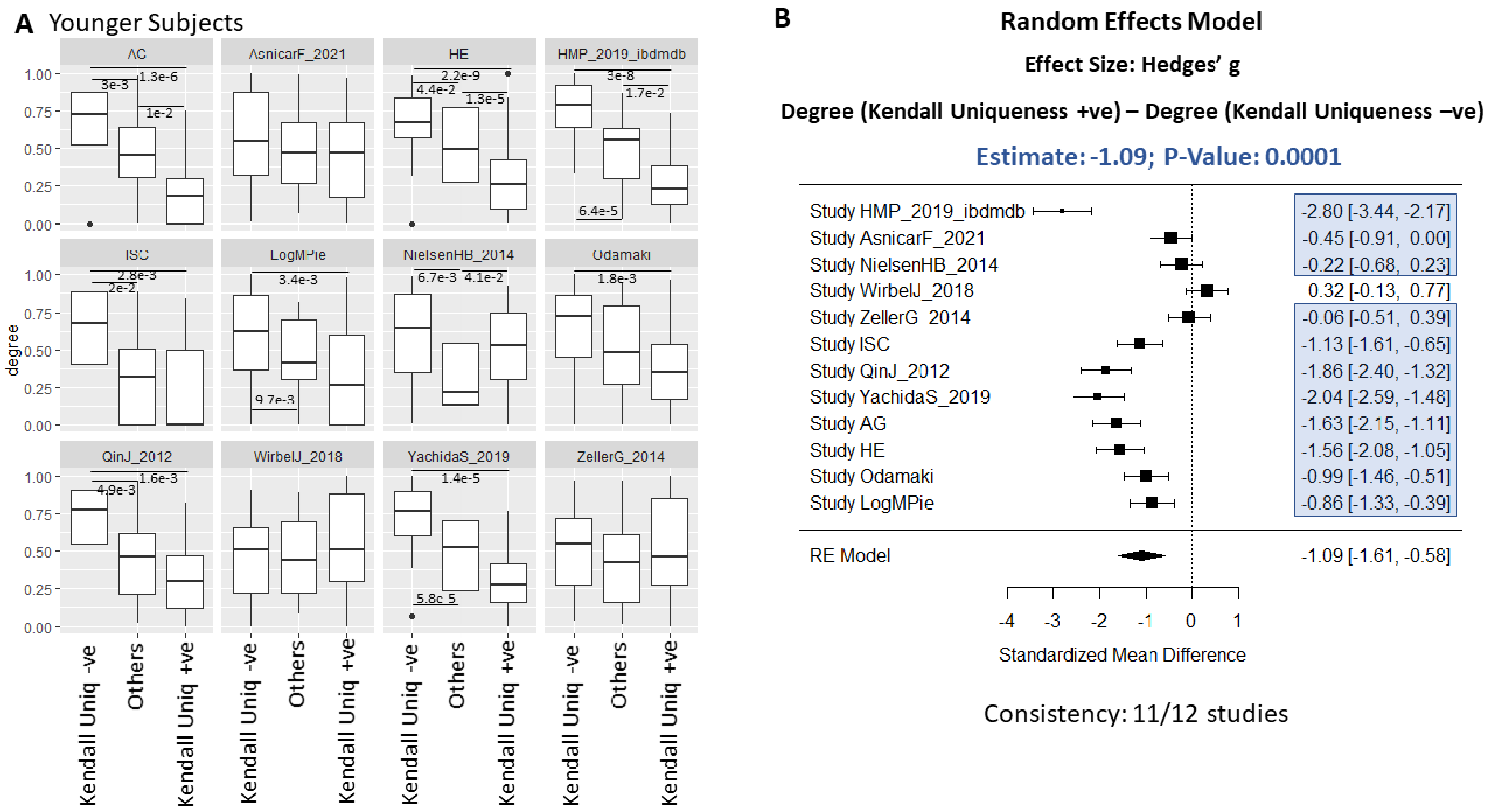
A Younger Subjects
AG
AsnicarF_2021
HE
HMP_2019_ibdmdb
ISC
LogMPie
NielsenHB_2014
Odamaki
QinJ_2012
WirbelJ_2018
YachidaS_2019
ZellerG_2014
3e-3
1.3e-6
1e-2
2.2e-9
4.4e-2
1.3e-5
3e-8
1.7e-2
6.4e-5
2.8e-3
2e-2
3.4e-3
9.7e-3
6.7e-3
4.1e-2
1.8e-3
4.9e-3
1.6e-3
1.4e-5
5.8e-5
degree
1.00
0.75
0.50
0.25
0.00
Kendall Uniq -ve
Others
Kendall Uniq +ve
B
Random Effects Model
Effect Size: Hedges' g
Degree (Kendall Uniqueness +ve) – Degree (Kendall Uniqueness –ve)
Estimate: -1.09; P-Value: 0.0001
Study HMP_2019_ibdmdb -2.80 [-3.44, -2.17]
Study AsnicarF_2021 -0.45 [-0.91, 0.00]
Study NielsenHB_2014 -0.22 [-0.68, 0.23]
Study WirbelJ_2018 0.32 [-0.13, 0.77]
Study ZellerG_2014 -0.06 [-0.51, 0.39]
Study ISC -1.13 [-1.61, -0.65]
Study QinJ_2012 -1.86 [-2.40, -1.32]
Study YachidaS_2019 -2.04 [-2.59, -1.48]
Study AG -1.63 [-2.15, -1.11]
Study HE -1.56 [-2.08, -1.05]
Study Odamaki -0.99 [-1.46, -0.51]
Study LogMPie -0.86 [-1.33, -0.39]
RE Model -1.09 [-1.61, -0.58]
-4
-3
-2
-1
0
1
Standardized Mean Difference
Consistency: 11/12 studies

Supplementary Figure S9. **A.** Boxplot comparing the degrees of the three groups of species-level taxa (Kendall Uniqueness -ve, n=22; Kendall Uniqueness +ve, n=54, Others n=36) in the young-specific co-occurrence networks obtained for the 12 different studies considered for the network-based analysis. Horizontal bars between boxes indicate the p-values of significance for the comparison within the different groups using dunns' test (two-sided) after adjusting for multiple comparisons using Benjamini-Hochberg. Individually, in eight out of the 12 study cohorts, the Kendall Uniqueness -ve species group exhibited significantly higher (at least with Q <= 0.05) degree than the Kendall Uniqueness +ve species group. This is further shown in B. **B.** Forest plot of the Random Effects Model meta-analysis indicating the significant degree difference between two groups of taxa in the young-specific networks across the 12 studies. The summarized estimates of the model along p-value are also indicated. The significant negative effect size further indicates a significant higher degree for the Kendall Uniqueness -ve group as compared to the Kendall Uniqueness +ve taxa. Across 11 of the 12 studies, a negative effect size was observed. Consistency indicated here denotes that the pattern of overall negative effect size was replicated within 11 out of the 12 individual studies. Boxes corresponding to the boxplots indicate the inter-quartile range (with the median indicated in bold) of the values and the upper and lower whiskers extend to +1.5 X interquartile range from the third quartile (upper whisker) or to -1.5 X interquartile range from the first quartile (lower whisker). The number of samples (or gut microbiome) (n) corresponding to the different studies are AG:2789, AsnicarF_2021:971, HE:4575, HMP_2019_ibdmdb:729, ISC:262, LogMPie:823, NielsenHB_2014:326, Odamaki:190, QinJ_2012:271, WirbelJ_2018:58, YachidaS_2019:223, ZellerG_2014:47. In (B), Two-sided P-values for the Random Effect Model were computed using permutation tests of association for the summary effect sizes.

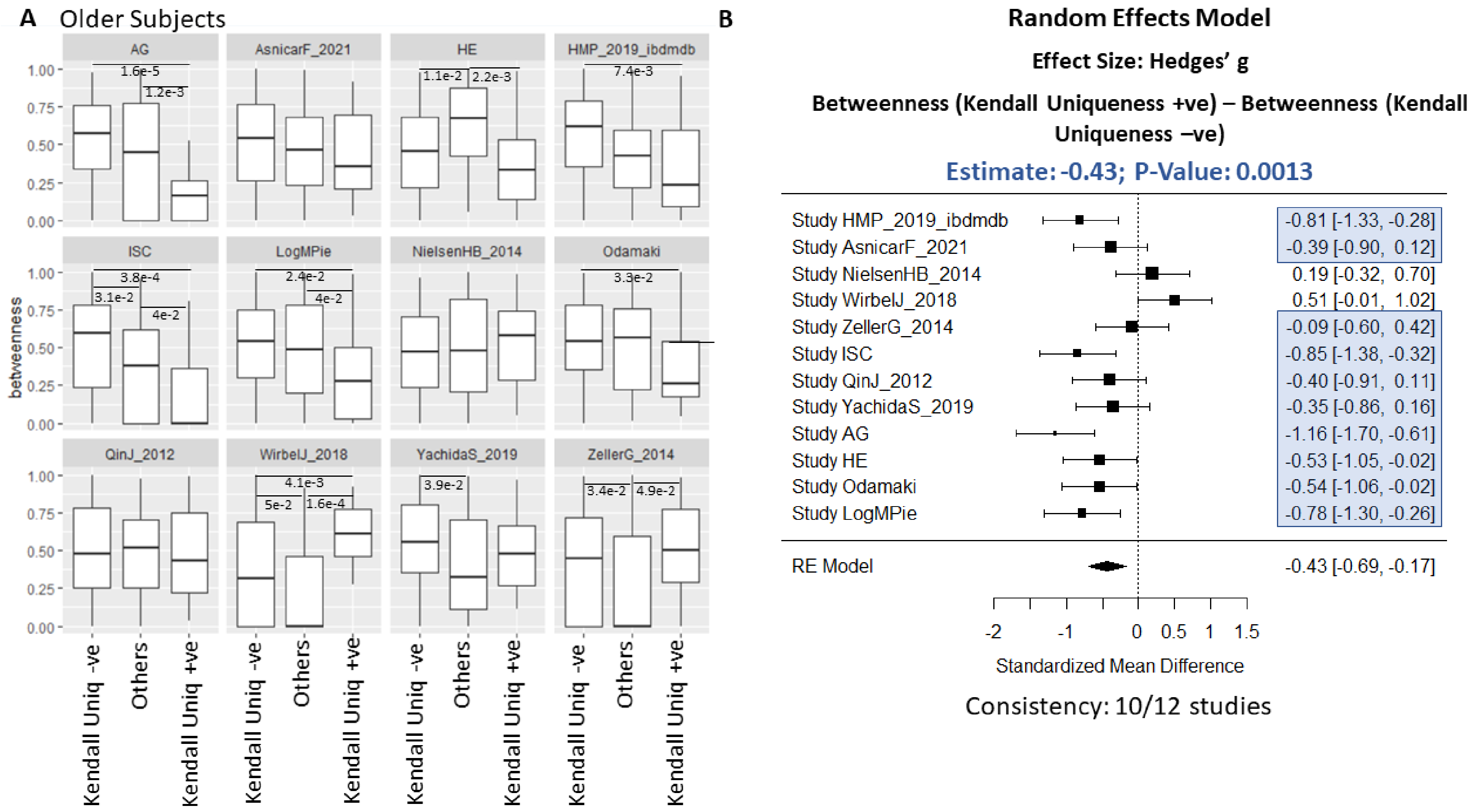
A Older Subjects
AG
AsnicarF_2021
HE
HMP_2019_ibdmdb
ISC
LogMPie
NielsenHB_2014
Odamaki
QinJ_2012
WirbelJ_2018
YachidaS_2019
ZellerG_2014
1.6e-5
1.2e-3
1.1e-2
2.2e-3
7.4e-3
3.8e-4
3.1e-2
4e-2
2.4e-2
4e-2
3.3e-2
4.1e-3
5e-2
1.6e-4
3.9e-2
3.4e-2
4.9e-2
betweenness
0.00
0.25
0.50
0.75
1.00
Kendall Uniq -ve
Others
Kendall Uniq +ve
B
Random Effects Model
Effect Size: Hedges' g
Betweenness (Kendall Uniqueness +ve) – Betweenness (Kendall Uniqueness –ve)
Estimate: -0.43; P-Value: 0.0013
Study HMP_2019_ibdmdb -0.81 [-1.33, -0.28]
Study AsnicarF_2021 -0.39 [-0.90, 0.12]
Study NielsenHB_2014 0.19 [-0.32, 0.70]
Study WirbelJ_2018 0.51 [-0.01, 1.02]
Study ZellerG_2014 -0.09 [-0.60, 0.42]
Study ISC -0.85 [-1.38, -0.32]
Study QinJ_2012 -0.40 [-0.91, 0.11]
Study YachidaS_2019 -0.35 [-0.86, 0.16]
Study AG -1.16 [-1.70, -0.61]
Study HE -0.53 [-1.05, -0.02]
Study Odamaki -0.54 [-1.06, -0.02]
Study LogMPie -0.78 [-1.30, -0.26]
RE Model -0.43 [-0.69, -0.17]
-2 -1 0 0.5 1 1.5
Standardized Mean Difference
Consistency: 10/12 studies

Supplementary Figure S10. **A.** Boxplot comparing the betweenness of the three groups of species-level taxa (Kendall Uniqueness -ve, n=22; Kendall Uniqueness +ve, n=54, Others n=36) in the older-subject-specific co-occurrence networks obtained for the 12 different studies considered for the network-based analysis. Horizontal bars between boxes indicate the p-values of significance for the comparison within the different groups using dunns' test (two-sided) after adjusting for multiple comparisons using Benjamini-Hochberg. Individually, in six out of the 12 study cohorts, the Kendall Uniqueness -ve species group exhibited significantly higher betweenness (at least with Q <= 0.05) than the Kendall Uniqueness +ve species group. In three other cohorts, also a non-significant increase was observed. This was further enforced in the Random Effect Model meta-analysis. **B.** Forest plot of the Random Effects Model meta-analysis indicating the significant betweenness difference between two groups of taxa in the older-subject-specific networks across the 12 studies. The summarized estimates of the model along p-value are also indicated. The significant negative effect size further indicates a higher betweenness for the Kendall Uniqueness -ve group as compared to the Kendall Uniqueness +ve taxa. Across 10 of the 12 studies, a negative effect size was observed. Consistency indicated here denotes that the pattern of overall negative effect size was replicated within 10 out of the 12 individual studies. Boxes corresponding to the boxplots indicate the inter-quartile range (with the median indicated in bold) of the values and the upper and lower whiskers extend to +1.5 X interquartile range from the third quartile (upper whisker) or to -1.5 X interquartile range from the first quartile (lower whisker). The number of samples (or gut microbiome) (n) corresponding to the different studies are: AG:1023, AsnicarF_2021:127, HE:2434, HMP_2019_ibdmdb:117, ISC:202, LogMPie:51, NielsenHB_2014:68, NUAGE:610, Odamaki:116, QinJ_2012:71, WirbelJ_2018:67, YachidaS_2019:393, ZellerG_2014:109. In (B), Two-sided P-values for the Random Effect Model were computed using permutation tests of association for the summary effect sizes.

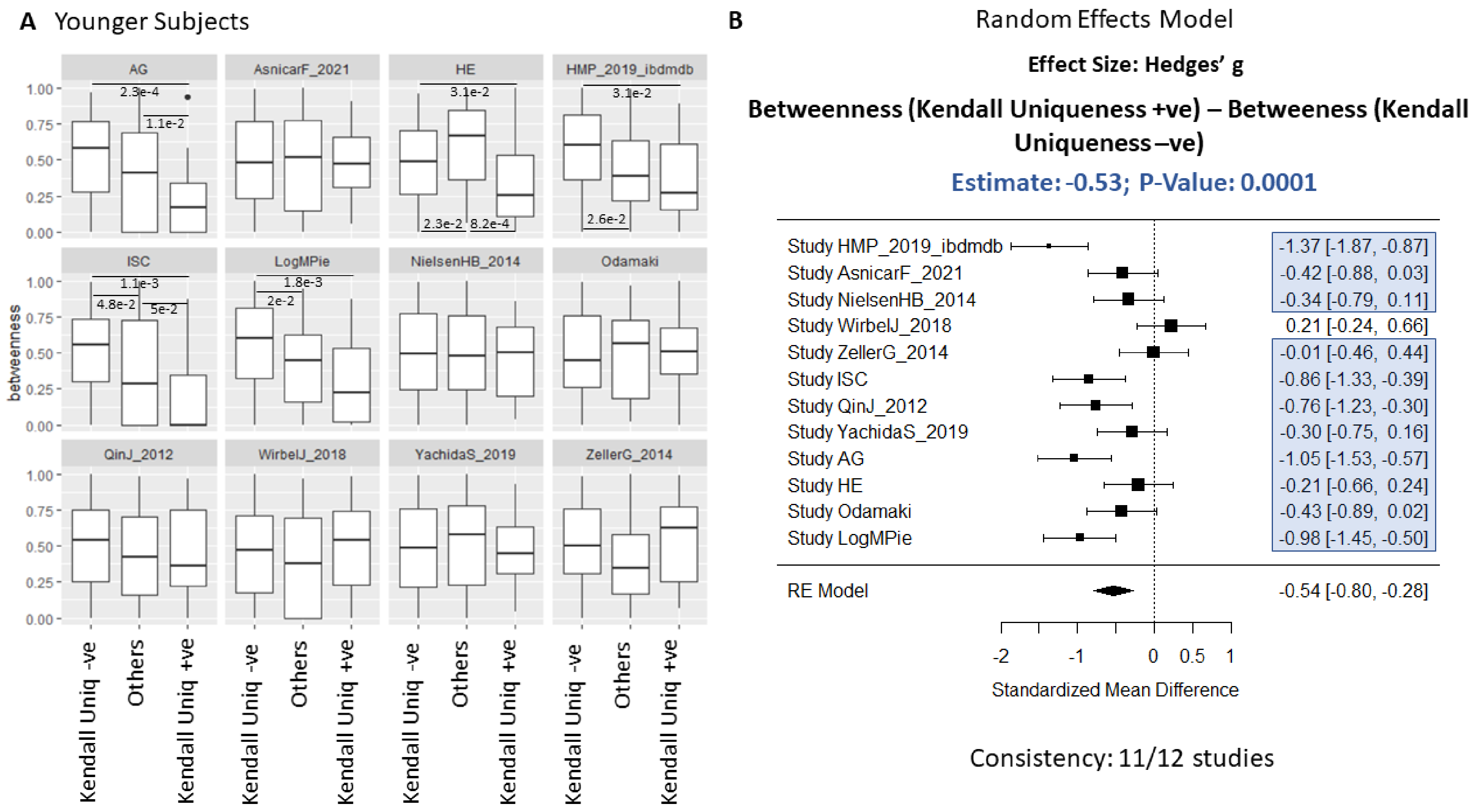
A Younger Subjects
AG
AsnicarF_2021
HE
HMP_2019_ibdmdb
ISC
LogMPie
NielsenHB_2014
Odamaki
QinJ_2012
WirbelJ_2018
YachidaS_2019
ZellerG_2014
betweenness
2.3e-4
1.1e-2
3.1e-2
2.3e-2
8.2e-4
2.6e-2
1.1e-3
4.8e-2
5e-2
1.8e-3
2e-2
Kendall Uniq -ve
Others
Kendall Uniq +ve
B
Random Effects Model
Effect Size: Hedges' g
Betweenness (Kendall Uniqueness +ve) – Betweeness (Kendall Uniqueness –ve)
Estimate: -0.53; P-Value: 0.0001
Study HMP_2019_ibdmdb -1.37 [-1.87, -0.87]
Study AsnicarF_2021 -0.42 [-0.88, 0.03]
Study NielsenHB_2014 -0.34 [-0.79, 0.11]
Study WirbelJ_2018 0.21 [-0.24, 0.66]
Study ZellerG_2014 -0.01 [-0.46, 0.44]
Study ISC -0.86 [-1.33, -0.39]
Study QinJ_2012 -0.76 [-1.23, -0.30]
Study YachidaS_2019 -0.30 [-0.75, 0.16]
Study AG -1.05 [-1.53, -0.57]
Study HE -0.21 [-0.66, 0.24]
Study Odamaki -0.43 [-0.89, 0.02]
Study LogMPie -0.98 [-1.45, -0.50]
RE Model -0.54 [-0.80, -0.28]
Standardized Mean Difference
Consistency: 11/12 studies

Supplementary Figure S11. **A.** Boxplot comparing the betweenness of the three groups of species-level taxa (Kendall Uniqueness -ve, n=22; Kendall Uniqueness +ve, n=54, Others n=36) in the young-specific co-occurrence networks obtained for the 12 different studies considered for the network-based analysis. Horizontal bars between boxes indicate the p-values of significance for the comparison within the different groups using dunns' test (two-sided) after adjusting for multiple comparisons using Benjamini-Hochberg. Individually, in five out of the 12 study cohorts, the Kendall Uniqueness -ve species group exhibited significantly higher betweenness (at least with Q <= 0.05) than the Kendall Uniqueness +ve species group. In three other cohorts, also a non-significant increase was observed. This was further enforced in the Random Effect Model meta-analysis. **B.** Forest plot of the Random Effects Model meta-analysis indicating the significant betweenness difference between two groups of taxa in the young-specific networks across the 12 studies. The summarized estimates of the model along p-value are also indicated. The significant negative effect size further indicates a higher betweenness for the Kendall Uniqueness -ve group as compared to the Kendall Uniqueness +ve taxa. Within 11 of the 12 studies, a negative effect size pattern was replicated. Boxes corresponding to the boxplots indicate the inter-quartile range (with the median indicated in bold) of the values and the upper and lower whiskers extend to +1.5 X interquartile range from the third quartile (upper whisker) or to -1.5 X interquartile range from the first quartile (lower whisker). The number of samples (or gut microbiome) (n) corresponding to the different studies are AG:2789, AsnicarF_2021:971, HE:4575, HMP_2019_ibdmdb:729, ISC:262, LogMPie:823, NielsenHB_2014:326, Odamaki:190, QinJ_2012:271, WirbelJ_2018:58, YachidaS_2019:223, ZellerG_2014:47. In (B), Two-sided P-values for the Random Effect Model were computed using permutation tests of association for the summary effect sizes.

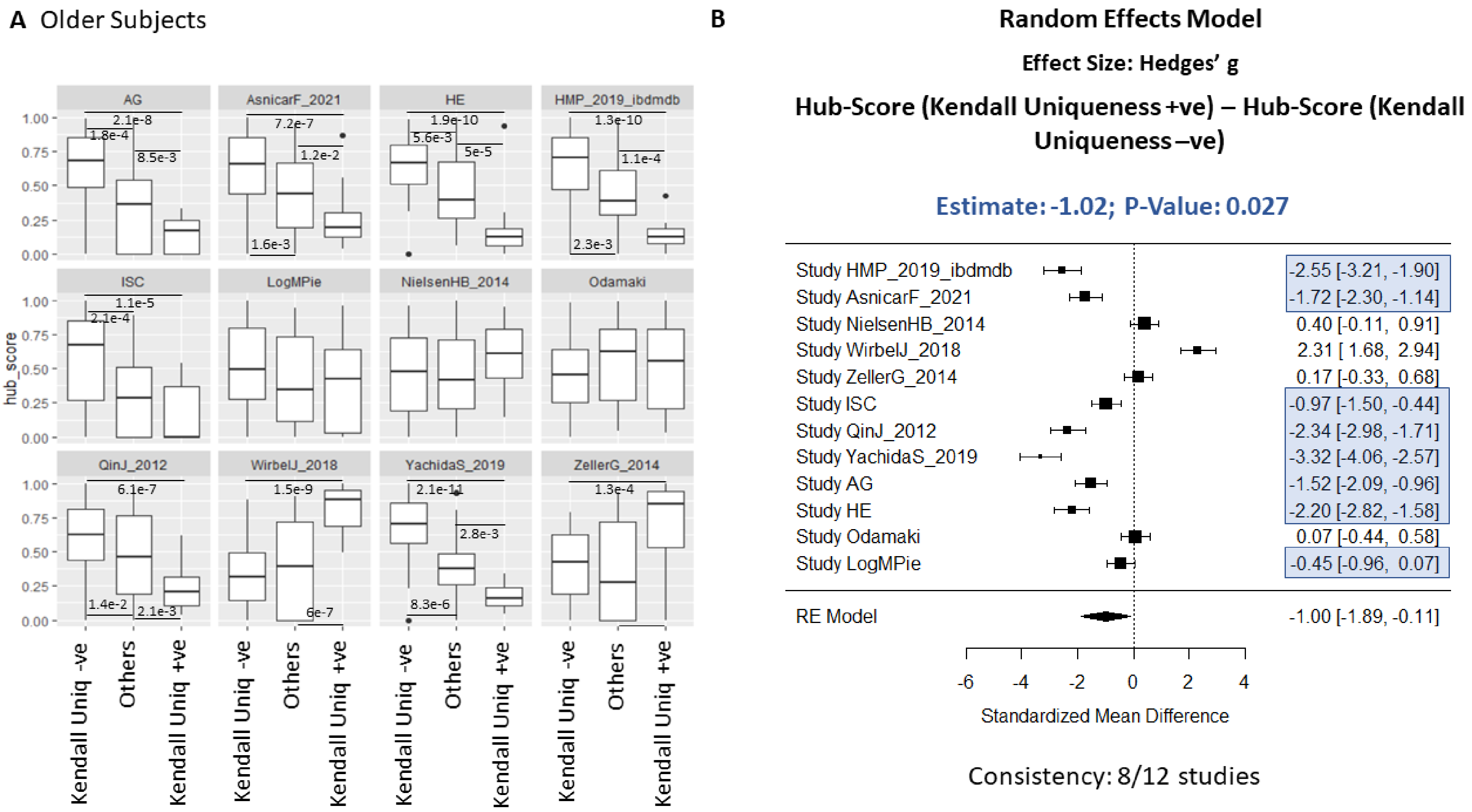
A Older Subjects
AG
AsnicarF_2021
HE
HMP_2019_ibdmdb
ISC
LogMPie
NielsenHB_2014
Odamaki
QinJ_2012
WirbelJ_2018
YachidaS_2019
ZellerG_2014
hub_score
1.00
0.75
0.50
0.25
0.00
2.1e-8
1.8e-4
8.5e-3
7.2e-7
1.2e-2
1.6e-3
1.9e-10
5.6e-3
5e-5
1.3e-10
1.1e-4
2.3e-3
1.1e-5
2.1e-4
6.1e-7
1.4e-2
2.1e-3
1.5e-9
6e-7
2.1e-11
2.8e-3
8.3e-6
1.3e-4
Kendall Uniq -ve
Others
Kendall Uniq +ve
B
Random Effects Model
Effect Size: Hedges' g
Hub-Score (Kendall Uniqueness +ve) – Hub-Score (Kendall Uniqueness –ve)
Estimate: -1.02; P-Value: 0.027
Study HMP_2019_ibdmdb -2.55 [-3.21, -1.90]
Study AsnicarF_2021 -1.72 [-2.30, -1.14]
Study NielsenHB_2014 0.40 [-0.11, 0.91]
Study WirbelJ_2018 2.31 [1.68, 2.94]
Study ZellerG_2014 0.17 [-0.33, 0.68]
Study ISC -0.97 [-1.50, -0.44]
Study QinJ_2012 -2.34 [-2.98, -1.71]
Study YachidaS_2019 -3.32 [-4.06, -2.57]
Study AG -1.52 [-2.09, -0.96]
Study HE -2.20 [-2.82, -1.58]
Study Odamaki 0.07 [-0.44, 0.58]
Study LogMPie -0.45 [-0.96, 0.07]
RE Model -1.00 [-1.89, -0.11]
-6
-4
-2
0
2
4
Standardized Mean Difference
Consistency: 8/12 studies

Supplementary Figure S12. **A.** Boxplot comparing the hub-scores of the three groups of species-level taxa (Kendall Uniqueness -ve, n=22; Kendall Uniqueness +ve, n=54, Others n=36) in the older-subject-specific co-occurrence networks obtained for the 12 different studies considered for the network-based analysis. Horizontal bars between boxes indicate the p-values of significance for the comparison within the different groups using dunns' test (two-sided) after adjusting for multiple comparisons using Benjamini-Hochberg. Individually, in seven out of the 12 study cohorts, the Kendall Uniqueness -ve species group exhibited significantly higher hub-score (at least with Q <= 0.05) than the Kendall Uniqueness +ve species group. This was further enforced in the Random Effect Model meta-analysis. **B.** Forest plot of the Random Effects Model meta-analysis indicating the significant hub-score difference between two groups of taxa in the older-subject-specific networks across the 12 studies. The summarized estimates of the model along p-value are also indicated. The significant negative effect size further indicates a higher betweenness for the Kendall Uniqueness -ve group as compared to the Kendall Uniqueness +ve taxa. Across 8 of the 12 studies, a negative effect size was observed. Boxes corresponding to the boxplots indicate the inter-quartile range (with the median indicated in bold) of the values and the upper and lower whiskers extend to +1.5 X interquartile range from the third quartile (upper whisker) or to -1.5 X interquartile range from the first quartile (lower whisker). The number of samples (or gut microbiome) (n) corresponding to the different studies are: AG:1023, AsnicarF_2021:127, HE:2434, HMP_2019_ibdmdb:117, ISC:202, LogMPie:51, NielsenHB_2014:68, NUAGE:610, Odamaki:116, QinJ_2012:71, WirbelJ_2018:67, YachidaS_2019:393, ZellerG_2014:109. In (B), Two-sided P-values for the Random Effect Model were computed using permutation tests of association for the summary effect sizes.

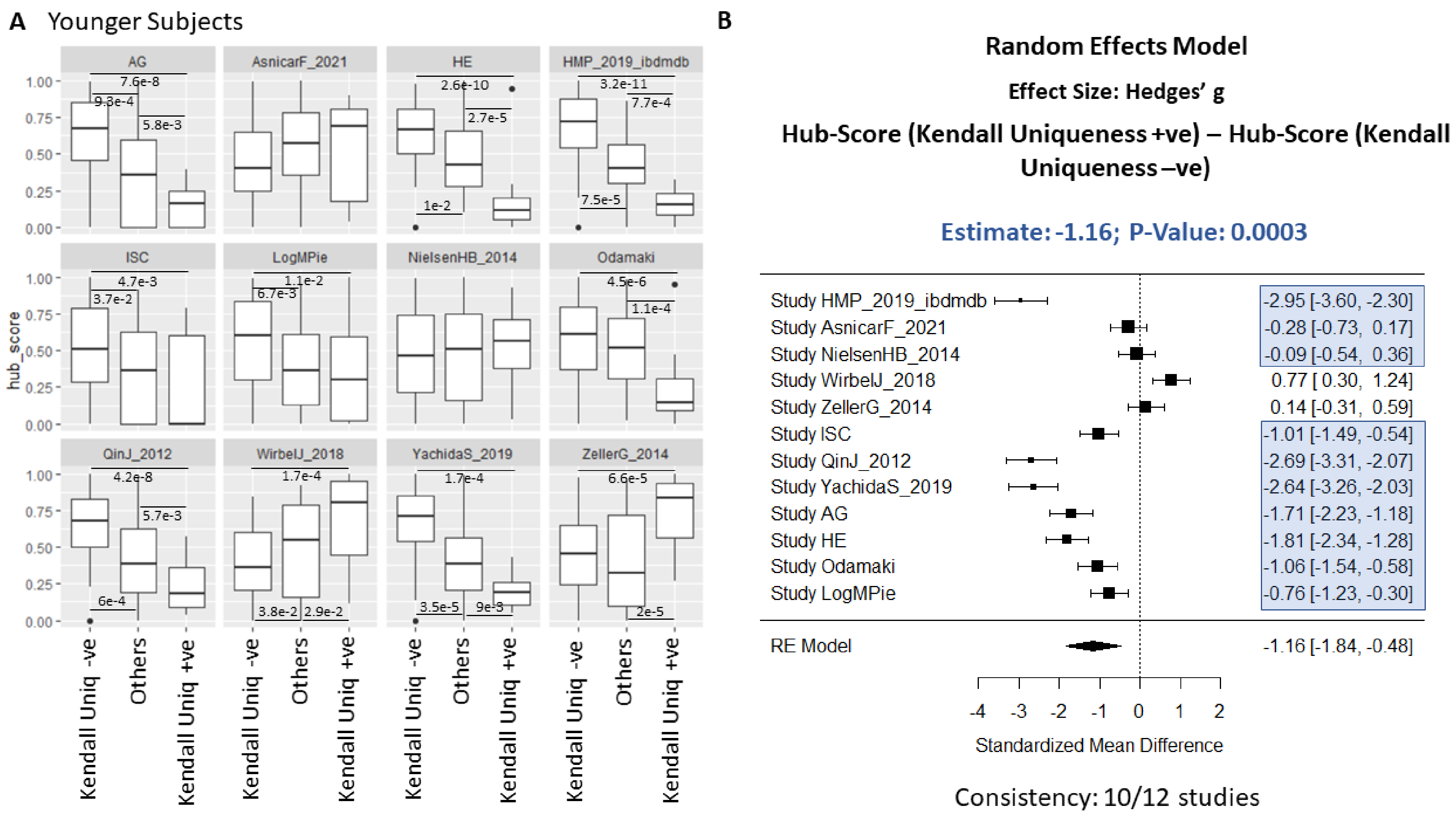
A Younger Subjects
AG
AsnicarF_2021
HE
HMP_2019_ibdmdb
ISC
LogMPie
NielsenHB_2014
Odamaki
QinJ_2012
WirbelJ_2018
YachidaS_2019
ZellerG_2014
hub_score
1.00
0.75
0.50
0.25
0.00
7.6e-8
9.3e-4
5.8e-3
2.6e-10
2.7e-5
1e-2
3.2e-11
7.7e-4
7.5e-5
4.7e-3
3.7e-2
1.1e-2
6.7e-3
4.5e-6
1.1e-4
4.2e-8
5.7e-3
6e-4
1.7e-4
3.8e-2
2.9e-2
1.7e-4
3.5e-5
9e-3
6.6e-5
2e-5
Kendall Uniq -ve
Others
Kendall Uniq +ve
B
Random Effects Model
Effect Size: Hedges' g
Hub-Score (Kendall Uniqueness +ve) – Hub-Score (Kendall Uniqueness –ve)
Estimate: -1.16; P-Value: 0.0003
Study HMP_2019_ibdmdb -2.95 [-3.60, -2.30]
Study AsnicarF_2021 -0.28 [-0.73, 0.17]
Study NielsenHB_2014 -0.09 [-0.54, 0.36]
Study WirbelJ_2018 0.77 [0.30, 1.24]
Study ZellerG_2014 0.14 [-0.31, 0.59]
Study ISC -1.01 [-1.49, -0.54]
Study QinJ_2012 -2.69 [-3.31, -2.07]
Study YachidaS_2019 -2.64 [-3.26, -2.03]
Study AG -1.71 [-2.23, -1.18]
Study HE -1.81 [-2.34, -1.28]
Study Odamaki -1.06 [-1.54, -0.58]
Study LogMPie -0.76 [-1.23, -0.30]
RE Model -1.16 [-1.84, -0.48]
-4 -3 -2 -1 0 1 2
Standardized Mean Difference
Consistency: 10/12 studies

Supplementary Figure S13. **A.** Boxplot comparing the hub-scores of the three groups of species-level taxa (Kendall Uniqueness -ve, n=22; Kendall Uniqueness +ve, n=54, Others n=36) in the young-specific co-occurrence networks obtained for the 12 different studies considered for the network-based analysis. Horizontal bars between boxes indicate the p-values of significance for the comparison within the different groups using dunns' test (two-sided) after adjusting for multiple comparisons using Benjamini-Hochberg. Individually, in eight out of the 12 study cohorts, the Kendall Uniqueness -ve species group exhibited significantly higher hub-score (at least with Q <= 0.05) than the Kendall Uniqueness +ve species group. This was further enforced in the Random Effect Model meta-analysis. **B.** Forest plot of the Random Effects Model meta-analysis indicating the significant hub-score difference between two groups of taxa in the young-specific networks across the 12 studies. The summarized estimates of the model along p-value are also indicated. The significant negative effect size further indicates a higher betweenness for the Kendall Uniqueness -ve group as compared to the Kendall Uniqueness +ve taxa. Across 10 of the 12 studies, a negative effect size was observed. Boxes corresponding to the boxplots indicate the inter-quartile range (with the median indicated in bold) of the values and the upper and lower whiskers extend to +1.5 X interquartile range from the third quartile (upper whisker) or to -1.5 X interquartile range from the first quartile (lower whisker). The number of samples (or gut microbiome) (n) corresponding to the different studies are AG:2789, AsnicarF_2021:971, HE:4575, HMP_2019_ibdmdb:729, ISC:262, LogMPie:823, NielsenHB_2014:326, Odamaki:190, QinJ_2012:271, WirbelJ_2018:58, YachidaS_2019:223, ZellerG_2014:47. In (B), Two-sided P-values for the Random Effect Model were computed using permutation tests of association for the summary effect sizes.

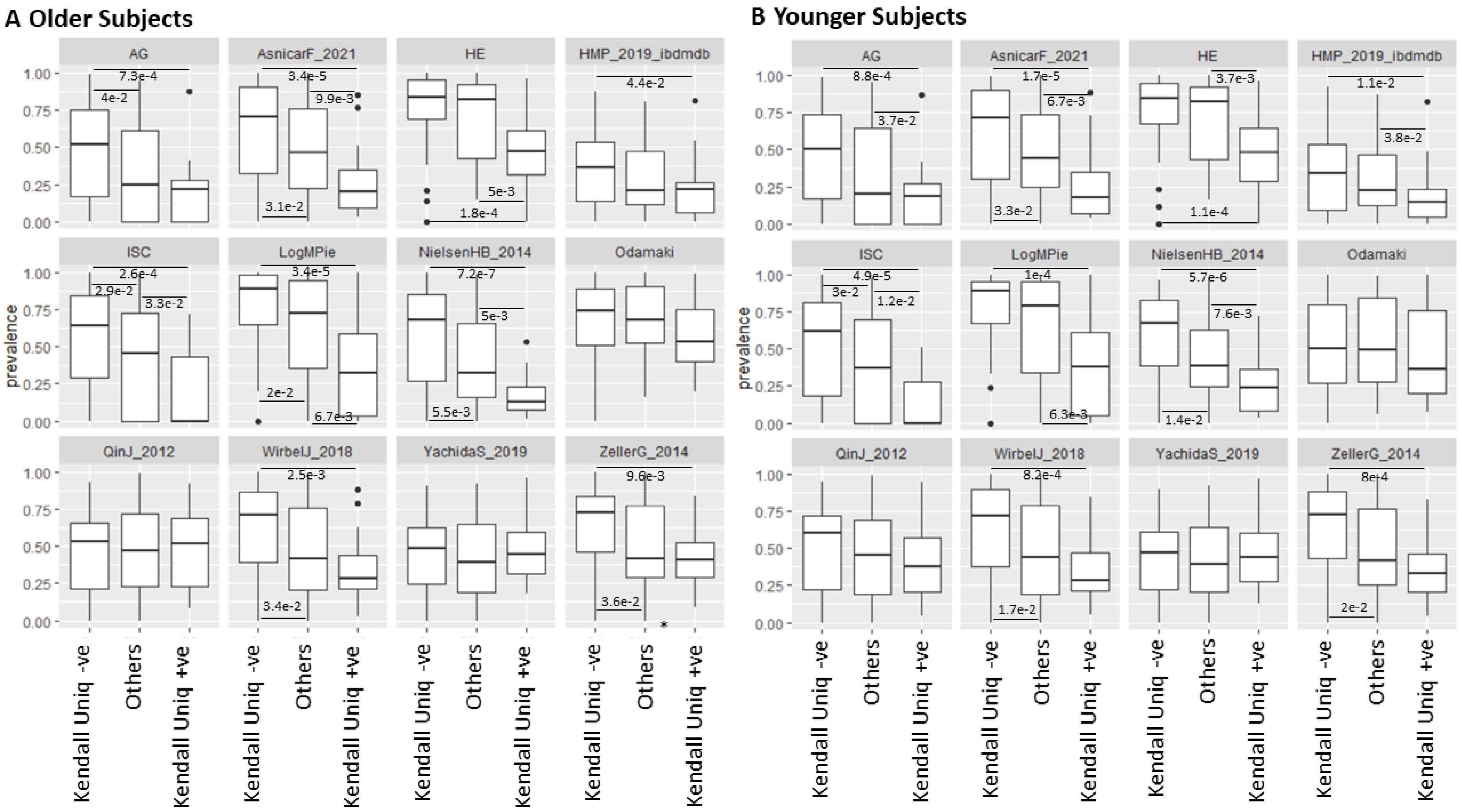
A Older Subjects
B Younger Subjects
AG
AsnicarF_2021
HE
HMP_2019_ibdmdb
ISC
LogMPie
NielsenHB_2014
Odamaki
QinJ_2012
WirbelJ_2018
YachidaS_2019
ZellerG_2014
prevalence
1.00
0.75
0.50
0.25
0.00
Kendall Uniq -ve
Others
Kendall Uniq +ve
7.3e-4
4e-2
3.4e-5
9.9e-3
3.1e-2
5e-3
1.8e-4
4.4e-2
2.6e-4
2.9e-2
3.3e-2
2e-2
6.7e-3
7.2e-7
5.5e-3
2.5e-3
3.4e-2
9.6e-3
3.6e-2
8.8e-4
3.7e-2
1.7e-5
6.7e-3
3.3e-2
3.7e-3
1.1e-4
1.1e-2
3.8e-2
4.9e-5
3e-2
1.2e-2
1e-4
6.3e-3
5.7e-6
7.6e-3
1.4e-2
8.2e-4
1.7e-2
8e-4
2e-2

Supplementary Figure S14. Boxplots comparing the prevalence of the taxa belonging to the three species-level groups (Kendall Uniqueness -ve, n=22; Kendall Uniqueness +ve, n=54, Others n=36) across the 12 different studies considered for the network-based analysis by separately considering the gut microbiome datasets obtained from **A)** older individuals (age >= 60 years) and B) Younger individuals with age <60 years. Horizontal bars between boxes indicate the p-values of significance for the comparison within the different groups using dunns' test (two-sided) after adjusting for multiple comparisons using Benjamini-Hochberg. Boxes corresponding to the boxplots indicate the inter-quartile range (with the median indicated in bold) of the values and the upper and lower whiskers extend to +1.5 X interquartile range from the third quartile (upper whisker) or to -1.5 X interquartile range from the first quartile (lower whisker). For the gut microbiomes from older subjects, the number of samples (or gut microbiome) (n) corresponding to the different studies are: AG:1023, AsnicarF_2021:127, HE:2434, HMP_2019_ibdmdb:117, ISC:202, LogMPie:51, NielsenHB_2014:68, NUAGE:610, Odamaki:116, QinJ_2012:71, WirbelJ_2018:67, YachidaS_2019:393, ZellerG_2014:109. For the gut microbiomes from younger subjects, the number of samples (or gut microbiome) (n) corresponding to the different studies are AG:2789, AsnicarF_2021:971, HE:4575, HMP_2019_ibdmdb:729, ISC:262, LogMPie:823, NielsenHB_2014:326, Odamaki:190, QinJ_2012:271, WirbelJ_2018:58, YachidaS_2019:223, ZellerG_2014:47. In (B), Two-sided P-values for the Random Effect Model were computed using permutation tests of association for the summary effect sizes.

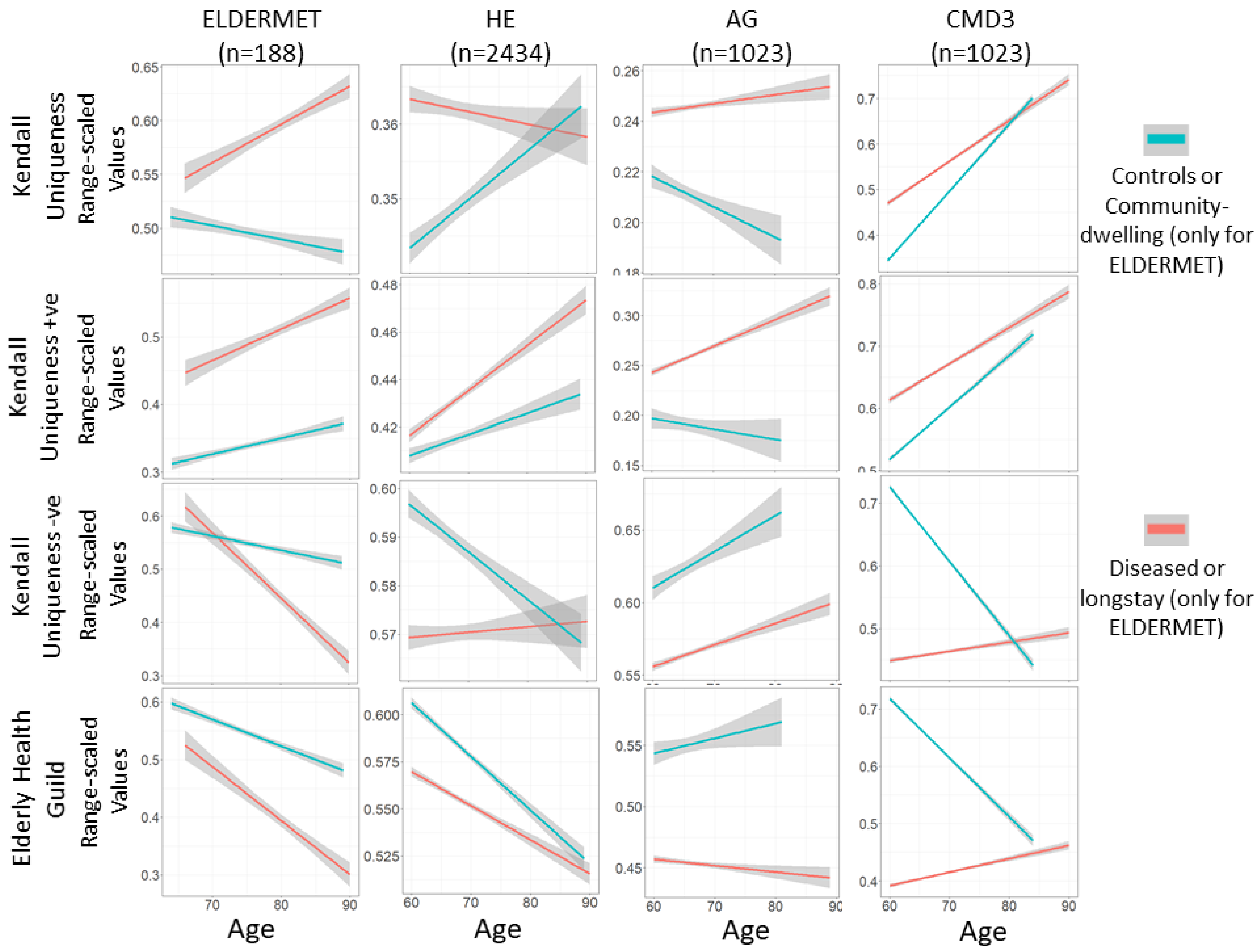
ELDERMET
(n=188)
HE
(n=2434)
AG
(n=1023)
CMD3
(n=1023)
Kendall Uniqueness
Kendall Uniqueness +ve
Kendall Uniqueness -ve
Elderly Health Guild
Range-scaled Values
Age
Controls or Community-dwelling (only for ELDERMET)
Diseased or longstay (only for ELDERMET)

Supplementary Figure S15. Variation of four key health-linked microbiome properties with age within the control and unhealthy phenotypes of older people for the four major cohorts. While the rate of variation of these properties was not always consistent across cohorts., the unhealthy subjects always have higher values of Kendall Uniqueness and disease-associated Kendall Uniqueness positive taxa and lower values of Health associated Kendall Uniqueness negative taxa and the select healthy aging associated taxonomic guild compared to the controls. For both the plots, bold lines indicate the mean regression line for each of the associations, the shaded regions (in grey) corresponding to each line indicate their confidence intervals (+/- standard errors).
