# Supplementary material for: Toward an improved definition of a healthy microbiome for healthy aging
Source: Nat Aging. 2022 Nov 17;2(11):1054–69. doi: 10.1038/s43587-022-00306-9 (PMC10154212; doi:10.1038/s43587-022-00306-9)
Supplement: Supplementary file 2 — Reporting Summary [file 43587_2022_306_MOESM2_ESM.pdf]

nature portfolio

Corresponding author(s): Paul W O'Toole
Last updated by author(s): Oct 4, 2022

Reporting Summary

Nature Portfolio wishes to improve the reproducibility of the work that we publish. This form provides structure for consistency and transparency in reporting. For further information on Nature Portfolio policies, see our Editorial Policies and the Editorial Policy Checklist.

Please do not complete any field with "not applicable" or n/a. Refer to the help text for what text to use if an item is not relevant to your study.
For final submission: please carefully check your responses for accuracy; you will not be able to make changes later.

Statistics

For all statistical analyses, confirm that the following items are present in the figure legend, table legend, main text, or Methods section.

n/a	Confirmed	
☐	☒	The exact sample size (*n*) for each experimental group/condition, given as a discrete number and unit of measurement
☒	☐	A statement on whether measurements were taken from distinct samples or whether the same sample was measured repeatedly
☐	☒	The statistical test(s) used AND whether they are one- or two-sided<br>*Only common tests should be described solely by name; describe more complex techniques in the Methods section.*
☐	☒	A description of all covariates tested
☐	☒	A description of any assumptions or corrections, such as tests of normality and adjustment for multiple comparisons
☐	☒	A full description of the statistical parameters including central tendency (e.g. means) or other basic estimates (e.g. regression coefficient) AND variation (e.g. standard deviation) or associated estimates of uncertainty (e.g. confidence intervals)
☐	☒	For null hypothesis testing, the test statistic (e.g. *F*, *t*, *r*) with confidence intervals, effect sizes, degrees of freedom and *P* value noted<br>*Give P values as exact values whenever suitable.*
☒	☐	For Bayesian analysis, information on the choice of priors and Markov chain Monte Carlo settings
☒	☐	For hierarchical and complex designs, identification of the appropriate level for tests and full reporting of outcomes
☐	☒	Estimates of effect sizes (e.g. Cohen's *d*, Pearson's *r*), indicating how they were calculated

Our web collection on statistics for biologists contains articles on many of the points above.

Software and code

Policy information about availability of computer code

Data collection	All data utilized as part of the study were publicly available either as part of sequence repositories such as European Nucleotide Archive (ENA) and DNA Databank of Japan (DDBJ) or as pre-processed profiles from curatedMetagenomicData and figshare. Please refer to the detailed Data Availability Statement below for these details. All the data corresponding to the NU-AGE data set utilized in the current study are uploaded to this github repository. or curatedMetagenomicData3 repository, the taxonomic and pathway profiles were already available and hence were downloaded and directly utilized for the current study. The sequence data for each of the individual study collated as part of the curatedMetagenomicData3 (CMD3) are publicly available and the corresponding accession numbers can be obtained by downloading the repository at: https://waldronlab.io/curatedMetagenomicData/ . For the American Gut (AG) project, the filtered, bloom removed OTU biom files and the corresponding metadata were already available at figshare with reference IDs 6137192 and 6137315, respectively (11, 36, 37). These profiles were utilized for the steps of this analyses. For He et al and LogMPie cohort, the sequence data was available at the European Nucleotide Archive (ENA) (https://www.ebi.ac.uk/ena/) at accession numbers PRJEB18535 and PRJEB25642, respectively, and the metadata available as part of the original publications (39, 40). For Odamaki et al, the sequence data was available at the DDBJ under the accession under accession number DRA004160 and the metadata was obtained from the corresponding publication (16). For the four studies comprising the Irish Shotgun cohorts, the sequence data were already publicly available at the ENA under the accessions PRJEB20054 (Cronin et al) (12), PRJEB15388 (Barton et al) (13), PRJEB42304 (Jeffery et al) (14), and PRJEB37017 (Ghosh et al) (1). The starting data and the processed profiles for the NU-AGE data resource as well as the minimum starting data for each repository that are necessary to interpret, verify and extend the research in the article are available at: https://github.com/tsg-microbiome/AgeMetaAnalysis.
Data analysis	All previously published computer programs that were used in the current study have been described in the Methods section and referred to in the manuscript. All analysis codes that were created as part of this study have been uploaded to GitHub at: https://github.com/tsg-microbiome/AgeMetaAnalysis. All the data corresponding to the NU-AGE data set utilized in the current study are uploaded to this github repository. The different software packages and their versions are listed below: R version 4.1.0, Cytoscape v3.8.0, Different modules with R

package: curatedMetagenomicData (v3.0.10), metafor (v3.8.1), vegan (v2.5.7), ade4 (v1.7.17), psych (v2.1.19), gplots (3.1.1), RColorBrewer (v1.1.2), metap (v1.8), igraph (v1.3.4), dplyr (v1.0.10), effsize (v0.8.1), MASS (v7.3.54), sfsmisc (v1.1.12), pcaPP (v1.9.74), dendextend (v1.16.0), ccrepe (1.28.0), compositions (v2.0.4)

For manuscripts utilizing custom algorithms or software that are central to the research but not yet described in published literature, software must be made available to editors and reviewers. We strongly encourage code deposition in a community repository (e.g. GitHub). See the Nature Portfolio guidelines for submitting code & software for further information.

Data

Policy information about availability of data

All manuscripts must include a data availability statement. This statement should provide the following information, where applicable:

- Accession codes, unique identifiers, or web links for publicly available datasets
- A description of any restrictions on data availability
- For clinical datasets or third party data, please ensure that the statement adheres to our policy

The study is a meta-analysis of seven major data resources, the sequence data for four of the data resources (with the exception of NU-AGE) are publicly available. For curatedMetagenomicData3 repository, the taxonomic and pathway profiles were already available and hence were downloaded and directly utilized for the current study. The sequence data for each of the individual study collated as part of the curatedMetagenomicData3 (CMD3) are publicly available and the corresponding accession numbers can be obtained by downloading the repository at: https://waldronlab.io/curatedMetagenomicData/ . For the American Gut (AG) project, the filtered, bloom removed OTU biom files and the corresponding metadata were already available at figshare with reference IDs 6137192 and 6137315, respectively (11, 36, 37). These profiles were utilized for the steps of this analyses. For He et al and LogMPie cohort, the sequence data was available at the European Nucleotide Archive (ENA) (https://www.ebi.ac.uk/ena/) at accession numbers PRJEB18535 and PRJEB25642, respectively, and the metadata available as part of the original publications (39, 40). For Odamaki et al, the sequence data was available at the DDBJ under the accession under accession number DRA004160 and the metadata was obtained from the corresponding publication (16). For the four studies comprising the Irish Shotgun cohorts, the sequence data were already publicly available at the ENA under the accessions PRJEB20054 (Cronin et al) (12), PRJEB15388 (Barton et al) (13), PRJEB42304 (Jeffery et al) (14), and PRJEB37017 (Ghosh et al) (1). The starting data and the processed profiles for the NU-AGE data resource as well as the minimum starting data for each repository that are necessary to interpret, verify and extend the research in the article are available at: https://github.com/tsg-microbiome/AgeMetaAnalysis. All the data corresponding to the NU-AGE data set utilized in the current study are uploaded to this github repository. The explanations for the different data resources are provided in the README.md file of this github repository.

Human research participants

Policy information about studies involving human research participants and Sex and Gender in Research.

Reporting on sex and gender	The study utilizes meta-analysis on publicly available deanonymized data and does not collect data from human participants as part of this study. The details on protocols involving different aspects of the human study participants are described in the original studies (which have been referred to in this study).
Population characteristics	not applicable. As described in the previous response, this is a meta-analysis. The details can be obtained from the original studies
Recruitment	not applicable. As described in the previous response, this is a meta-analysis. The details can be obtained from the original studies
Ethics oversight	not applicable. As described in the previous response, this is a meta-analysis. The details can be obtained from the original studies

Note that full information on the approval of the study protocol must also be provided in the manuscript.

Field-specific reporting

Please select the one below that is the best fit for your research. If you are not sure, read the appropriate sections before making your selection.

☒ Life sciences ☐ Behavioural & social sciences ☐ Ecological, evolutionary & environmental sciences

For a reference copy of the document with all sections, see nature.com/documents/nr-reporting-summary-flat.pdf

Life sciences study design

All studies must disclose on these points even when the disclosure is negative.

Sample size	Since the current study is a meta-analysis of several publicly available datasets, no statistical method was used to predetermine sample size in this study. We have attempted to include all data from each of the available datasets. Wherever applicable, we have described the criteria used to select the specific subsets of studies.Wherever possible, the sizes of the different studies in terms of the number of participants have been indicated as part of various main and supplementary tables as well as summarized in the Results and Methods section. We have also explained the same in the section 'Statistics and Reproducibility' in the Methods.

Data exclusions	Data wherever excluded have been indicated in the Methods and Results section. We have described the criteria used to select the specific subsets of studies. We have also explained the same in the section 'Statistics and Reproducibility' in the Methods.
Replication	The current study adopts meta-analysis approach that inherently looks for reproducibility of findings across multiple studies. For most meta-analysis, we also checked how consistently was a finding reproduced across studies and utilized specific thresholds to identify and report consistent as well as significant findings.
Randomization	This is not clinical trial. Hence, no randomization was used.
Blinding	This is not clinical trial. Hence, no blinding was performed.

Reporting for specific materials, systems and methods

We require information from authors about some types of materials, experimental systems and methods used in many studies. Here, indicate whether each material, system or method listed is relevant to your study. If you are not sure if a list item applies to your research, read the appropriate section before selecting a response.

Materials & experimental systems

n/a	Involved in the study	
☒	☐	Antibodies
☒	☐	Eukaryotic cell lines
☒	☐	Palaeontology and archaeology
☒	☐	Animals and other organisms
☒	☐	Clinical data
☒	☐	Dual use research of concern
☒	☐	

Methods

n/a	Involved in the study	
☒	☐	ChIP-seq
☒	☐	Flow cytometry
☒	☐	MRI-based neuroimaging

This checklist template is licensed under a Creative Commons Attribution 4.0 International License, which permits use, sharing, adaptation, distribution and reproduction in any medium or format, as long as you give appropriate credit to the original author(s) and the source, provide a link to the Creative Commons license, and indicate if changes were made. The images or other third party material in this article are included in the article's Creative Commons license, unless indicated otherwise in a credit line to the material. If material is not included in the article's Creative Commons license and your intended use is not permitted by statutory regulation or exceeds the permitted use, you will need to obtain permission directly from the copyright holder. To view a copy of this license, visit http://creativecommons.org/licenses/by/4.0/
