# Supplementary figures and images for: Toward an improved definition of a healthy microbiome for healthy aging
Source: Nat Aging. 2022 Nov 17;2(11):1054–69. doi: 10.1038/s43587-022-00306-9 (PMC10154212; doi:10.1038/s43587-022-00306-9)

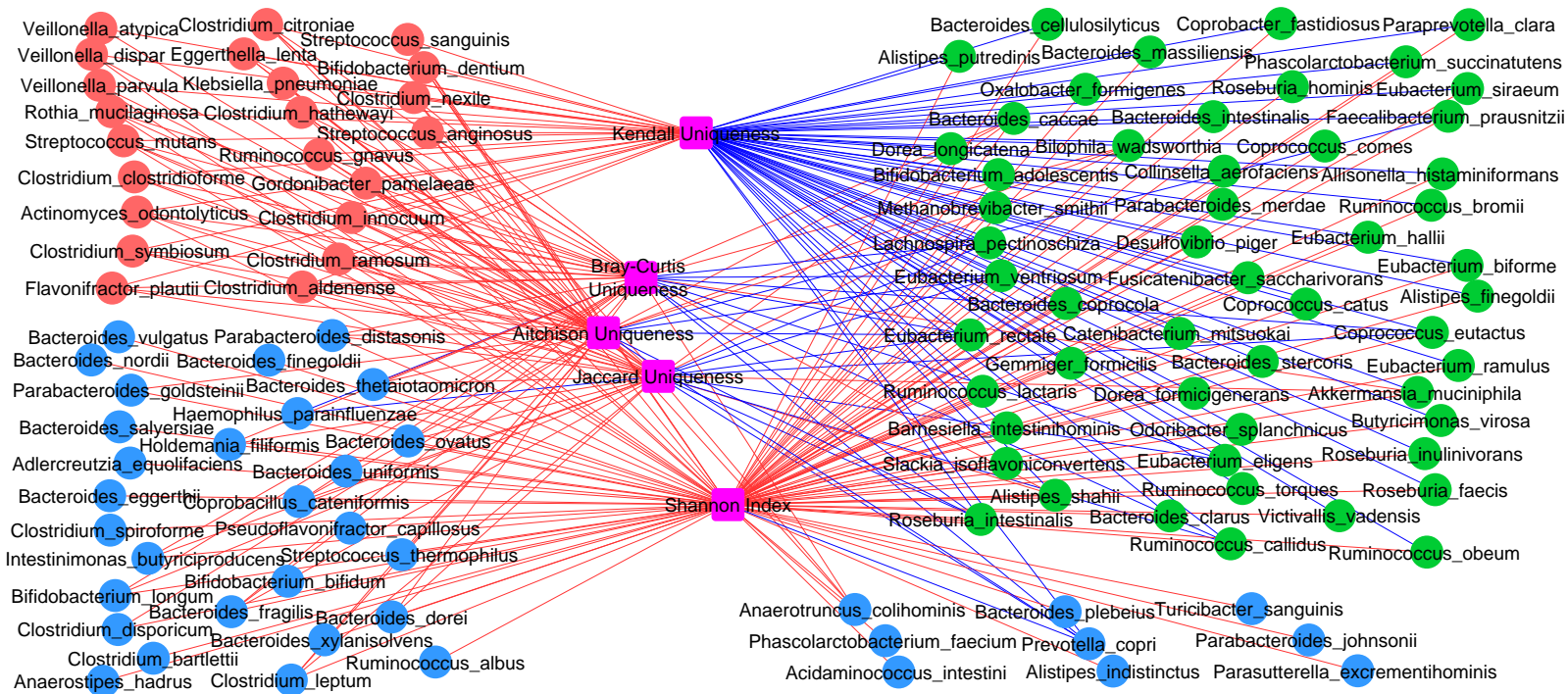

Supplement: Source Data Fig. 2. — Individual high-resolution editable network picture for Fig. 2. [file 43587_2022_306_MOESM11_ESM.pdf]

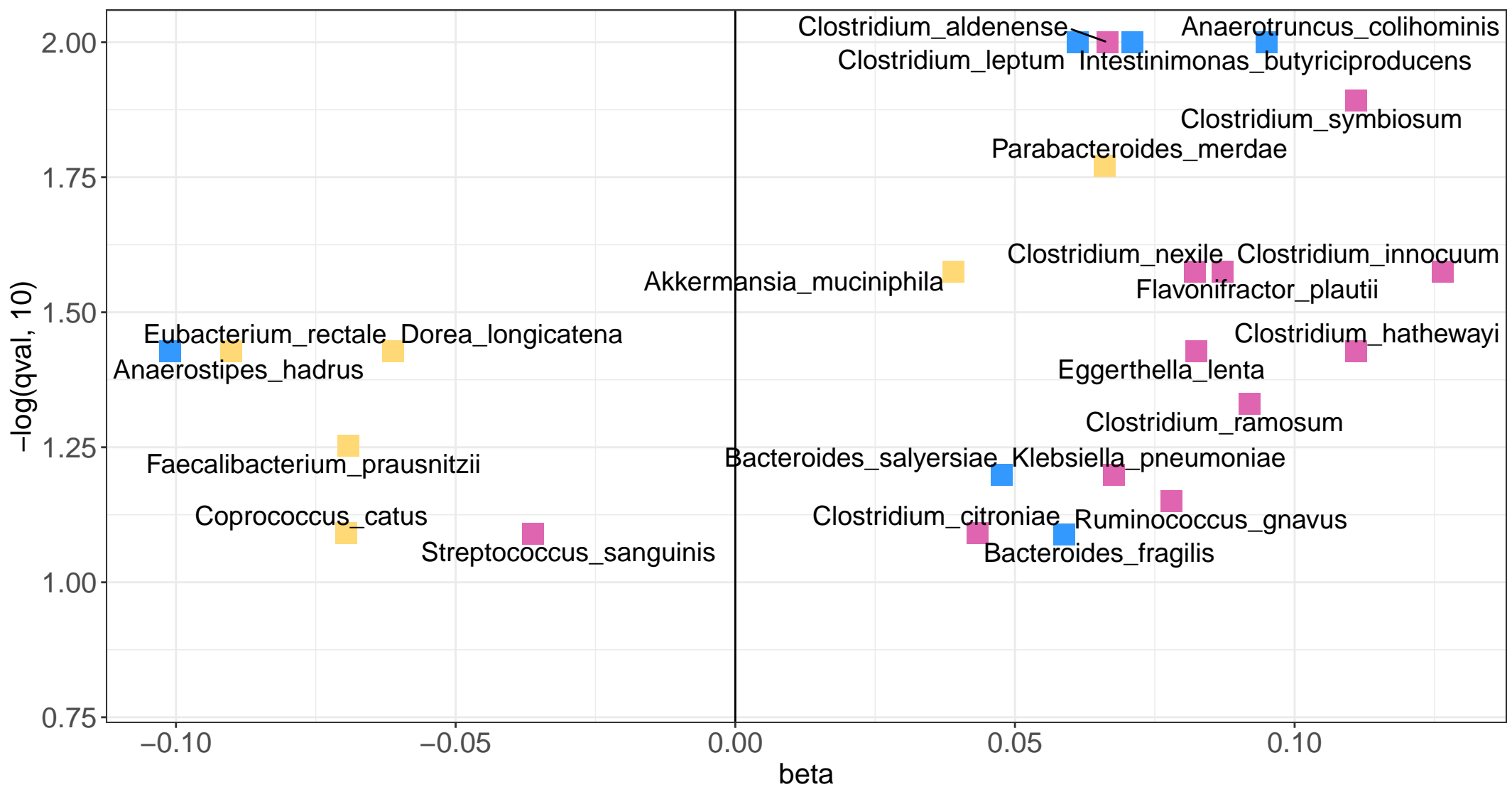

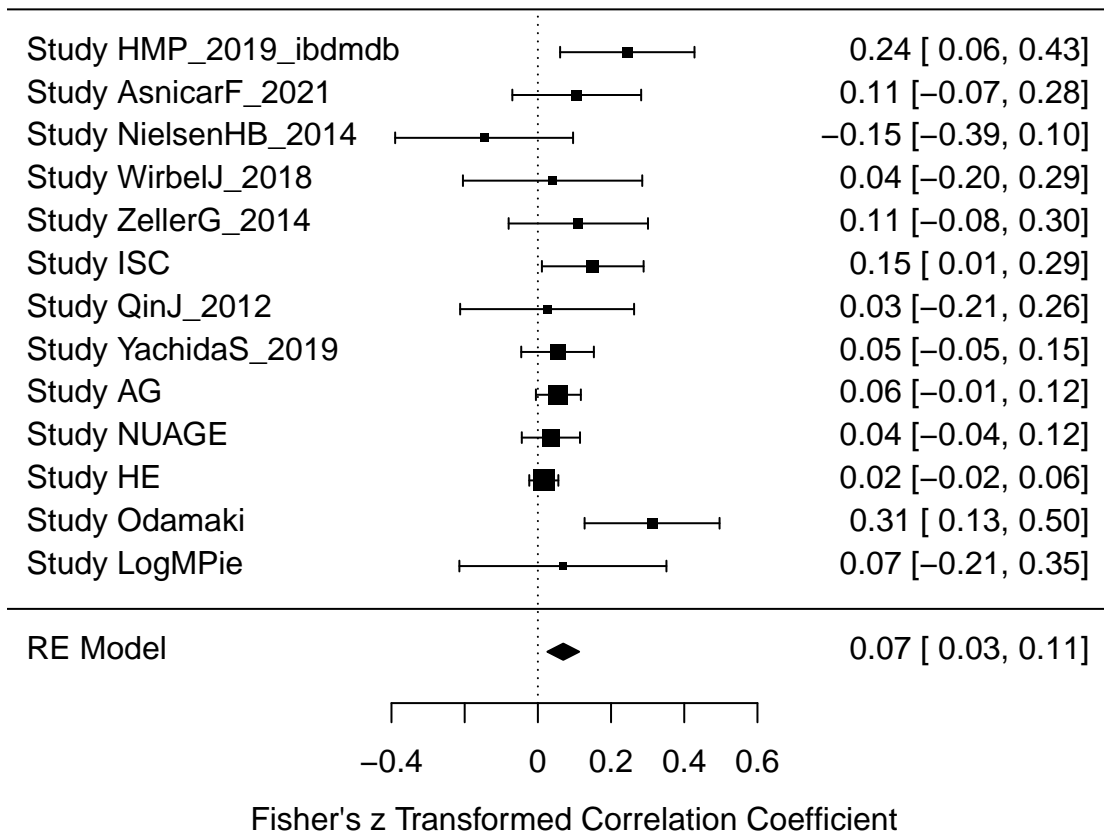

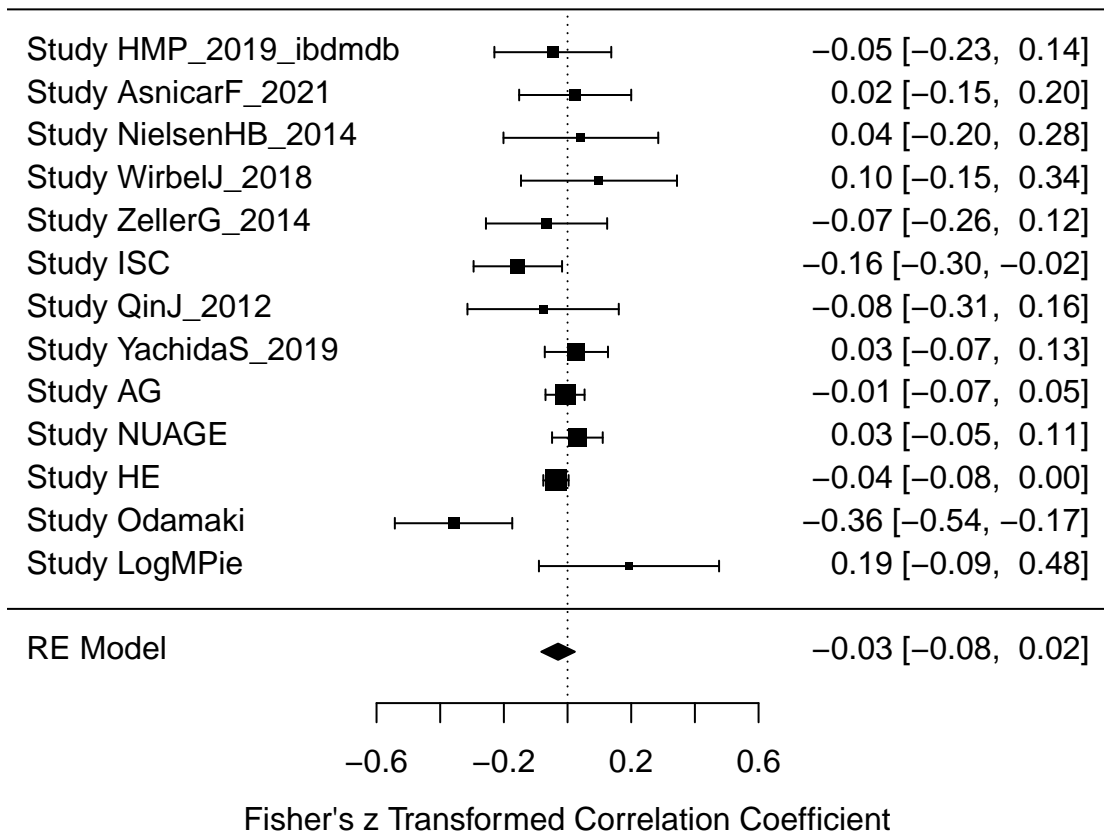

Supplement: Source Data Fig. 3. — Individual high-resolution editable images for Fig. 3a,b. [file 43587_2022_306_MOESM12_ESM.pdf]

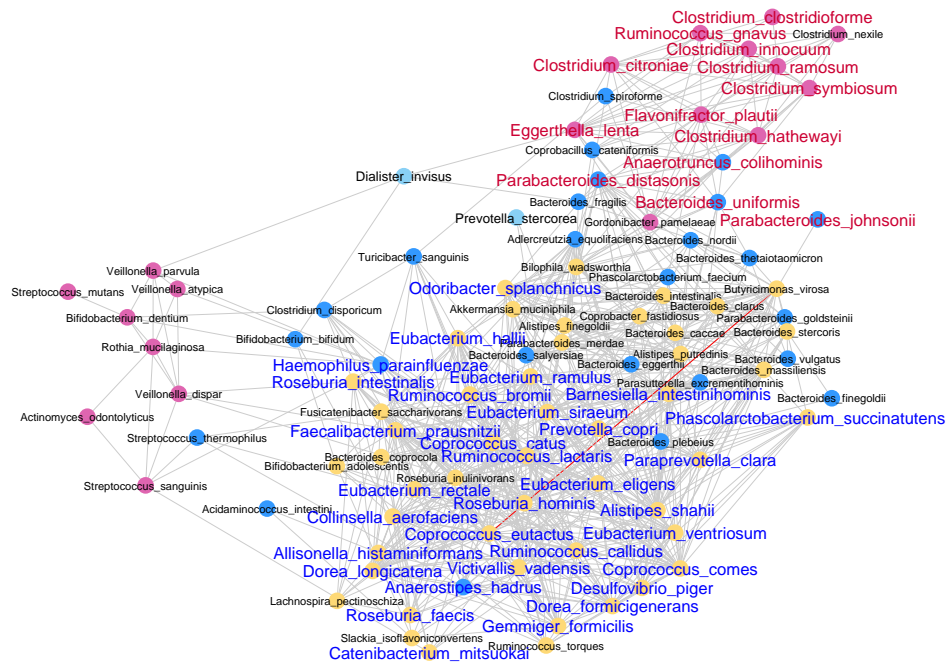

Supplement: Source Data Fig. 5. — Individual high-resolution editable network pictures for Fig. 5. [file 43587_2022_306_MOESM14_ESM.pdf]

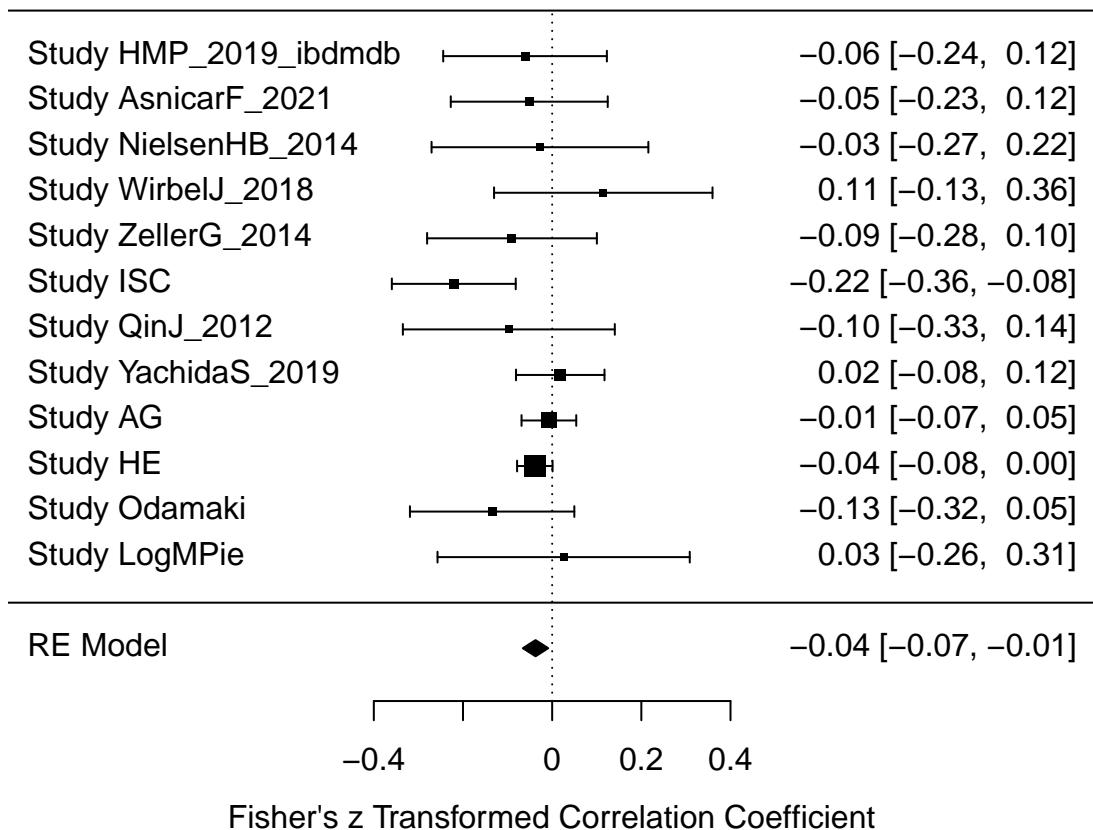

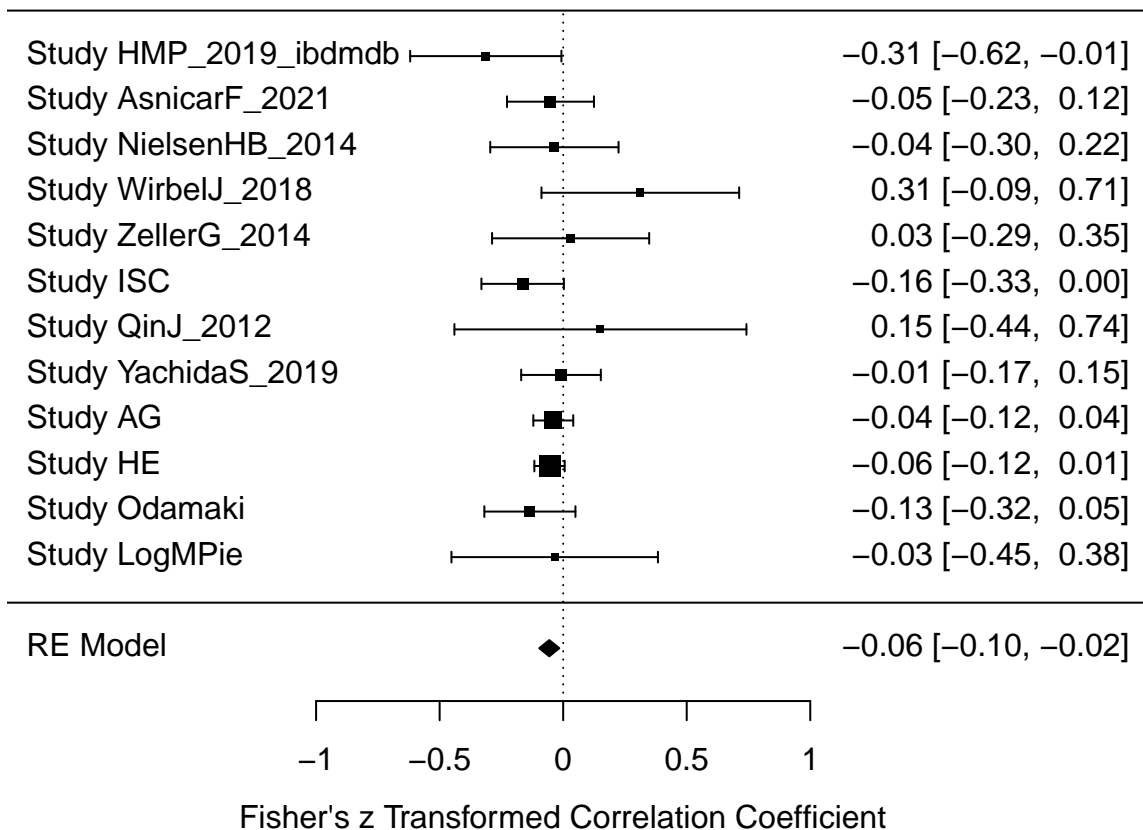

Supplement: Source Data Extended Data Fig. 9. — Individual high-resolution editable forest plots for Extended Data Fig. 9a,b. [file 43587_2022_306_MOESM22_ESM.pdf]

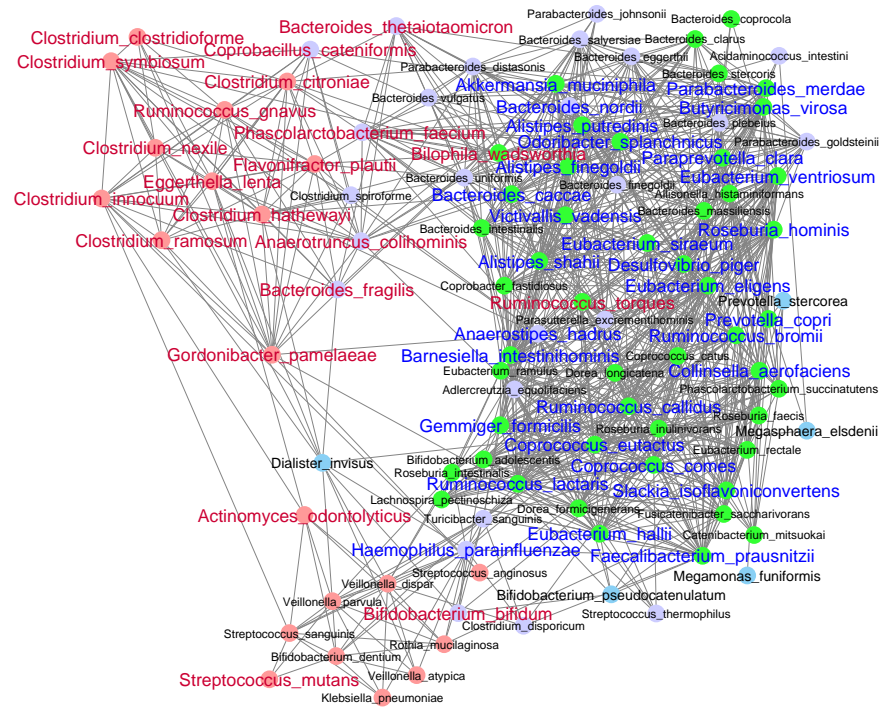

Supplement: Source Data Extended Data Fig. 10. — Individual high-resolution editable network pictures for Fig. 5. [file 43587_2022_306_MOESM23_ESM.pdf]
